# Selection Bias in Hybrid Randomized Controlled Trials Using External Controls: A Simulation Study

**DOI:** 10.1002/sim.70819

**Published:** 2026-11-13

**Authors:** Han Chang Chiam, Franz König, Martin Posch

**Affiliations:** ^1^ Center for Medical Data Science Medical University of Vienna Vienna Austria

**Keywords:** external controls, historical control selection bias, hybrid RCTs, prespecification, robust MAP, test‐then‐pool

## Abstract

Hybrid randomized controlled trials (hybrid RCTs) integrate external control data, such as historical or concurrent data, with data from randomized trials. While numerous frequentist and Bayesian methods, such as the test‐then‐pool and the meta‐analytic‐predictive prior, have been developed to account for potential disagreement between the external control and randomized data, they cannot ensure strict Type I error rate control. However, these methods can reduce biases stemming from systematic differences between external controls and trial data. A critical yet underexplored issue in hybrid RCTs is the prespecification of external data to be used in the analysis. The validity of statistical conclusions in hybrid RCTs depends on the assumption that external control selection is independent of historical trial outcomes. In practice, historical data may be accessible during the planning stage, potentially influencing important decisions, such as which historical datasets to include or the sample size of the prospective part of the hybrid trial, thus introducing bias. Such data‐driven design choices can be an additional source of bias, which can occur even when historical and prospective controls are exchangeable. Through a simulation study, we quantify the bias introduced by outcome‐dependent selection of historical controls in hybrid RCTs using both Bayesian and frequentist approaches, and discuss potential strategies to mitigate this bias. Our scenarios consider variability and time trends in the historical studies, distributional shifts between historical and prospective control groups, sample sizes and allocation ratios, as well as the number of studies included. The impact of different hypothetical selection mechanisms for selecting external controls is demonstrated using a clinical trial example.

**Abbreviations**
ELIR: expected local‐information‐ratio
EMA: European Medicines Agency
ESS: effective sample size
FDA: United States Food and Drug Administration
hybrid RCTs: hybrid randomized controlled trials
ICH: International Council for Harmonisation of Technical Requirements for Pharmaceuticals for Human Use
MAP: meta‐analytic‐predictive
MHRA: United Kingdom Medicines and Healthcare products Regulatory Agency
ODS: outcome‐dependent selection
RCT: randomized controlled trial
RD: risk difference
RMSE: root mean square error
TTP: test‐then‐pool

## 1 Introduction

Randomized controlled trials (RCTs) are the gold standard for establishing the safety and efficacy of new therapies. In rare or life‐threatening diseases, conventional RCTs are difficult to conduct because they can require large sample sizes, long recruitment periods, and randomization to a comparator arm that may appear less acceptable to eligible patients when treatment options are limited. Recent open‐science initiatives and data‐sharing portals [1, 2, 3] now give investigators access to patient data from completed trials, disease registries, and electronic health records. These resources motivate the integration of external information in the decision making for RCTs to increase statistical efficiency, reduce the required sample size, and accelerate the conduct of RCTs. Hybrid randomized controlled trials (hybrid RCTs) achieve this by supplementing the concurrently randomized control arm with external controls, where the control condition may be placebo, standard of care, best supportive care, or an active comparator, depending on the clinical setting. Compared to single‐arm trials and thresholding approaches [4], they preserve certain benefits of randomization and increase the chance that participants receive the novel treatment with unequal randomization [5, 6]. If the observed randomized and external controls differ substantially, the analysis can revert to using only the internal controls, thereby mitigating bias in treatment comparisons [7, 8, 9].

Because external controls can introduce bias, the International Council for Harmonisation of Technical Requirements for Pharmaceuticals for Human Use (ICH) E10 guideline states that external controls are acceptable “only in limited circumstances”, typically when a large, clearly attributable treatment effect is expected in a disease with a well‐characterized natural history [10]. The United States Food and Drug Administration (FDA) guidance on rare diseases also mentions the same principles for serious conditions with predictable outcomes [11].

The use of external data in clinical trials has received increasing attention in the past decades, and regulatory agencies have released several guidance documents in this context (EMA 2018 [12]; FDA 2023a, b [13, 14]). Two very recent drafts reinforce this trajectory: the United Kingdom Medicines and Healthcare products Regulatory Agency (MHRA) 2025 draft guideline on real‐world‐data external‐control arms demands that sponsors prespecify and lock the external comparator set, and Section 5.3 of the ICH E20 draft guideline on adaptive designs similarly emphasizes the prospective justification of any external data incorporated into an adaptive or hybrid design [15, 16]. The FDA draft guidance on Bayesian methodology in clinical trials also addresses the use of external information, including historical controls, for constructing informative priors. Its section on the identification and review of available external information is closely relevant to the selection issues examined in this paper [17]. Related issues were also discussed at two recent European Medicines Agency (EMA) workshops: one on the use of Bayesian statistics in clinical development, including metrics to assess Bayesian trials and use cases of historical borrowing with Bayesian methods [18], and one on the use of external controls for evidence generation in regulatory decision‐making [19]. A planned EMA reflection paper on Bayesian statistics in clinical development will outline the required information and justification for employing Bayesian methods, including prespecification and reporting of data sources. Further EMA guidance on Bayesian methods and external controls is under development [20, 21]. However, guidance specific to the use of external controls to augment a randomized concurrent control arm is still limited. A recent scoping review by Bofill Roig et al. [22] identified 37 EMA, FDA, and ICH guidance documents that mentioned non‐concurrent or external controls; the most common circumstances for the use of external controls in the identified guideline documents were “rare disease” or “indication specific” concerns followed by “large treatment effect” and “ethical concerns.”

The use of external controls relies on the exchangeability assumption whereby the distribution of potential outcomes in the external control group is similar to that in the randomized control group, possibly conditional on relevant baseline covariates. A well‐acknowledged challenge is the risk of bias when external and internal data are not exchangeable, which may result in a so called prior‐data conflict. Existing borrowing methods differ mainly in how they regulate the contribution of external information. In fixed borrowing approaches, for example power priors with prespecified discount parameters, the external contribution is determined a priori [23]. In dynamic borrowing approaches, the external contribution is down‐weighted or discounted based on observed compatibility between external and internal controls, often assessed through outcome differences, likelihood‐based measures or commensurability parameters. For example, adaptive power priors update the discount applied to the external likelihood, meta‐analytic approaches summarize historical controls to construct a predictive prior for the current control response, and model averaging approaches classify exchangeable and non‐exchangeable components via mixture models in the Bayesian framework [24, 25, 26]. In the frequentist setting, test‐then‐pool (TTP) pools external and internal controls if a preliminary compatibility test does not indicate disagreement, whereas adaptive lasso and recently developed conformal selective‐borrowing methods select or weight external information using outcome comparability, baseline covariates, or both [27, 28, 29]. Extensions of these borrowing frameworks may further incorporate regression adjustment, matching, weighting, or related tools such as propensity scores to improve comparability with respect to measured baseline covariates. These approaches have recently been reviewed by Zhu et al. [30]. A comprehensive review of these approaches is beyond the scope of this study.

Some methods use external control data during trial conduct to determine whether or how much external information should be incorporated [27, 31, 32]. However, these approaches still require a prespecified set of candidate external data sources. The candidate pool itself may already have been selected with knowledge of historical data outcomes, and subsequent borrowing or compatibility assessments cannot remove bias introduced by this selection. The integrity of statistical conclusions in hybrid RCTs depends on the assumption that external control selection is independent of the outcomes of the historical control data. In practice, historical data may be available during the planning stage and may influence which datasets are included or how the prospective part of the trial is sized, subsequently introducing bias. While the appropriateness of external data can be assessed using Pocock's criteria and has been discussed in several studies [5, 33, 34, 35, 36], the process of selecting external controls is complex and depends on a range of factors. When multiple historical trials are available, even if a rationale for the choice of historical controls is provided, one may not be able to rule out that the selection of historical controls is also influenced by outcome data from these trials. In particular, if trial results are in the public domain when planning a hybrid trial, the hybrid trial protocol can be written based on this information. Current methodological approaches focus on adjustments to ensure comparability between external data and prospective randomized data. Less attention has been given to aspects such as the information available when controls are selected and ways to measure the potential bias that arises if the choice of historical controls is informed by the outcome of the historical trials. A particular concern is the bias introduced when controls are selected because their outcomes are more negative than expected. We refer to this as outcome‐dependent selection (ODS): the selection or exclusion of historical data is explicitly or implicitly based on their observed outcomes. Once the selected pool of studies is biased, even robust dynamic borrowing methods may propagate at least part of that bias to the final inference, leading to biased hypothesis tests and treatment effect estimates. ODS bias has been identified by both regulators and independent reviewers as a threat to validity; ICH E10 and FDA's 2023 draft guidance on externally controlled trials both stress that external‐control sources must be finalized before any comparative analysis, and Burger et al. labeled such post hoc choices as “retrospective selection bias” [10, 14, 37].

To further illustrate ODS, suppose three historical trials are available with observed control response rates of 0.15, 0.20, and 0.25, and a hybrid RCT is planned. Because these outcomes are already known at the design stage, one might be tempted to exclude a trial based on its outcome to align the historical pool with an anticipated prospective control rate near 0.20. For example, dropping the highest‐rate trial (0.25) reduces apparent heterogeneity but also lowers the pooled historical mean to (0.15+0.20)/2=0.175, which can tilt borrowing toward larger estimated treatment effects. In practice there are often seemingly reasonable grounds to exclude a study, such as the age of data, differences in endpoint definition, or population shifts. However, once these decisions are linked to observed outcomes, they constitute ODS and might introduce bias.

To assess the risk of ODS, we conducted a comprehensive simulation study that quantified the bias introduced when historical trials were selected with knowledge of their outcomes. The simulations investigated the impact of different forms of cherry‐picking: selecting historical controls because their observed outcomes made the experimental treatment appear more favorable. To illustrate the ODS mechanism transparently, we used a simple binary endpoint setting with a one‐sided superiority test comparing experimental and control response rates. We focused on test‐then‐pool and the robust meta‐analytic‐predictive (MAP) prior as two representative dynamic borrowing approaches: the former uses a pre‐test to decide whether to pool the randomized and historical controls in a frequentist framework, whereas the latter uses a Bayesian mixture prior to limit borrowing when the randomized and historical controls are incompatible. We then investigated how the bias varied with study‐level heterogeneity, the size and number of available historical studies, and the mechanism used to select historical controls. Importantly, the hypothetical selection mechanisms we studied are not recommended for practice. They were intended to mimic real‐world scenarios in which sponsors might (deliberately or inadvertently) trim the external‐control pool to obtain higher conditional power before submitting the statistical analysis plan. We showed that both Bayesian and frequentist methods are prone to such selection bias, and the various hypothetical selection mechanisms investigated in the simulation studies illustrate how severe the bias may become.

Two simulation frameworks are commonly used to evaluate historical borrowing methods. In a *conditional* framework, the historical dataset is treated as fixed and only the prospective RCT is evaluated. Thus, operating characteristics (e.g., Type I error rate and power) are interpreted relative to the observed external evidence. In an *unconditional* framework, both historical and prospective data are generated in each replicate, and results are averaged over variability in both sources, reflecting long‐run performance across possible historical evidence pools [38]. The simulation study in this paper adopts the unconditional framework. By contrast, the case study is conditional: a fixed historical pool is used to (i) evaluate design operating characteristics via exact calculations for binary endpoints and (ii) perform posterior analyses given the observed trial data.

The remainder of this paper is organized as follows. Section 2 describes the analysis framework. We introduce both Bayesian and frequentist methods to allow statistical inference while utilizing (some) historical control data. Section 3 describes the simulation setup and reports the operating characteristics of different selection mechanisms from the simulation. We explore a set of selection mechanisms for historical controls to investigate potential biases in an extensive setup for clinical trial simulations. Section 4 illustrates the impact of the selection mechanisms with a case study. Section 5 discusses implications for protocol development and offers recommendations on prespecifying external‐data selection to minimize outcome‐dependent selection bias.

## 2 Methods

We consider hybrid RCTs, based on a prospective trial with 1:1 randomization using a binary endpoint, and a pool of k historical trials for which only the control data are considered. We assume that the historical controls used in the analysis are selected based on specific selection mechanisms. The hybrid RCT then incorporates these historical controls to augment the prospective control arm in the analysis (Figure 1b). As a reference, we compare this design with a stand‐alone RCT consisting of the prospective trial only (Figure 1a). For both types of trials, we consider both frequentist and Bayesian analysis methods (Figure 2). For the stand‐alone RCT, Fisher's exact test is used in the frequentist framework, while in the Bayesian framework, both arms are fit with uniform priors (i.e., a Beta (1,1) prior for a dichotomous outcome). For the hybrid RCT, dynamic borrowing is implemented using a test‐then‐pool approach in the frequentist framework, whereas a robust MAP approach is utilized for the Bayesian analysis. Because hybrid RCTs often randomize more participants to the experimental treatment to maximize potential benefits, we also explore unequal randomization ratios of 2:1 and 3:1 while keeping the total sample size fixed in the prospective part of the hybrid RCT.

**FIGURE 1:**
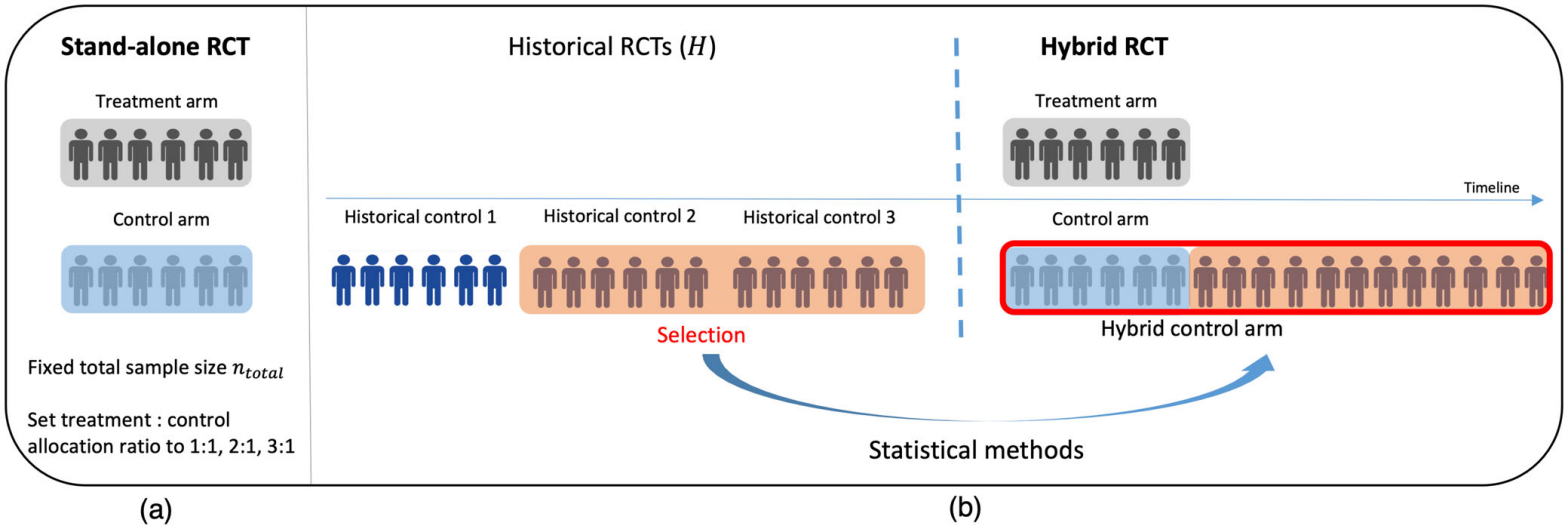
(a) Stand‐alone RCT with prospective treatment arm in gray, prospective control arm in blue. (b) Illustration of the selection of historical trials and the structure of a hybrid RCT. The x‐axis shows a timeline, and the vertical blue dashed line differentiates the historical RCT data and the prospective hybrid RCT. Selected historical trials are shown in orange, the prospective treatment arm in gray, and the prospective control arm in blue. Selected historical controls are combined with the prospective control to form a hybrid control arm (red circle).

**FIGURE 2:**
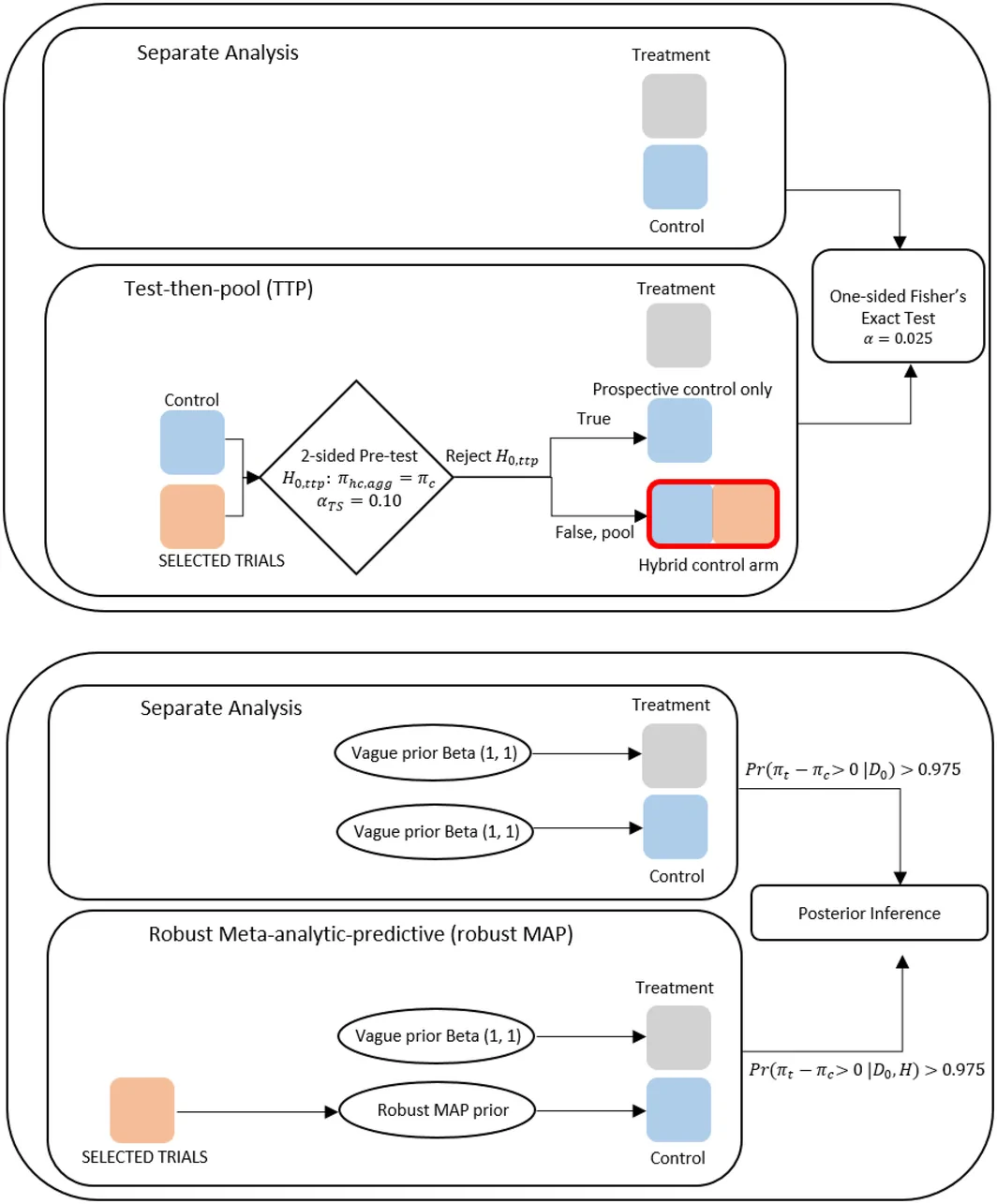
Analysis framework of the simulation study. Top: Frequentist analysis. The top panel shows separate analysis with a one‐sided Fisher's exact test using prospective trial data only (gray and blue). The bottom panel shows test‐then‐pool, which uses a two‐sided pre‐test to assess similarity between the selected historical controls and the prospective control; after the pooling decision, a one‐sided Fisher's exact test is performed for treatment effect assessment. Bottom: Bayesian analysis. The top panel shows separate analysis fitting a vague Beta (1, 1) prior to both arms. The bottom panel shows the robust MAP approach, where the robust MAP prior is obtained from selected historical trials (orange). Circles indicate the prior of the analysis, which is then updated with the prospective treatment and control data (gray and blue) to conduct posterior inference.

The parameter of interest is the treatment effect defined as the risk difference (RD):

$$\mathrm{RD}=\pi_{t}-\pi_{c},$$

where $\pi_{t}$ and $\pi_{c}$ are the true response probabilities for patients eligible for the prospective trial under treatment and control, respectively. External controls (historical trials) may inform the estimation of $\pi_{c}$ through pooling or prior augmentation. We test the hypotheses

$$H_{0}:\mathrm{RD}\leq 0\;\mathrm{vs}.\;H_{1}:\mathrm{RD}>0.$$

While there are several methods available for leveraging information from multiple historical trials, in this study we focus on TTP and robust MAP. The risk difference is estimated as the difference between the mean response rates in the treatment and control arms. For the frequentist analyses, we use sample means from the prospective trial, with the option to pool historical controls when appropriate; for the Bayesian analyses, we use posterior means under either a vague prior or priors derived from historical controls.

### 2.1 Separate Analysis

A stand‐alone RCT implies that only data from the prospective trial itself are used in the analysis. This is denoted by the term separate analysis which uses only prospective trial data and serves as a *no‐borrowing* benchmark. For the frequentist analysis, a one‐sided Fisher's exact test at level α=0.025 is performed. In the corresponding Bayesian analysis we assume for both arms Beta(1,1) priors and declare success when

$$\mathrm{Pr}\;\left(\pi_{t}-\pi_{c}>0\;|\;D_{0}\right)>0.975,$$

where $D_{0}$ denotes the prospective trial data in both arms.

### 2.2 Test‐Then‐Pool

Historical controls from k trials are first selected (Section 3.1), then treated as a single historical control dataset. Consistency between historical and prospective controls is assessed by a two‐sided pre‐test (Fisher's exact test) at level $\alpha_{\mathrm{TS}}$, testing the hypotheses

(1) $$H_{0,\mathrm{TTP}}:\;\pi_{\mathrm{hc},agg}=\pi_{c}\;\mathrm{vs}.\;H_{1,\mathrm{TTP}}:\;\pi_{\mathrm{hc},agg}\neq \pi_{c},$$

where $\pi_{\mathrm{hc},agg}$ and $\pi_{c}$ are the aggregated historical and prospective control response rates, respectively. Failure to reject $H_{0,\mathrm{TTP}}$ indicates that there is no significant difference between the historical and prospective controls. Therefore, the selected historical and prospective control observations are pooled with equal individual weights to form a hybrid control arm for the subsequent treatment‐control comparison. On the other hand, if the difference between historical and prospective controls is significant at level $\alpha_{\mathrm{TS}}$, the analysis proceeds with the prospective control only. In both cases, the subsequent treatment‐control comparison is performed with a one‐sided Fisher's exact test at level α=0.025 using either the pooled controls or the prospective controls only.

### 2.3 Robust Meta‐Analytic‐Predictive Priors

Neuenschwander et al. [25] proposed to summarize historical information in a meta‐analysis framework. A MAP prior is derived from the historical data to inform the prospective trial. This MAP prior has no simple parametric form but can be approximated by fitting a finite mixture of natural conjugate priors [39]. For example, for binary endpoints, the MAP prior derived from binomial controls is well approximated by a mixture of beta distributions. Schmidli et al. [40] further proposed a robust version of the MAP prior that accounts for potential prior‐data conflict. This robustification is achieved by averaging a vague prior and the MAP prior, with a prespecified weight ($w_{R}$) that corresponds to the probability of conflict between the historical controls and the new trial, see Equation (2). $w_{R}=1$ assigns full weight to the vague prior and leads to the separate analysis, while $w_{R}=0$ corresponds to a full MAP prior. The resulting prior is given by

(2) $$\begin{array}{ll} {\hat{p}}_{H,\text{robust}}\left(\pi_{\mathrm{new}}\right) & =w_{R}\overset{\text{vague}\;\text{prior}}{\overbrace{\text{Beta}\left(1,1\right)}}+\left(1-w_{R}\right)\\ & \;\overset{\mathrm{MAP}\;\text{prior}\;\text{from}\;\text{historical}\;\text{trials}}{\overbrace{\sum_{\ell =1}^{K}w_{\ell }\text{Beta}\left(\pi_{\mathrm{new}}|a_{\ell },b_{\ell }\right)}}\;\;\text{with}\sum_{\ell =1}^{K}w_{\ell }=1, \end{array}$$

where ℓ indexes mixture components. Historical controls were selected using the selection mechanisms in Section 3.1 prior to fitting the robust MAP. The subsequent robust MAP analysis borrows from the selected historical trials, with the degree of borrowing governed by between‐trial similarity in the MAP/robust MAP framework. The treatment arm in the prospective trial is informed by a vague Beta (1,1) prior. We declare success if

$$\mathrm{Pr}\;\left(\pi_{t}-\pi_{c}>0\;|\;D_{0},ℋ\right)>0.975,$$

where $\pi_{t}$ and $\pi_{c}$ denote the treatment and control response rates, $D_{0}$ the prospective trial data, and ℋ the historical data used for borrowing. Full posterior updating and computation of posterior superiority probabilities are detailed in Supporting Information S1. Analyses were implemented in R with the RBesT [41] package.

## 3 Simulation Study

The main objective of this simulation study is to evaluate how the operating characteristics of a hybrid clinical trial design are affected by the selection of historical controls. We performed simulations to evaluate marginal operating characteristics averaged over the prior distribution of the control response rates and the sampling distribution of outcomes in the historical trials: Type I error rate, power, bias, root mean square error (RMSE), and effective sample size (ESS) of the selection mechanisms described in Section 3.1. See Table 1 for a summary of the assessed operating characteristics.

**TABLE 1:** Marginal operating characteristics estimated in the simulation study averaged over the priors and the sampling distribution.

Measure	Definition	Estimation	Description
Bias	$E\left[\hat{R}D-\mathrm{RD}\right]$	$\frac{1}{n_{\mathrm{sim}}}\sum_{r=1}^{n_{\mathrm{sim}}}\left(\hat{R}D^{\left(r\right)}-\mathrm{RD}\right)$	Systematic deviation of the estimated treatment effect from the true value.
RMSE	$\sqrt{E\left[{\left(\hat{R}D-\mathrm{RD}\right)}^{2}\right]}$	$\sqrt{\frac{1}{n_{\mathrm{sim}}}\sum_{r=1}^{n_{\mathrm{sim}}}{\left(\hat{R}D^{\left(r\right)}-\mathrm{RD}\right)}^{2}}$	Average magnitude of estimation error, in the same units as RD.
Power	$\mathrm{Pr}\left(\text{reject}\;H_{0}|H_{1}\right)$	Proportion of rejections	Probability of rejecting $H_{0}$ under the specified alternative.
Type I error rate	$\mathrm{Pr}\left(\text{reject}\;H_{0}|\mathrm{RD}=0\right)$	Proportion of false positives	Probability of rejecting $H_{0}$ when treatment has no effect.
ESS	Effective sample size of the prior	Average prior ESS calculated using the expected local‐information‐ratio (ELIR) method [42]	Quantifies historical information contributed by borrowing, expressed as an equivalent number of concurrent randomized control observations. Averaged over replicates; varies with the selection mechanism.

*Note:* The power is evaluated under $H_{1}$ and the Type I error rate is evaluated under $H_{0}$. Bias and RMSE are evaluated under both $H_{0}$ and $H_{1}$, where RD denotes the scenario‐specific marginal true risk difference described in Section 3.

### 3.1 Simulation Setup

#### 3.1.1 Data‐Generating Mechanism

We consider k historical control arms indexed by i=1,…,k. For each historical arm with sample size $n_{i}$, the binary response count $Y_{i}$ follows a binomial distribution $Y_{i}∼\mathrm{Bin}\left(n_{i},\pi_{i}\right)$, where $\pi_{i}\in \left(0,1\right)$ is the event probability simulated from

(3) $$\theta_{i}=\text{logit}\left(\pi_{i}\right)=\beta_{0}+u_{i},\;u_{i}\overset{\mathrm{iid}}{∼}N\left(0,\tau^{2}\right).$$

$\theta_{i}$ is the log‐odds of $\pi_{i}$ and $u_{i}$ is the trial‐specific random effect, which models the between‐trial heterogeneity and has variance $\tau^{2}$. The prospective trial is indexed by i=k+1 and has two arms: a concurrent control arm with event probability $\pi_{c}$ and a treatment arm with event probability $\pi_{t}$. Their log‐odds are $\theta_{c}=\text{logit}\left(\pi_{c}\right)=\beta_{0}+u_{k+1}$ and $\theta_{t}=\text{logit}\left(\pi_{t}\right)=\beta_{0}+\beta_{1}+u_{k+1}$, where $u_{k+1}∼N\left(0,\tau^{2}\right)$. Thus the historical controls and the concurrent control arm share the same random‐effects distribution $\theta_{1},\ldots ,\theta_{k},\theta_{c}\overset{\mathrm{iid}}{˜}N\left(\beta_{0},\tau^{2}\right)$.

The fixed effects $\left(\beta_{0},\beta_{1}\right)$ were chosen on the logit scale. Under $H_{1}$, $\beta_{0}=\text{logit}\left(0.20\right)=\log\;\left(\frac{0.20}{0.80}\right)$, $\beta_{1}=\text{logit}\left(0.40\right)-\text{logit}\left(0.20\right)$, so that at the reference level $u_{k+1}=0$, $\pi_{c}=0.20$ and $\pi_{t}=0.40$, corresponding to a risk difference of 0.20. Under $H_{0}$, $\beta_{1}=0$. Thus, the treatment effect on the log‐odds scale $\beta_{1}$ was fixed, and the corresponding risk difference varied with $u_{k+1}$. For each value of τ, bias and RMSE were calculated relative to the marginal true risk difference obtained by averaging $\pi_{t}-\pi_{c}$ over the distribution of $u_{k+1}$.

For ease of presentation, the quantities labeled $E\left(\pi_{c}\right)$ and $E\left(\pi_{\mathrm{hc}}\right)$ in the scenario descriptions, tables, and figures denote the reference response rates conditional on the corresponding trial‐specific random effect being zero, that is, $E\left(\pi_{c}|u_{k+1}=0\right)$ and $E\left(\pi_{\mathrm{hc},i}|u_{i}=0\right)$.

#### 3.1.2 Trial Design Choices and Scenario Assumptions

Following simulation reporting guidelines [43, 44, 45], we distinguish trial design choices (D) from scenario assumptions (A). In all simulations, TTP was implemented with a two‐sided Fisher's exact pre‐test at level $\alpha_{\mathrm{TS}}=0.10$ for the pooling decision, followed by a one‐sided Fisher's exact test to assess the treatment effect. Robust MAP was implemented with $w_{R}=0.1$. Table 2 summarizes the design options that were varied in the simulation study, such as the prospective trial sample size $n_{\text{total}}$ and randomization ratios. In addition, the considered assumptions (between‐trial heterogeneity τ, number of historical trials k, sample size of historical controls per trial $n_{\mathrm{hc}}$, and the true prospective control response rate $\pi_{c}$) are listed. In particular, we consider the main simulation Scenarios 1a and 1b, and two extensions, Scenarios 2 and 3, described below.

- Scenario 1: All historical controls are simulated with response rate $\pi_{\mathrm{hc}}$ according to Equation (3) where $E\left(\pi_{\mathrm{hc}}\right)=0.20$.
  ∘ Scenario 1a *Exchangeable*: When the prospective control response rate also satisfies $E\left(\pi_{c}\right)=0.20$, the historical and prospective control response rates ($\pi_{\mathrm{hc}},\pi_{c}$) are *exchangeable*; their study‐specific logits are i.i.d. $N\left(\beta_{0},\tau^{2}\right)$. Thus, they are drawn from the same distribution (differences across historical and prospective controls arise only from the study‐specific random effects $u_{i}$), and on average (averaged over the random effects) the response rates in the historical and concurrent controls are the same.
  ∘ Scenario 1b *Prospective‐historical distributional shift*: Eπc=0.20 such that the prospective control arm shows a prior‐data conflict on average.
- Scenario 2 *Time trend*: We impose a linear time effect on the historical controls with $\beta_{2}=-0.05$ on the logit scale (only for the case of k=8 historical control trials each with a sample size of $n_{\mathrm{hc}}=30$). The drift is introduced by adding $\beta_{2}\left(k-i+1\right)$  Equation (3) and is anchored at zero for the prospective trial (i=k+1), so historical controls drift from the prospective baseline. Consequently, historical response rates rise from ≈0.14 (earliest) toward ≈0.20 (most recent), while the prospective control response rate matches the scenario 1 (≈0.20); thus, any differences arise solely from the trend in historical studies.
- Scenario 3 *Large prospective trial*: In addition to the scenario 1 ($n_{\text{total}}=60,180$ with RD=0.20), we generated larger trials ($n_{\text{total}}=500$) with smaller effects RD=(0.0635,0.1152), while fixing the historical trials with sample size per arm of $n_{\mathrm{hc}}=\left(30,90\right)$. These values were calibrated such that the no‐borrowing design achieved approximately the same power (≈39% and 84%) as the smaller trials, thereby isolating the gain from borrowing.

**TABLE 2:** Factors varied in the simulation, classified as Design (D) or Assumption (A), with investigated values and description.

Factor	Type	Investigated values	Description
τ	A	0.10, 0.30, 0.50	Between‐trial heterogeneity; values motivated by Neuenschwander et al. [25].
k	A	4, 8	Number of historical trials (control arm only).
$n_{\mathrm{hc}}$	A	30, 90	Historical control sample size per trial.
$n_{\text{total}}$	D	60, 180	Prospective trial total sample size.
Randomization ratio	D	1:1, 2:1, 3:1	Allocation ratio of prospective trial (treatment: control), with fixed $n_{\text{total}}$.
$\pi_{c}$	A	0.15–0.75 (step 0.05)	True prospective control response rate.

*Note:* Type: D = Design, a feature of the prospective trial set by the investigators; A = Assumption, a feature that lies beyond the direct influence of the investigators.

Table 3 summarizes the simulation scenarios. For each combination of scenario and design, 10000 simulation runs were performed. All scenarios were simulated using the same random number seed to reduce the Monte Carlo error between scenario comparisons. For each simulation run, we simulated both historical and prospective data, to obtain *marginal* operating characteristics, that is, operating characteristics that are averaged over the variability in both the response rates and the sampling distributions of historical and prospective data, rather than conditional on a fixed set of historical controls [38].

**TABLE 3:** Simulation scenarios for prospective trial.

Scenario	Name	Description
1a	Exchangeable	No distributional shift, $E\left(\pi_{\mathrm{hc}}\right)=E\left(\pi_{c}\right)=0.20$, historical and prospective control response rates $\pi_{\mathrm{hc}},\pi_{c}$ are exchangeable.
1b	Prospective‐historical distributional shift	$E\left(\pi_{\mathrm{hc}}\right)=0.20$, prospective $E\left(\pi_{c}\right)$ varied from 0.15 to 0.75 (step 0.05). Represents prior‐data conflict.
2	Time trend	Linear logit drift ($\beta_{2}=-0.05$) across historical trials for $k=8,\;n_{\mathrm{hc}}=30$; prospective trial fixed at $E\left(\pi_{c}\right)=0.20$, earliest historical trial $E\left(\pi_{\mathrm{hc}}\right)\approx 0.14$.
3	Large prospective trial	Larger prospective trial with total sample size ($n_{\text{total}}$) = 500 with smaller effects RD=(0.0635,0.1152), calibrated so that the no‐borrowing design achieves ≈39% and 84% power, respectively.

*Note:* The scenario numbers are used for reference in the Results section. RD denotes risk difference in prospective trial used for calibration. $\pi_{\mathrm{hc}}$: historical control trials response rate; $\pi_{c}$: prospective trial control response rate.

#### 3.1.3 Considered Selection Mechanisms

To investigate how the selection of historical controls (and discarding some of them) may impact important operating characteristics, we explore a range of different hypothetical selection mechanisms summarized in Table 4. These selection mechanisms are not proposed for practical use, but were chosen as illustrative examples of selection mechanisms that might be applied when designing a hybrid RCT. The selection mechanisms range from approaches unrelated to the observed effect, such as selecting all k available historical controls (“Full selection”) or randomly selecting some of the control arms (“Random selection”), to mechanisms that use information on the observed response rates. Among the latter, some are more extreme than others. For example, “Drop‐the‐best” simply drops a single historical control, that is, the one with the largest (“best”) observed response rate. Another selection mechanism drops all historical controls with a too positive outcome, that is, where the observed response rate is above a certain threshold (“threshold selection”).

**TABLE 4:** Description of the hypothetical selection mechanisms when k historical trials are available.

Selection mechanism	Description	ODS^a^	Number of selected trials^b^
Full selection	Utilize all k available historical trials as external controls (k=4,8).	N	k
Random selection	Randomly drop a historical control.	N	k−1
Drop‐the‐best	Drop the historical control with the highest response rate.	Y	k−1
Threshold selection	Select all historical controls with response rates ≤ a prespecified threshold (e.g., 20%; see Figure 3b).	Y	Random
Optimal power selection	Choose the subset that maximizes conditional power, assuming known current‐trial parameters.	Y	Random
Separate analysis	Discard all historical controls (no borrowing).	—	—

^a^ ODS: Outcome‐dependent selection that indicates whether the observed response rates are used. Y = Yes; *N* = No.

^b^ “Random” indicates that the number of selected historical trials varies by selection mechanism (e.g., threshold or optimal power selection).

Figure 3 illustrates the considered selection mechanisms based on an exemplary set of k=8 historical controls. As defined in the introduction, ODS refers to the selection or exclusion of historical controls based, explicitly or implicitly, on their observed outcomes. In Table 4, Drop‐the‐best, threshold selection and optimal power selection are classified as ODS mechanisms.

**FIGURE 3:**
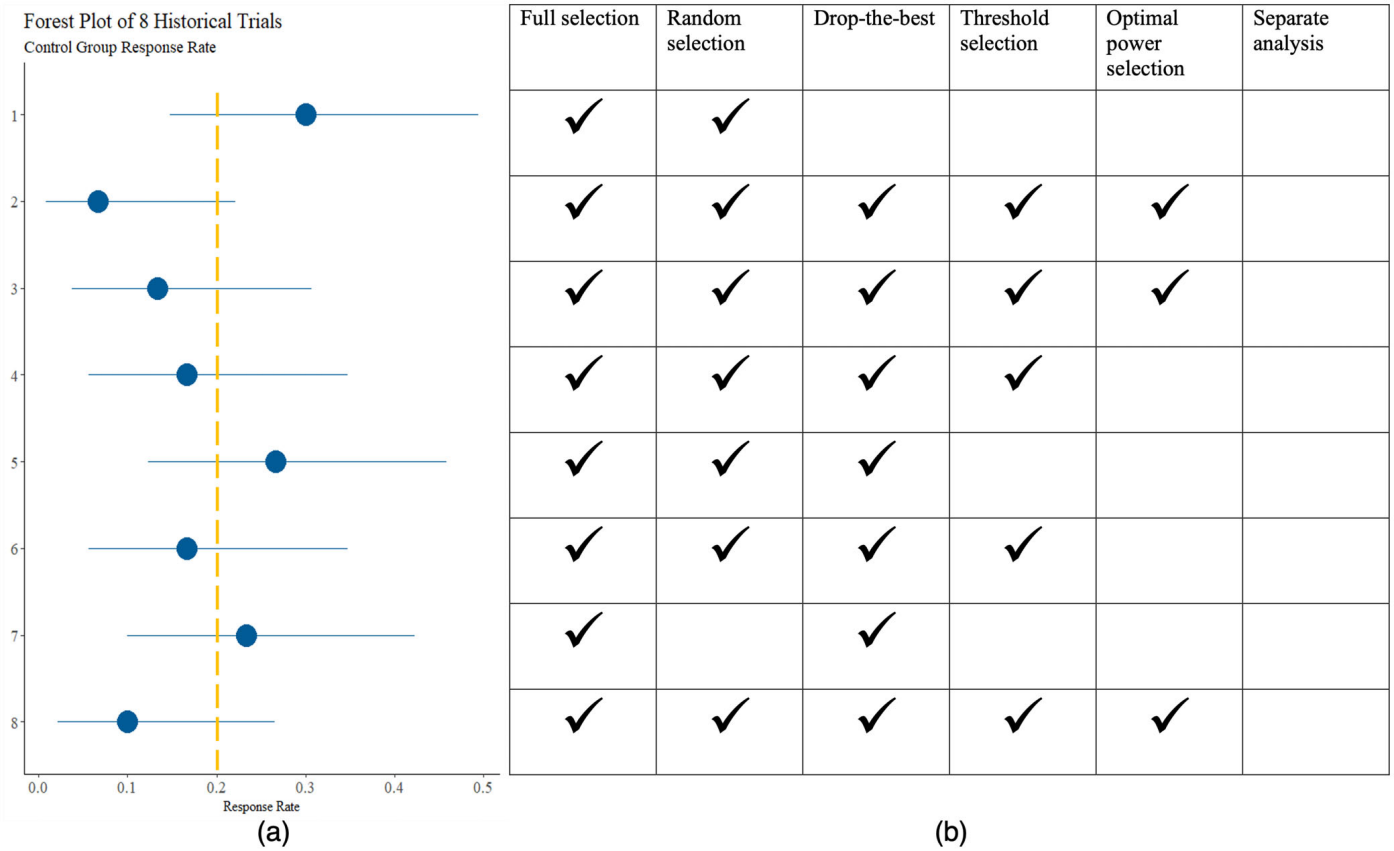
Illustration of different hypothetical selection mechanisms implemented in this study with k=8 historical trials. (a) Forest plot shows control group response rates with 95% confidence intervals. The yellow line indicates the threshold ($\pi_{\mathrm{hc}}=0.20$). (b) Table that indicates which historical trials are selected with indicators (✓) under each selection mechanism. For a description of the selection mechanisms see Table 4. The historical trials are numbered in chronological order.

Under the optimal power selection mechanism, the subset of historical controls that leads to the maximum conditional power to reject the null hypothesis in the prospective trial is selected. Here we assume that the prospective trial has not been performed yet but the distributions from which observations of the prospective trial will be sampled are known. The conditional power is defined as the conditional probability, given the data of the selected historical controls, that the prespecified Bayesian success criterion, $\mathrm{Pr}\left(\pi_{t}-\pi_{c}>0|D_{0},ℋ\right)>0.975$, will be met after the prospective trial data have been observed. Here it is assumed that the prospective trial data are sampled with known response rates $\left(\pi_{t}^{⋆},\pi_{c}^{⋆}\right)$ and the trial has sample sizes $\left(n_{t},n_{c}\right)$. Full technical details are provided in appendix for the optimal power selection mechanism. Under the threshold and optimal power selection mechanisms, there might be the situation that none or only one historical trial is selected (see Supporting Information S2).

### 3.2 Results

#### 3.2.1 Scenario 1a: Historical Control Response Rates and Prospective Control Response Rate Are *Exchangeable*

In the frequentist setting, TTP with full or random selection maintains the Type I error rate below 0.025 only when between‐trial heterogeneity is low. ODS mechanisms inflate the Type I error rate, especially with larger treatment:control randomization ratios (Supplementary Figure S1). In the Bayesian analysis using a robust MAP prior, full and random selection keep the Type I error rate close to the nominal 0.025 under low to moderate heterogeneity (Supplementary Figure S1) and when historical studies are small ($n_{\mathrm{hc}}$ = 30). Under high heterogeneity, borrowing with these selection mechanisms exceeds the nominal Type I error level. With robust MAP, Type I error inflation is more severe when borrowing from smaller historical studies ($n_{\mathrm{hc}}=30$), but this does not hold for TTP (Supplementary Figure S2). Across both analysis frameworks, ODS mechanisms inflate the Type I error rate, with the greatest inflation at high heterogeneity. When more historical trials are available (k=8), ODS mechanisms that select a random number of studies (e.g., threshold selection and optimal power selection) have more degrees of freedom and tend to cause more severe inflation. Type I error inflation is much more severe with ODS mechanisms when the historical study pool has large k and small $n_{\mathrm{hc}}$ than when it has small k and large $n_{\mathrm{hc}}$, regardless of the level of between‐trial heterogeneity (Supplementary Figure S2). This warrants particular care when several small historical trials are available.

ODS mechanisms lead to higher power than full and random selection when the prospective trial is small ($n_{\mathrm{hc}}=30,n_{\text{total}}=60$, Supplementary Figure S4). ODS mechanisms provide the greatest power advantage when between‐trial heterogeneity is large (Supplementary Figure S3). The largest relative power gain from borrowing occurs with a 3:1 randomization ratio, although this ratio has lower absolute power than 1:1, regardless of the selection mechanisms (Supplementary Figure S3). More patients are allocated to the experimental treatment arm while achieving similar power when we borrow the data. When between‐trial heterogeneity is small (τ=0.1) and the historical data set is large ($n_{\mathrm{hc}}=90$), all historical controls are highly similar to the prospective control. In this situation, ODS mechanisms offer no power gain over simply using all or randomly chosen historical controls, because nearly every trial in the pool contributes useful information.

With TTP, ODS mechanisms introduce positive bias in the treatment effect estimates. This bias is larger with threshold selection and optimal power selection and when the sample size in the prospective trial is small ($n_{\text{total}}=60$). With this sample size, the biases for these two selections are around 2–3 percentage points. In the Bayesian framework, if a uniform prior is applied in both arms, even in the separate analysis a negative bias in the treatment effect estimate, under $H_{1}$ (Supplementary Figure S5) is introduced. This is a property of the Bayesian analysis when the sample size is small. The bias becomes more pronounced if unequal randomization is applied and more patients are allocated to the treatment arm. ODS mechanisms introduce a positive bias resulting in an overestimation of the treatment effect (Supplementary Figures S5 and S6). A higher number of historical studies (k=8) increases the bias introduced by ODS when the robust MAP is used (Supplementary Figure S6). Similar findings are observed under $H_{0}$ (Supplementary Figures S7 and S8).

Borrowing historical controls reduces RMSE whenever heterogeneity is low (τ=0.1), regardless of the selection mechanisms (Supplementary Figure S9). At moderate heterogeneity (τ=0.3), an RMSE reduction is seen only for $n_{\mathrm{hc}}=30$. Threshold selection and optimal power selection yield higher RMSE compared to other selection mechanisms across all levels of between‐trial heterogeneity (τ), the number of historical studies (k), or the sample size within each historical study ($n_{\mathrm{hc}}$) (Supplementary Figures S9 and S10). Similar findings are observed under $H_{0}$ (Supplementary Figures S11 and S12). RMSE increases with higher randomization ratio (keeping $n_{\text{total}}$ fixed) as unequal treatment:control allocation increases the variance of the RD estimate. Borrowing historical controls can reduce the impact of randomization ratio when τ=0.1,0.3.

For the robust MAP, we report the ESS, representing the amount of information that is contributed by the selected historical controls in the treatment‐control comparison, for the different selection mechanisms in the Supplementary Tables S1–S4. With increasing between‐trial heterogeneity (τ), the ESS decreases across all settings of $k,n_{\mathrm{hc}}$ and selection mechanisms. This ESS reduction with increasing τ is smaller with more flexible ODS mechanisms like threshold selection and optimal power selection, because these selection mechanisms keep a more homogeneous subset of studies, at the cost of selection bias. For larger τ or k, ODS mechanisms can lead to higher ESS than full or random selection because greater heterogeneity or a larger number of historical studies that can be selected enables more extreme selections. For optimal power selection, the prospective trial's treatment:control randomization ratio affects the sample size $\left(n_{t},n_{c}\right)$ and thus influences the selection. However, changing the treatment:control ratio has only minor, non‐systematic effects on ESS (Supplementary Tables S3 and S4). The ESS with increasing randomization ratio remains stable or decreases slightly. Both drop‐the‐best and random selection retain k−1 studies, but drop‐the‐best yields larger ESS because removing the highest response historical trial reduces heterogeneity, at the cost of potential bias.

#### 3.2.2 Scenario 1b: Distributional Shift in the Prospective Control Population Resulting in Prior‐Data Conflict

Figure 4 illustrates the impact of *prospective–historical distributional shift*, which is the case when historical data differ from the prospective control response rates (scenario 1b). Borrowing will only be appropriate when the prospective control response rates are similar to historical control response rates (around 0.20, vertical line). In this case, full and random selection show no bias, lower RMSE, higher power, and well‐controlled Type I error rate compared to the separate analysis. ODS mechanisms (dashed lines) achieve higher power but at the cost of greater bias, higher RMSE (Figure 4d–f), and inflated Type I error rate (Figure 4a). When the prospective control response rate is larger than expected, the operating metrics showed a dynamic pattern: an initial increase followed by a decline. Inflated Type I error rates are expected whenever information is borrowed, consistent with the findings of Kopp‐Schneider [46]. In this setting, the performance curves for ODS mechanisms eventually intersect those of full and random selection because ODS mechanisms preferentially draw on historical studies with lower response rates. As the prospective control response rate increases, the dynamic borrowing methods then borrow less information with ODS mechanisms as the conflict grows and revert to no borrowing earlier than full and random selection. These findings hold across all simulation scenarios we examined, regardless of the values of $\tau ,k,n_{\mathrm{hc}}$, although the magnitude of the effects varies.

**FIGURE 4:**
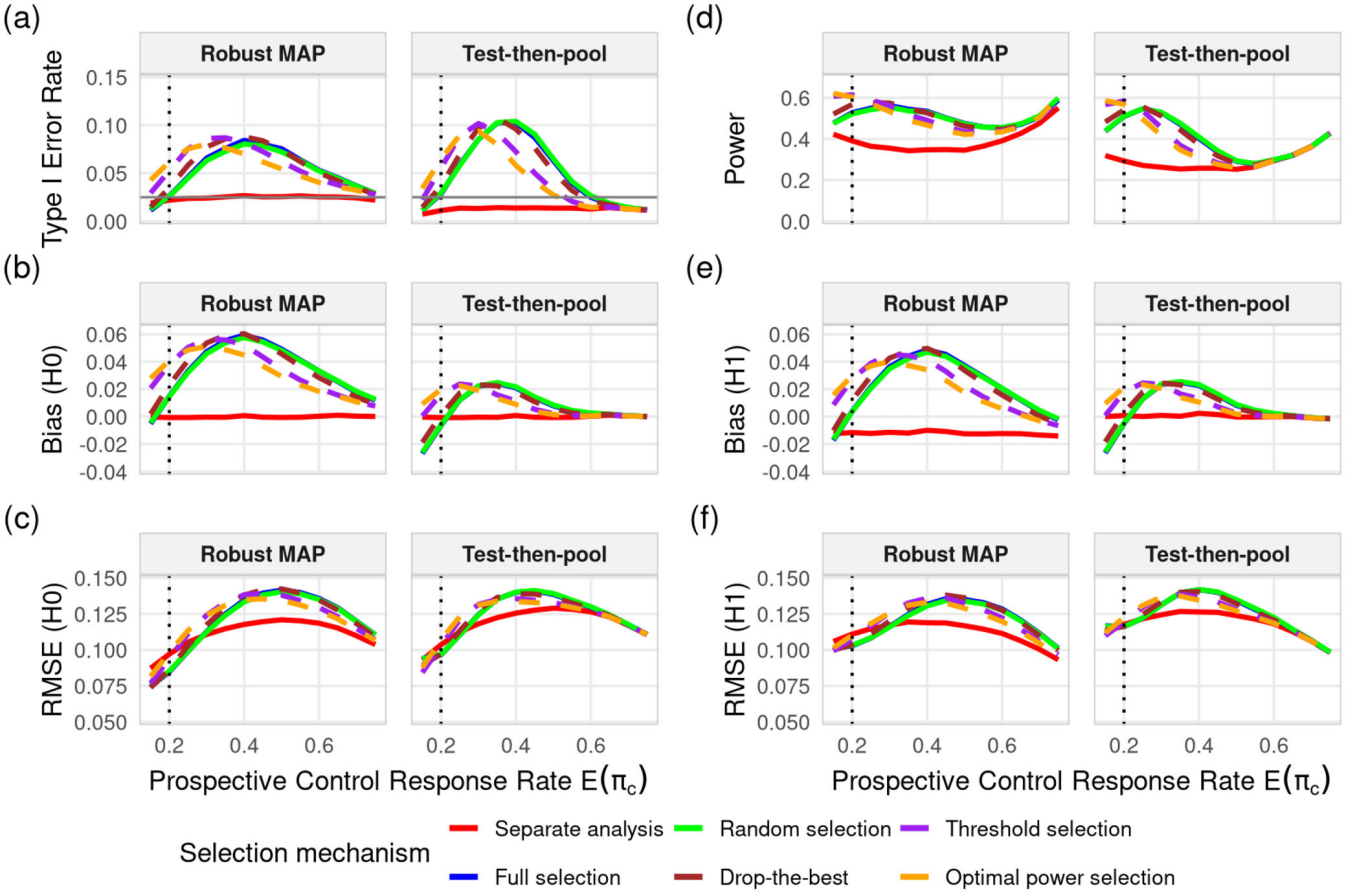
Marginal operating characteristics of selection mechanisms with an initial set of k=8 historical control trials each with a sample size of $n_{\mathrm{hc}}=30$ and assuming a moderate between‐trial heterogeneity of τ=0.30, prospective trial total sample size $n_{\text{total}}=60$ (for more details on the simulation setup see Section 3). (a)–(c) $H_{0}$: Type I error rate, bias, RMSE. (d)–(f) $H_{1}$: Power, bias, RMSE. x‐axis: Prospective control response rate; the vertical line at x=0.20 indicates exchangeability, where the maximum amount of borrowing is appropriate; dashed lines denote outcome‐dependent selections.

#### 3.2.3 Scenarios 2 and 3: Time Trend and Large Prospective Trial Scenarios

A time trend was applied by adding a linear drift on the logit scale $\beta_{2}=-0.05$ to all historical trials (Scenario 2). The resulting mismatch of historical and prospective controls increased bias, root mean‐squared error, and the one‐sided Type I error rate for every selection mechanism (Table 5); even full and random selection, which do not favor extreme studies, now lead to an inflation of the nominal Type I error rate. The ODS mechanisms amplify the problem: drop‐the‐best, threshold, and optimal power selection show larger increases in bias and Type I error rate while providing only modest power gains. When temporal drift is present, borrowing therefore requires a careful assessment of comparability and strong justification.

**TABLE 5:** Marginal operating characteristics (%) with an initial set of k=8 historical control trials each with a sample size of $n_{\mathrm{hc}}=30$ and assuming a moderate between‐trial heterogeneity of τ=0.30 (for more details on the simulation setup see Section 3).

	Exchangeable^a^				Time trend^b^				Large prospective trial^c^			
Selectionmechanism	T1E	Power	Bias	RMSE	T1E	Power	Bias	RMSE	T1E	Power	Bias	RMSE
Separate analysis	2.20	39.05	−1.17	11.09	2.20	39.05	−1.17	11.09	2.57	38.87	−0.04	3.85
Full selection	2.63	52.75	0.38	10.28	3.95	58.32	1.85	10.52	2.83	42.91	0.18	3.90
Random selection	2.60	52.21	0.37	10.31	3.95	57.70	1.78	10.52	2.79	42.62	0.17	3.89
Drop‐the‐best	3.48	56.80	1.21	10.36	5.27	61.92	2.62	10.73	3.28	45.98	0.41	3.92
Threshold selection	5.26	61.43	2.73	10.76	6.54	63.96	3.38	11.01	4.29	49.38	0.80	3.95
Optimal power selection	6.18	60.33	2.98	11.02	7.33	60.67	3.47	11.34	4.35	49.03	0.79	3.95

*Note:* RD: treatment effect measure, risk difference between the treatment and control arm. All values are **percentages**. T1E: Type I error rate at nominal one‐sided level 2.5%. Bias: percentage‐point deviation of posterior mean RD from the truth, under $H_{1}$, calculated by $\frac{1}{n_{\mathrm{sim}}}\sum_{r=1}^{n_{\mathrm{sim}}}\left(\hat{R}D^{\left(r\right)}-\mathrm{RD}\right)\times 100$. RMSE: root mean square error, under $H_{1}$, calculated by $\sqrt{\frac{1}{n_{\mathrm{sim}}}\sum_{r=1}^{n_{\mathrm{sim}}}{\left(\hat{R}D^{\left(r\right)}-\mathrm{RD}\right)}^{2}}\times 100$.

^a^ Exchangeable: reference risk difference RD=0.20 at $u_{k+1}=0$.

^b^ Time trend: linear drift in the historical data.

^c^ Large prospective trial: prospective trial with RD=0.0635 at $u_{k+1}=0$ with n=250 per arm.

In the large prospective trial scenario (Scenario 3) we set the reference risk difference at $u_{k+1}=0$ to 0.0635 and increased each trial arm to 250 participants. Under this design, bias and RMSE decrease for all methods as the prospective trial data dominate the posterior. However, the three ODS mechanisms still show larger biases and Type I error rate inflation, although both distortions are smaller than in Scenario 1. Power differences are also smaller, because the larger prospective control arm leaves comparatively little room for historical data augmentation.

Sensitivity analyses using a half‐normal prior with scale 0.5 for τ provided similar results (data not shown).

## 4 Case Study

We illustrate the impact of selection mechanisms with a case study based on the ankylosing spondylitis (AS) trial data originally reported by Baeten et al. [47]. The original trial was designed as a two‐arm trial testing secukinumab against placebo with a total sample size of 30 and a 4:1 randomization ratio. The primary efficacy endpoint was the Assessment of SpondyloArthritis international Society criteria for 20% improvement (ASAS20) at week 6. This design provided only a very small concurrent control group, and only 3 of the 6 placebo patients reached the week‐6 assessment. Given the small sample size and highly unbalanced allocation, inference based on the concurrent trial alone is highly uncertain. Placebo data from eight previous ankylosing spondylitis trials were included to augment the control arm [48]. This trial assumed true ASAS20 response rates of 25% on placebo and 60% on secukinumab, and these values were used for trial design evaluation ($\pi_{\text{placebo}}=0.25,\pi_{\text{secukinumab}}=0.60,\mathrm{RD}=0.35$ for the power evaluation and selection of the optimal power subset). In this case study, treatment effect is summarized by the posterior mean difference in response rates between secukinumab and placebo. As the original trial borrowed historical placebo data via a MAP prior, our case study assessment focuses on the Bayesian framework, using the robust MAP with a decision threshold of 0.975 for the one‐sided superiority decision.

### 4.1 Design of the Hybrid RCT and Conditional Operating Characteristics

Supplementary Figure S15 lists the historical controls that are selected in this case study with the considered selection mechanisms. Robust MAP priors derived from these selections were incorporated into the analysis as described in Section 2. At the planning stage of the prospective trial, we can evaluate the trial design by calculating the conditional operating characteristics given the actual historical control dataset used and assuming a range of plausible response rates for the prospective part of the hybrid RCT. The trial designs were evaluated in terms of the conditional Type I error rate and conditional power (Figure 5). Conditional Type I error rates are calculated under the null hypothesis of $\pi_{\text{secukinumab}}=\pi_{\text{placebo}}$. Conditional power evaluation is based on $\pi_{\text{secukinumab}}=\pi_{\text{placebo}}+0.35$. Conditional Type I error rates and conditional power were calculated exactly over a range of $\pi_{c}\in \left[0.01,0.60\right]$. To show how results change with a larger prospective sample, we repeated the evaluation for total sample sizes $n_{\text{total}}=60,300,3000$ with the same randomization ratio 4:1. Full results of these trial design evaluations are provided in Supplementary Figure S16.

**FIGURE 5:**
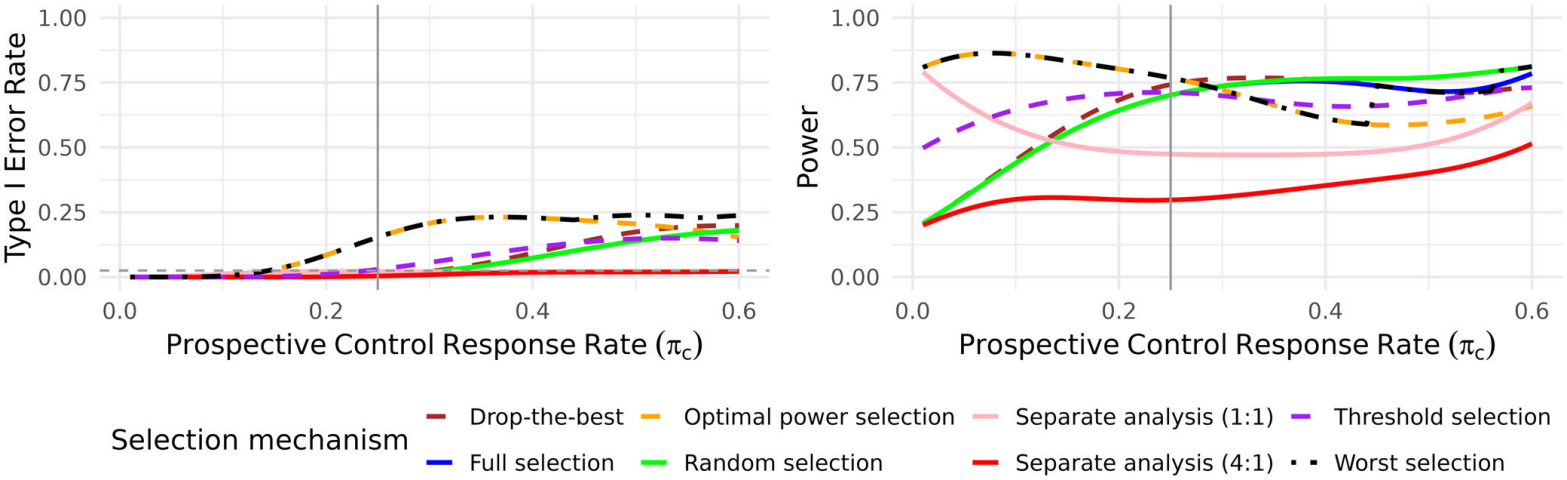
Conditional operating characteristics of different selection mechanisms, based on a case study AS trial, at the trial design stage. Left: Conditional Type I error rates, gray dashed line indicates the nominal Type I error rate 0.025. Right: Conditional power. The vertical line at x=0.25 indicates exchangeability, where the prospective trial control response rate is similar to the mean response rate of the MAP prior derived from all historical studies. *x*‐axis: Prospective control response rate; The black dashed line indicates selection that maximizes the conditional Type I error rates, which is the possible worst selection in this case study.

Threshold selection with threshold ≤ 25% and optimal power selection inflated the conditional Type I error rate even when the prospective control response matched the historical mean. The conditional Type I error rates decreased in cases with a larger sample size in the prospective trial, but outcome‐dependent selections continued to show Type I error rate inflation. In contrast, full selection and random selection kept the Type I error rates below the nominal level (α=0.025). Gains in power from the ODS mechanisms were modest (1–6 percentage points) compared with full and random selection when the control rate matched expectations and disappeared once the prospective control conflicted with the historical data. For $n_{\text{total}}\in \left\{300,3000\right\}$, we report only conditional Type I error rates, because conditional power is essentially one for all methods under the alternative $\pi_{\text{secukinumab}}=\pi_{\text{placebo}}+0.35$.

In addition to conditional Type I error rate and conditional power, we also evaluated the probability of success (PoS, also known as assurance in Bayesian design literature) at the design stage. PoS averages conditional power over the joint prior distributions of treatment and control response rates, thereby reflecting the unconditional chance that the trial will declare success given the current knowledge. Table 6 shows that PoS values differ across selection mechanisms: ODS mechanisms such as threshold and optimal power selection yield higher PoS (0.485 and 0.619, respectively) compared with full and random selection (0.436 and 0.432). Separate analysis results in a substantially lower PoS (0.189), indicating the additional assurance that can be obtained when borrowing external control data. Further details for the calculation of the conditional Type I error rate, conditional power and PoS are available in Supporting Information S4.

**TABLE 6:** Results of the Bayesian robust MAP prior analysis when using different subsets of the available historical control data depending on the selection mechanism illustrated for the case study ankylosing spondylitis (AS) trial data.

Selection mechanism	PoS^a^	Estimate	95% CI	$\mathrm{Pr}\left(\pi_{t}-\pi_{c}>0|D_{0},H\right)$	Number of selected trials
Full selection	0.436	0.335	(0.090, 0.561)	0.994	8
Random selection	0.432	0.332	(0.076, 0.566)	0.993	7
Drop‐the‐best	0.455	0.348	(0.110, 0.566)	0.996	7
Threshold selection	0.485	0.387	(0.123, 0.611)	0.995	3
Optimal power selection	0.619	0.444	(0.223, 0.640)	0.998	1
Separate analysis	0.189	0.327	(−0.047, 0.624)	0.959	0

*Note:* More details on which historical control trials have been selected for which selection mechanism are given in Supplementary Figure S15 in the online supplement. Estimate: Posterior treatment effect estimate (difference of posterior means between the two arms). 95% CI: 95% credible interval. $\mathrm{Pr}\left(\pi_{t}-\pi_{c}>0|D_{0},H\right)$: Posterior probability of treatment benefit, the trial is considered successful if this probability exceeds the threshold of 0.975.

^a^ PoS: Probability of success at the design stage.

### 4.2 Results of the Hybrid RCT

The results in Table 6 are based on an intention‐to‐treat analysis using the Bayesian robust MAP prior approach. All randomized patients were included, and patients without week‐6 ASAS20 outcome were classified as nonresponders. Fourteen of 24 patients responded in the secukinumab arm and 1 of 6 responded in the placebo arm. The separate analysis used independent uniform Beta (1, 1) priors for both arms. The separate analysis failed to demonstrate superiority of the secukinumab arm over the placebo arm, with a posterior probability of treatment benefit of 0.959, below the prespecified decision threshold of 0.975. The estimated treatment effect was 0.327 with a 95% credible interval of (−0.047, 0.624). Similarly, the frequentist separate analysis failed to yield statistical significance using a one‐sided Fisher's exact test at level α=0.025 (see Supplementary Table S6).

When using all available historical controls (“Full selection”), one can demonstrate superiority of the secukinumab arm over the placebo arm as the posterior probability of a positive treatment effect was 0.994. Thus, conditional on the observed trial data and the robust MAP prior constructed from all available historical controls, there was a 99.4% posterior probability of a positive treatment effect, which exceeded the prespecified decision threshold of 0.975. The treatment effect estimate of 0.335 was quite similar to the separate analysis, but by using historical control data the width of the credible interval became narrower, with a 95% credible interval of (0.090, 0.561). A similar effect was observed under random selection. The ODS mechanisms still resulted in much narrower credible intervals despite leaving out some of the historical trials. However, the treatment effect estimates are much higher by leaving out historical controls where the observed effect was “too” good. The “optimal power selection” produced a treatment effect estimate approximately 11 percentage points higher than those obtained with random or full selection. For more details, see Table 6.

However, these apparent gains for the ODS mechanisms in terms of higher posterior probabilities of a positive treatment effect come with the previously noted inflation in the conditional Type I error rate and thus emphasize the trade‐off between efficiency and Type I error rate control illustrated in our simulation study. Similar results are obtained with a frequentist approach when using the TTP (Supplementary Table S6).

## 5 Discussion

Our simulations showed that outcome‐dependent selection of historical controls can bias treatment‐effect estimates and inflate Type I error rates, particularly when there is greater selection flexibility in the candidate pool.

### 5.1 Practical Recommendations for Selecting Historical Controls

Table 7 summarizes practical recommendations informed by our simulation findings and by principles from current regulatory guidance and evidence‐synthesis practice. The recommendations cover different aspects of the construction and evaluation of the historical controls in hybrid RCTs.

**TABLE 7:** Recommendations for the transparent selection of historical controls in hybrid trials and for assessing the consequences of that selection, informed by the findings of our simulation study and by relevant regulatory guidance and literature.

Recommendation	Details	Relevance for avoiding bias	References
1. Specification of the estimand and study‐selection criteria using design‐stage information	Elements to be prespecified independently of historical study data include features related to the estimand, such as the target population, endpoint, summary measure, and control and experimental treatment regimens, as well as details and criteria related to the process for selecting the historical studies, such as search sources, the time range from which studies are selected, and other study‐level screening criteria. These specifications may be based on design‐stage information to align the prospective trial design with the historical studies that may be used in the analysis.	These features determine which studies are selected from the search sources and enter the initial candidate pool. If the choice of these selection criteria is informed by study data, it may favor control groups with outcomes that make the subsequent treatment comparison more favorable, thereby introducing ODS. Even when selection is not based on study outcomes, selection based on design‐stage characteristics can introduce bias if these characteristics are associated with the control outcome. Such bias may be mitigated by covariate adjustment if the relevant characteristics are measured and can be accounted for in the adjustment (Recommendation 4).	[10, 14, 17, 49, 50]
2. Identification and disclosure of the candidate pool of historical studies	The prespecified search sources are searched and potentially relevant studies are identified using the broad screening criteria in Recommendation 1. The search strategy and dates, all identified studies, including those not taken forward for detailed assessment, and relevant limitations in study identification or data access are documented.	In our simulations, a larger k created more possible subsets, whereas a smaller $n_{\mathrm{hc}}$ produced more variable and potentially extreme observed response rates. These features made ODS more consequential and showed the importance of prespecification and disclosure of the complete candidate pool.	[14, 16, 17, 51, 52, 53]
3. Study‐level comparability assessment and refinement of the candidate pool	Each candidate study retained after broad screening is assessed against the prespecified eligibility criteria (Recommendation 1) using relevant study‐level information from the completed study, including study design and conduct, calendar period, endpoint definition, aggregate baseline characteristics, follow‐up completeness, missingness summaries, and other data‐quality features. Where feasible, the assessment is conducted without access to observed historical control outcomes, observed values of correlated endpoints, or other outcome‐related information.	Applying the eligibility criteria may require subjective clinical or methodological judgment and lead to ODS if inclusion decisions are influenced by observed outcomes. Even when applied independently of outcomes, study‐level selection may still induce nonexchangeability if it alters characteristics prognostic for the control outcome relative to the prospective trial population.	[5, 10, 14, 15, 17]
4. Patient‐level comparability assessment and adjustment when individual participant data are available	When individual participant data are available, comparability is assessed using prespecified baseline covariates at the participant level, and measured differences can be adjusted for. Matching, weighting, stratification or related methods based on propensity scores for study membership can be used to align historical controls with the target population of the prospective trial.	Patient‐level adjustment can mitigate bias arising from measured baseline differences between the selected historical and prospective populations. It can also address bias induced by the study‐selection process when study selection is based on baseline covariates or design‐stage characteristics that are included in the adjustment. The validity of such adjustment requires adequate covariate measurement, overlap, post‐adjustment balance, and appropriate model specification. It cannot eliminate bias due to unmeasured differences or due to ODS.	[15, 17, 30]
5. Finalization and documentation of the historical study set	The historical study set for the primary analysis of the hybrid RCT is finalized before the initiation of the prospective trial. For dynamic borrowing, the candidate set and decision rule are fixed prospectively, although the amount ultimately borrowed may depend on the prospective data. Included and excluded studies, reasons for exclusion, deviations from the prespecified criteria and their justifications, data access limitations, and the historical outcome‐related information available when selection decisions were made are documented.	Different historical subsets produced different operating characteristics under the same borrowing procedures. Reporting only the final selected study set obscures an important part of the design.	[10, 14, 17, 19, 51, 54]
6. Assessment of sensitivity to alternative historical study selections	During trial planning, the operating characteristics of prespecified plausible historical‐control selection mechanisms are evaluated where possible. In the final analysis, source‐selection sensitivity is assessed in addition to borrowing‐model sensitivity. Relevant assessments include prespecified analyses of alternative historical sets, candidates with uncertain eligibility, leave‐one‐study‐out analyses, and temporal restrictions.	Standard borrowing‐model sensitivity analyses are conditional on a fixed historical study set and are unable to assess uncertainty arising from the selection process. Quantification of the impact of different selection mechanisms can inform risk assessment during the hybrid RCT design stage and robustness assessment after prospective data have been collected.	[17, 19, 49, 55]

*Note:* The *Relevance for avoiding bias* column explains each recommendation's role in addressing bias arising from the selection process.

The selection of historical controls provides the basis for statistical borrowing. Dynamic borrowing methods then assess the compatibility of historical information conditional on the evidence supplied to the analysis. However, these methods cannot recover studies that were omitted at the selection stage. The validity of borrowing external controls depends, among other factors, on how the historical study set is constructed and on whether the statistical borrowing model reflects uncertainty about the comparability of the selected historical and prospective controls. This distinction motivates the recommendations in Table 7.

The construction of the historical study set has similarities to the selection of data sets and data sources in evidence‐synthesis approaches such as meta‐analyses and systematic reviews. Because the selection of evidence entering such analyses can affect their results [56], detailed guidance has been developed for study identification and exclusion and for documentation of this process [51, 52, 54]. We distinguish the candidate pool, identified through prespecified search sources and broad screening criteria based on design‐stage information (Recommendations 1 and 2), from the historical study set that remains after the prespecified criteria are applied in a study‐level comparability assessment using relevant information from the completed studies (Recommendation 3). Assessment of a hybrid RCT should consider not only the historical controls included in the analysis but also the candidate pool, the selection process, and the reasons for inclusion or exclusion (Recommendations 2–5).

A further source of bias is that the selection of historical trials and the design of the prospective trial can be interdependent. The selection of historical trials, including their number, size, and outcomes, may inform sample size, allocation ratios, inclusion and exclusion criteria, endpoints, and other design choices. In turn, these design choices may influence which historical studies are suitable for inclusion in the hybrid trial (Recommendation 1). Complete separation of the selection of historical studies and the design of the prospective trial may be unrealistic, in particular when historical results are already publicly available.

Nevertheless, the risk of ODS can be mitigated if the initial selection process is specified based on information that was available at the design stage of the historical trials, such as dated versions of trial protocols, without using historical outcome information generated in the historical trials. A similar design‐stage assessment has been emphasized in the context of federated trials, where differences across protocols are identified and addressed before a conjoined analysis is performed [57].

After the candidate pool of historical studies has been defined, one can implement a comparability assessment at the study level (Recommendation 3). Even when eligibility criteria are prespecified, their application to specific candidate studies often involves clinical judgment and a fully algorithmic study selection may not be feasible. Therefore, there is a risk that the selection may still be influenced by observed values of the primary endpoint, correlated secondary endpoints, or other outcome‐related information. If outcome blinding is not feasible, the information available when inclusion decisions were made should be documented transparently. Note that in contrast to meta‐analyses of RCTs, in the selection of historical controls, even outcome‐independent selection may introduce bias. For example, a selection favoring historical studies based on measured baseline characteristics that predict worse control outcomes than those expected in the prospective trial population can lead to bias. When individual participant data are available, an adjusted analysis based on prespecified baseline covariates can mitigate bias arising from this selection process (Recommendation 4). However, such adjustments cannot remove bias due to unmeasured differences or outcome‐dependent selection of the historical controls. Our simulations considered study‐level selection based on aggregate historical information and did not investigate patient‐level selection.

Bias may arise if the studies are selected completely independently of patient data but based on study characteristics, such as the region or the time period in which they were conducted. It may induce nonexchangeability if these characteristics are associated with differences in control outcomes. Such differences in study design and conduct are much more difficult to adjust for if the differences are not fully reflected in the observed baseline characteristics of trial patients.

Potential bias introduced by the selection of historical trials should be considered separately from bias introduced by the choice of the borrowing model (Recommendation 6). Conventional sensitivity analyses vary borrowing methods, weights for historical data, and heterogeneity assumptions to assess the results conditional on a given historical study set, but they do not assess the sensitivity of results to the selection of historical studies. Plausible alternative historical study sets reflecting reasonable clinical judgments or hypothetical selection mechanisms similar to those in our study can be examined to assess the potential magnitude of selection bias and the robustness of the trial conclusion. A separate analysis using only the concurrent control as the control data should also be included as a benchmark for the impact of historical borrowing, as in our simulation study.

### 5.2 Interpretation of Simulation Findings

This study examined how the selection of external controls affects inference in hybrid randomized controlled trials. When historical and concurrent controls are comparable, borrowing improves efficiency; however, how controls are selected has a substantial impact on the validity of the hybrid RCT. ODS mechanisms consistently increase bias and inflate the Type I error rate for modest gains in power, whereas full and random selection provide a more conservative balance. Power gains from ODS mechanisms are modest because ODS makes historical controls more likely to differ from prospective controls, leading to their down‐weighting in the analysis. The bias patterns were robust across varying levels of between‐trial heterogeneity τ, and different numbers and sizes of historical trials ($k,n_{\mathrm{hc}}$). Similar patterns were observed in sensitivity analyses using a half‐normal prior with a smaller scale for τ in the robust MAP method.

In the simulations, outcomes for both the historical studies and hybrid RCTs were simulated under a range of assumptions. The corresponding operating characteristics, also referred to as marginal operating characteristics, were computed by averaging over the sampling distribution of the historical control datasets, reflecting the long‐run frequency properties of the hybrid RCT design. Additionally, we provided conditional operating characteristics based on observed historical control datasets in the ankylosing spondylitis case study, showing the conditional Type I error rates and conditional power of the hybrid trial planned using the observed historical data. The conditional operating characteristics show the same qualitative behavior as the marginal operating characteristics: ODS mechanisms yield larger estimated treatment effects and higher posterior probabilities of benefit, at the price of increased Type I error rates. Interestingly, the impact of ODS mechanisms on the conditional Type I error rate persists even when the prospective trial has a large sample size of n=3000 (Supplementary Figure S16). This is consistent with our finding for the marginal operating characteristics in the large prospective trial scenario (Supplementary Figures S13 and S14). The impact of ODS mechanisms is not resolved by the larger sample size in the prospective trial.

In the scenario with a linear time trend in the log‐odds of response rates across the historical and prospective trials, all borrowing strategies resulted in inflated Type I error rates. ODS mechanisms caused a further increase. This highlights the need to evaluate temporal drift in the historical pool. If temporal drift in the control response is observed, the inclusion of historical controls may be questionable. One possible way to mitigate biases induced by time trends that cannot be explained by covariate shifts is to include calendar time as a factor in the borrowing model.

The bias caused by ODS mechanisms decreases with less flexible selection mechanisms. For example, monotone selection—where all preceding historical trials are also excluded if a later trial is excluded—results in lower bias. This is illustrated for the historical data selected by the optimal‐power mechanism in the case of $k=8,n_{\mathrm{hc}}=30$ (see Supplementary Figures S13 and S14). Using a more restrictive selection that considers the temporal order of the historical trials will generally lead to less Type I error inflation than the other non‐monotone ODS mechanisms, but overall fewer historical trials will be used (Supplementary Table S5).

The statistical methods proposed for hybrid designs rely on the assumption that the historical controls and the prospective controls are exchangeable. However, under ODS, even if exchangeability holds for the initial pool of historical controls, this assumption is violated for the selected trials. Because the selection of controls typically occurs after these trials have been performed, it may be impossible to demonstrate that the selection was not influenced by outcome data. Historical control data are often already publicly available in reports, publications, or registries. Prior knowledge of historical data may influence the selection process, either consciously or unconsciously, introducing a selection bias. Even if only data on a different but correlated endpoint are available and influence selection, bias will be introduced. Both Bayesian and frequentist testing procedures are affected by such ODS. While in this work we focused on frequentist operating characteristics, the outcome‐dependent selection of historical controls also invalidates Bayesian analyses. Outcome‐dependent selection changes the likelihood function, and therefore Bayesian posterior computations that do not account for this will be flawed. Selecting historical trials based on their outcomes induces data‐dependent priors, which compromises the validity of posterior probabilities and other Bayesian metrics such as the average Type I error rate [58]. Therefore, Bayesian inference may also be invalid under outcome‐dependent selection.

It is well known that neither Bayesian nor frequentist two‐step tests can strictly control the frequentist Type I error rate when incorporating historical data in the decision making [46, 58] if the distribution of the historical controls differs from the concurrent controls. This is an additional and distinct source of bias. Generally, having a higher risk under certain scenarios might be justified, e.g., in clinical trials with limited sample size such as in rare diseases. We show that if there is ODS, the operating characteristics usually presented will be biased, e.g., underestimating the actual risk of a false positive decision. Our supplementary analysis further illustrates that the power gain from borrowing should be interpreted together with its corresponding Type I error rate. Recalibrating the borrowing procedures to comparable or nominal Type I error levels reduced the benefit of borrowing, with power differences generally below zero, consistent with the findings from Kopp‐Schneider et al. [59] (Supporting Information S5; Supplementary Figures S17 and S18).

Unequal randomization warrants close attention in hybrid RCTs. Allocating more patients to the experimental arm can be motivated by ethical, recruitment, safety, or practical considerations, for example when treatment options are limited [60, 61]. However, this rationale is contested because treatment superiority is unknown at trial initiation. Potential benefits to current participants must be weighed against reduced statistical precision and less reliable evidence for future patients [62, 63, 64]. If the experimental treatment is actually inferior, such allocation exposes more participants to the worse treatment. For a fixed total sample size, unequal treatment:control allocation reduces concurrent control information and increases the variance of the treatment‐control comparison when using only the concurrent data. In hybrid RCTs, borrowing external controls can offset this loss of precision, but it also increases reliance on historical controls and reduces the concurrent information available to assess their comparability. If the historical control pool has been selected in an outcome‐dependent manner, unequal randomization may therefore amplify selection bias. Unequal allocation may also reflect investigator bias or optimism about the experimental treatment and should be justified transparently, especially in confirmatory settings. In our simulations, sensitivity to ODS increases as the prospective control arm shrinks: ODS of historical controls inflates treatment‐effect estimates and false positive rates. Beyond operating characteristics, this carries an ethical cost, as misleading evidence may affect treatment decisions for future patients.

### 5.3 Limitations

This work has limitations. Our implementation of TTP uses a pooled pre‐test in Equation (1). An alternative TTP approach is to test each historical control separately against the prospective control (with or without multiplicity adjustment). We implemented the robust MAP with a fixed robustness weight and one family of priors (half‐normal) for the heterogeneity parameter τ; other robustness weights, other MAP methods such as the modified robust MAP [65], and alternative priors for τ could have been used [66]. Furthermore, we focused on binary endpoints. However, ODS is expected to have a similar impact for time‐to‐event or continuous outcomes.

### 5.4 Conclusion

This study shows that while hybrid RCTs can improve the efficiency of a stand‐alone, underpowered RCT, the prespecification of external data sources remains an unresolved issue. Detailed documentation and justification of the selection process are needed, and transparency of the entire process is crucial. All potentially relevant external controls should be considered, and the selection process should be carefully assessed and reported [34]. If historical trials are excluded, additional sensitivity analyses should be conducted (Recommendation 6). For example, a hypothetical omitted historical control study could be introduced in a tipping‐point analysis to determine how its results would need to differ in order to change the original conclusion. This might help to quantify the potential selection bias.

## Author Contributions

F.K. and M.P. conceived this research. All authors contributed to the planning and design of the simulation study. H.C.C. wrote the first draft of the manuscript under the supervision of M.P. and F.K. H.C.C. performed the simulations and statistical analyses. F.K. and M.P. revised the manuscript. All authors discussed the results, provided comments, reviewed and approved the manuscript.

## Funding

This work was supported by European Union's Horizon‐MSCA‐2022‐DN‐01 program, under SHARE‐CTD grant agreement No. 101120360.

## Disclosure

The authors have nothing to report.

## Conflicts of Interest

The authors declare no conflicts of interest.

## Supporting information


**Figure S1:** Supplementary Type I error rates of various selection mechanisms, to show the impact of randomization ratio (*x*‐axis, total sample size in the prospective trial is fixed at $n_{\text{total}}=2*n_{\mathrm{hc}}$) and between‐trial heterogeneity τ (columns); Type I error rates of different numbers of historical trials *k* and sample size per historical control arm $n_{\mathrm{hc}}$ are provided. Dashed line: outcome‐dependent selections. Results from both robust MAP and TTP are provided.
**Figure S2:** Supplementary Type I error rates of various selection mechanisms, to show the impact of numbers of historical trials k (x‐axis) and sample size per historical control arm nhc (circle: 30, triangle: 90); Type I error rates of different levels of between‐trial heterogeneity τ are provided, with fixed 1:1 randomization ratio. Dashed line: outcome‐dependent selections. Results from both robust MAP and TTP are provided.
**Figure S3:** Supplementary Power of various selection mechanisms, to show the impact of randomization ratio (x‐axis, total sample size in the prospective trial is fixed at ntotal=2*nhc) and between‐trial heterogeneity τ (columns); Power of different numbers of historical trials k and sample size per historical control arm nhc are provided. Dashed line: outcome‐dependent selections. Results from both robust MAP and TTP are provided.
**Figure S4:** Supplementary Power of various selection mechanisms, to show the impact of numbers of historical trials k (x‐axis) and sample size per historical control arm nhc (circle: 30, triangle: 90); Power of different levels of between‐trial heterogeneity τ are provided, with fixed 1:1 randomization ratio. Dashed line: outcome‐dependent selections. Results from both robust MAP and TTP are provided.
**Figure S5:** Supplementary Bias of various selection mechanisms under H1, to show the impact of randomization ratio (x‐axis, total sample size in the prospective trial is fixed at ntotal=2*nhc) and between‐trial heterogeneity τ (columns); Bias of different numbers of historical trials k and sample size per historical control arm nhc are provided. Dashed line: outcome‐dependent selections. Results from both robust MAP and TTP are provided.
**Figure S6:** Supplementary Bias of various selection mechanisms under H1, to show the impact of numbers of historical trials k (x‐axis) and sample size per historical control arm nhc (circle: 30, triangle: 90); Bias of different levels of between‐trial heterogeneity τ are provided, with fixed 1:1 randomization ratio. Dashed line: outcome‐dependent selections. Results from both robust MAP and TTP are provided.
**Figure S7:** Supplementary Bias of various selection mechanisms under H0, to show the impact of randomization ratio (x‐axis, total sample size in the prospective trial is fixed at ntotal=2*nhc) and between‐trial heterogeneity τ (columns); Bias of different numbers of historical trials k and sample size per historical control arm nhc are provided. Dashed line: outcome‐dependent selections. Results from both robust MAP and TTP are provided.
**Figure S8:** Supplementary Bias of various selection mechanisms under H0, to show the impact of numbers of historical trials k (x‐axis) and sample size per historical control arm nhc (circle: 30, triangle: 90); Bias of different levels of between‐trial heterogeneity τ are provided, with fixed 1:1 randomization ratio. Dashed line: outcome‐dependent selections. Results from both robust MAP and TTP are provided.
**Figure S9:** Supplementary RMSE of various selection mechanisms under H1, to show the impact of randomization ratio (x‐axis, total sample size in the prospective trial is fixed at ntotal=2*nhc) and between‐trial heterogeneity τ (columns); RMSE of different numbers of historical trials k and sample size per historical control arm nhc are provided. Dashed line: outcome‐dependent selections. Results from both robust MAP and TTP are provided.
**Figure S10:** Supplementary RMSE of various selection mechanisms under H1, to show the impact of numbers of historical trials k (x‐axis) and sample size per historical control arm nhc (circle: 30, triangle: 90); RMSE of different levels of between‐trial heterogeneity τ are provided, with fixed 1:1 randomization ratio. Dashed line: outcome‐dependent selections. Results from both robust MAP and TTP are provided.
**Figure S11:** Supplementary RMSE of various selection mechanisms under H0, to show the impact of randomization ratio (x‐axis, total sample size in the prospective trial is fixed at ntotal=2*nhc) and between‐trial heterogeneity τ (columns); RMSE of different numbers of historical trials k and sample size per historical control arm nhc are provided. Dashed line: outcome‐dependent selections. Results from both robust MAP and TTP are provided.
**Figure S12:** Supplementary RMSE of various selection mechanisms under H0, to show the impact of numbers of historical trials k (x‐axis) and sample size per historical control arm nhc (circle: 30, triangle: 90); RMSE of different levels of between‐trial heterogeneity τ are provided, with fixed 1:1 randomization ratio. Dashed line: outcome‐dependent selections. Results from both robust MAP and TTP are provided.
**Table S1:** Supplementary Effective Sample Size (ESS) of robust MAP prior across selection mechanisms (excluding optimal power selection), half‐normal prior for between‐trial heterogeneity τ∼HN (1) as default. k: numbers of historical trials; nhc: sample size per historical control arm. Note that the total sample size in the prospective trial is fixed at ntotal=2*nhc.
**Table S2:** Supplementary Effective Sample Size (ESS) across selection mechanisms (excluding optimal power selection), half‐normal prior between‐trial heterogeneity for τ∼HN(0.5) as sensitivity analysis. k: numbers of historical trials; nhc: sample size per historical control arm. Note that the total sample size in the prospective trial is fixed at ntotal=2*nhc.
**Table S3:** Supplementary Effective Sample Size (ESS) for optimal power selection by randomization ratio, half‐normal prior for between‐trial heterogeneity τ∼HN (1) as default. k: numbers of historical trials; nhc: sample size per historical control arm. In contrast to the other selection mechanisms, optimal power selection depends on the design specification of the prospective trial, such as allocation ratio. Note that the total sample size in the prospective trial is fixed at ntotal=2*nhc.
**Table S4:** Supplementary Effective Sample Size (ESS) for optimal power selection by randomization ratio, under the alternative hypothesis; half‐normal prior for between‐trial heterogeneity τ∼HN(0.5) as sensitivity analysis. k: numbers of historical trials; nhc: sample size per historical control arm. In contrast to the other selection mechanisms, optimal power selection depends on the design specification of the prospective trial, such as allocation ratio. Note that the total sample size in the prospective trial is fixed at ntotal=2*nhc.
**Table S5:** Supplementary As Table 5 (k = 8 historical trials, with sample size per historical control arm nhc=30, and moderate between‐trial heterogeneity τ = 0.30), with an additional row reporting the monotone selection constraint applied to the optimal power selection. Monotone selection (red): if a later trial is excluded, all earlier trials are also excluded.
Exchangeable: reference risk difference *RD* = 0.20.
Time‐trend: linear drift in the historical data.
Large prospective trial: prospective trial with *RD* = 0.0635 with *n* = 250 per arm.
*RD*: treatment effect measure, risk differences between the treatment and control arm.
All values are **percentages**. T1E: Type I error rate at nominal one‐sided level 2.5%. Bias: percentage‐point deviation of posterior mean RD from the truth, under $H_{1}$, calculated by $\frac{1}{n_{\mathrm{sim}}}\sum_{r=1}^{n_{\mathrm{sim}}}\left(\hat{R}D^{\left(r\right)}-\mathrm{RD}\right)\times 100$. RMSE: root mean square error, under *H*_1_, calculated by $\sqrt{\frac{1}{n_{\mathrm{sim}}}\sum_{r=1}^{n_{\mathrm{sim}}}{\left(\hat{R}D^{\left(r\right)}-\mathrm{RD}\right)}^{2}}\times 100$.
**Figure S13:** Supplementary Performance under the null hypothesis with k = 8 historical trials, with sample size per historical control arm nhc = 30, and moderate heterogeneity between historical trials (τ = 0.30). Scenario 1a + 1b: under H0, RD = 0. Scenario 2 Time‐trend: linear drift in historical data. Scenario 3 Large prospective trial: under H0, RD = 0 and with n = 250 per arm. Panels show Type I error rate, Bias (H0), and RMSE (H0). x‐axis: Prospective control response rate; the vertical line at x = 0.20 indicates exchangeability (Scenario 1a). Dashed line: outcome‐dependent selections. Cyan solid line: Monotone selection, mitigating the impact of optimal power selection (orange dashed line).
**Figure S14:** Supplementary Performance under the alternative hypothesis with k = 8 historical trials, with sample size per historical control arm nhc = 30, and moderate heterogeneity between historical trials (τ = 0.30). Scenario 1a + 1b: reference risk difference RD = 0.20. Scenario 2 Time‐trend: linear drift in historical data. Scenario 3 Large prospective trial: RD = 0.0635 with n = 250 per arm. Panels show Power, Bias (H1), and RMSE (H1). x‐axis: Prospective control response rate; the vertical line at x = 0.20 indicates exchangeability (Scenario 1a). Dashed line: outcome‐dependent selections. Cyan solid line: Monotone selection, mitigating the impact of optimal power selection (orange dashed line).
**Figure S15:** Supplementary Selection results in the case study. Left: Forest plot of AS data, centered at 0.25; Right: selected trials with indicators (✓).
**Figure S16:** Supplementary Conditional operating characteristics of different selection mechanisms based on the same historical pool and varying prospective settings. (a) Conditional Type I error rates and power when prospective trial planned to have total sample size ntotal=30. (b).
Conditional Type I error rates and power when prospective trial planned to have total sample size $n_{\text{total}}=60$. (c) Conditional Type I error rates when prospective trial planned to have total sample size $n_{\text{total}}=300$ (left) and $n_{\text{total}}=3000$ (right), power is not shown here as under this sample size the power will be very close to 1. The black dashed line marks the worst (max conditional Type I error) selection.
**Table S6:** Supplementary Results of applying various selection mechanisms to the ankylosing spondylitis (AS) case study trial data, analyzed with TTP.
Estimate: Risk difference between treatment and control from two‐sample test for equality of proportions with continuity correction. 95% CI: confidence interval from two‐sample test for equality of proportions with continuity correction. *p* value: Obtained from Fisher's exact test with one‐sided α=0.025.
**Table S7:** Supplementary Calibrated decision thresholds for the test‐then‐pool and robust MAP analyses for the setting with τ = 0.30 and 1:1 randomization.
For test‐then‐pool, treatment benefit is declared when $p<\alpha_{s}^{*}$; a smaller value therefore represents a more stringent decision rule. For robust MAP, treatment benefit is declared when $\mathrm{Pr}\left(\pi_{t}-\pi_{c}>0|D_{0},{ℋ}_{s}\right)>c_{s}^{*}$. Here, a larger value represents a more stringent decision rule. The original thresholds were α=0.025 and c=0.975, respectively.
**Figure S17:** Supplementary Marginal operating characteristics of selection mechanisms with an initial set of k = 8 historical control trials each with a sample size of nhc = 30 and assuming a moderate between‐trial heterogeneity of τ = 0.30, prospective trial total sample size ntotal=60. For the borrowing procedures, Type I error rate and power were computed using calibrated decision thresholds that restricted the maximum Type I error rate to the corresponding reference level. (a)–(c) H0: type I error rate, bias, RMSE. (d)–(f) H1: power, bias, RMSE. x‐axis: prospective control response rate; the vertical line at x = 0.20 indicates exchangeability, where maximum amount of borrowing is appropriate; dashed lines denote outcome‐dependent selections.
**Figure S18:** Supplementary Power difference Δsπc between each borrowing procedure and the separate analysis for an initial set of k = 8 historical control trials each with sample size nhc = 30, moderate between‐trial heterogeneity τ = 0.30, prospective trial total sample size ntotal=60, and 1:1 randomization. Shaded bands denote approximate 95% Monte Carlo intervals. The horizontal dashed line indicates equal power, and the vertical line at x = 0.20 indicates exchangeability between historical and prospective controls.

## Data Availability

The code and supporting files for the simulation study and case‐study analyses are openly available in Zenodo at https://doi.org/10.5281/zenodo.22939727. The simulated data are generated by the code provided in the repository. The historical data used in the case study are publicly available in the AS dataset distributed with the RBesT R package.

## Appendix A Optimal Power Selection

To select the historical subset that maximizes conditional power at the planning effect size, we evaluate all $2^{k}-1$ nonempty subsets S⊆ℋ and compare their maximum conditional power with that of the separate analysis. If no nonempty subset provides higher conditional power, the empty subset is selected and treated as the separate analysis. For computational feasibility in this selection step we use a pooled informative prior for the control arm (not the robust MAP).

**Setup**. Let $ℋ=\left\{H_{1},\ldots ,H_{k}\right\}$ be the pool of historical control trials. For any subset S⊆ℋ, let x(S) and $n_{H}\left(S\right)$ be the total number of responders and total sample size aggregated over S. We take

$$\pi_{c}|S∼\text{Beta}\;\left(x\left(S\right),n_{H}\left(S\right)-x\left(S\right)\right),\;\pi_{t}∼\text{Beta}\left(1,1\right).$$

For a planned two–arm trial, we denote responders' counts by $\left(y_{t},y_{c}\right)$ and sample sizes by $\left(n_{t},n_{c}\right)$, $D_{0}=\left(y_{t},y_{c};n_{t},n_{c}\right)$ as the notation of prospective trial data. The empty subset was treated as the separate analysis, using a Beta(1,1) prior. For numerical implementation, a zero beta shape parameter was replaced by ε=0.0001. This correction was required only for subsets containing historical trials with no responders. No historical trial with full responders occurred in the simulations.

**Posterior success rule and decision boundary**. With one–sided success threshold γ (here γ=0.975), declare success when $\mathrm{Pr}\left(\pi_{t}>\pi_{c}|D_{0},S\right)>\gamma .$ Let $P_{y_{t},y_{c}}^{S}=\mathrm{Pr}\left(\pi_{t}>\pi_{c}|D_{0},S\right)$ under the priors above. By Beta–Binomial conjugacy,

$$\pi_{t}|y_{t}∼\text{Beta}\left(\alpha_{1},\beta_{1}\right),\;\pi_{c}|y_{c},S∼\text{Beta}\left(\alpha_{2},\beta_{2}\right),$$

with

$$\alpha_{1}=1+y_{t},\;\beta_{1}=1+n_{t}-y_{t},\;\alpha_{2}=x\left(S\right)+y_{c},\;\beta_{2}=n_{H}\left(S\right)-x\left(S\right)+n_{c}-y_{c}.$$

Then, for $\alpha_{1}\in \mathbb{N}$, the posterior superiority probability admits the series (Pham‐Gia et al. [67]):

$$P_{y_{t},y_{c}}^{S}\;=\;\sum_{k=0}^{\alpha_{1}-1}\frac{B\left(\alpha_{2}+k,\;\beta_{1}+\beta_{2}\right)}{\left(\beta_{1}+k\right)B\left(\alpha_{2},\beta_{2}\right)\;B\left(k+1,\beta_{1}\right)}.$$

Define the boundary as the largest treatment count not crossing γ,

(A1)
$$d_{1}^{S}\left(y_{c}\right)=\max\left\{\;y_{t}\leq n_{t}:\;P_{y_{t},y_{c}}^{S}\leq \gamma \;\right\}.$$

so success occurs if $y_{t}>d_{1}^{S}\left(y_{c}\right)$.

**Exact conditional power for subset selection**. At planning values $\left(\pi_{t}^{⋆},\pi_{c}^{⋆}\right)=\left(0.4,0.2\right)$,

(A2)
$$\text{Power}\left(S\right)=\sum_{y_{c}=0}^{n_{c}}\left(\begin{array}{c} n_{c}\\ y_{c} \end{array}\right){\left(0.2\right)}^{y_{c}}{\left(0.8\right)}^{n_{c}-y_{c}}\sum_{y_{t}=d_{1}^{S}\left(y_{c}\right)+1}^{n_{t}}\left(\begin{array}{c} n_{t}\\ y_{t} \end{array}\right){\left(0.4\right)}^{y_{t}}{\left(0.6\right)}^{n_{t}-y_{t}}$$

(note the inner sum starts at $d_{1}^{S}\left(y_{c}\right)+1$ since $d_{1}^{S}$ is the *largest failing* $y_{t}$).

**Optimal subset.**

$$S^{⋆}\;=\;\underset{S\subseteq ℋ}{\arg\max}\;\text{Power}\left(S\right),$$

that is, the historical combination that maximizes conditional power at (0.4,0.2) in the simulations under the pooled‐Beta informative control prior. In the case study, the same subset‐search principle was applied at $\left(\pi_{t}^{⋆},\pi_{c}^{⋆}\right)=\left(0.60,0.25\right)$, using the analysis prior appropriate to each candidate subset as described in Supporting Information S2.
