# Supplementary material for: Selection Bias in Hybrid Randomized Controlled Trials Using External Controls: A Simulation Study
Source: Stat Med. 2026 Nov 13;45(25-27):e70819. doi: 10.1002/sim.70819 (PMC13761282; doi:10.1002/sim.70819)
Supplement: Supplementary file 1 [file SIM-45-0-s001.pdf]

Supplementary Material for:
Selection Bias in Hybrid Randomized Controlled Trials Using External Controls: A Simulation Study

Han Chang Chiam, Franz König, and Martin Posch

Center for Medical Data Science, Medical University of Vienna, Vienna, Austria

September 2026

Corresponding author: Prof. Martin Posch, Center for Medical Data Science, Medical University of Vienna, Spitalgasse 23, 1090 Vienna, Austria. Email: martin.posch@meduniwien.ac.at

Contents

S1 Robust Meta-Analytic-Predictive (MAP) prior and posterior probability of superiority (binary endpoint) 3

S2 Simulation study 5

S3 Additional information for case study 25

S4 Design-stage quantities (binary endpoint; Beta(1,1) for treatment; robust MAP for control) 28

S5 Power comparison after type I error rate calibration 30

Overview

This supplementary material is organized as follows:

S1 **Robust Meta-Analytic-Predictive (MAP) prior and posterior probability of superiority (binary endpoint)** details the derivation of the robust MAP prior (main manuscript Section 2) from the historical controls, the subsequent posterior update, and the computation of posterior superiority probabilities.

S2 **Simulation study** provides implementation details of the simulation, along with figures and tables supporting the results presented in Section 3.2 of the main manuscript. This section also includes supporting information on monotone selection discussed in Section 5 of the main manuscript.

S3 **Additional information for the case study** presents further details for the case study in Section 4 of the main manuscript, including the trials selected after applying the hypothetical selection mechanisms, conditional operating characteristics under different prospective trial settings, and the analysis results of applying various selection mechanisms to the AS trial using TTP.

S4 **Design-stage quantities (binary endpoint; Beta(1,1) for treatment; robust MAP for control)** describes the calculation of the conditional operating characteristics and probability of success in Section 4.1 of the main manuscript.

S5 **Calibrated power comparison** presents a sensitivity analysis that recalibrates each borrowing procedure to match the maximum empirical Type I error rate of the separate analysis before comparing power. Calibration adjusts the significance level of Fisher's exact test for test-then-pool and the posterior probability threshold for declaring treatment benefit for robust MAP.

S1 Robust Meta-Analytic-Predictive (MAP) prior and posterior probability of superiority (binary endpoint)

Notation. For each study arm let $\pi \in (0, 1)$ denote the response probability and $\theta = \text{logit}(\pi)$ the log-odds. Historical control arms are indexed by $i = 1, \ldots, k$ with random counts $Y_i \sim \text{Bin}(n_i, \pi_i)$ and $\theta_i = \text{logit}(\pi_i)$. In the prospective trial we write $\mathcal{D}_0 = (y_t, y_c;\, n_t, n_c)$ for the observed treatment/control counts and sample sizes.

Meta-Analytic-Predictive (MAP) prior on the probability scale. We fit a normal hierarchical model on the log-odds scale, with normal prior for the mean μ of log-odds and half-normal prior for the between-trial heterogeneity τ

$$\theta_i \mid \mu, \tau^2 \sim \mathcal{N}(\mu, \tau^2), \qquad \mu \sim \mathcal{N}(0, 2), \quad \tau \sim \text{Half}-\text{Normal}(1).$$

Given the historical data $\{\theta_i\}$ the posterior predictive distribution of θ_{new} for a new study on the log-odds scale is

$$p(\theta_{\text{new}} \mid \text{hist}) = \iint \mathcal{N}(\theta_{\text{new}}; \mu, \tau^2)\, p(\mu, \tau \mid \text{hist})\, d\mu\, d\tau.$$

Mapping to the probability scale via $\pi_{\text{new}} = \text{logit}^{-1}(\theta_{\text{new}})$ and approximating by a finite Beta mixture (Dalal & Hall [1]) yields

$$\widehat{p}_H(\pi_{\text{new}}) = \sum_{\ell=1}^{K} w_\ell\, \text{Beta}(\pi_{\text{new}} \mid a_\ell, b_\ell), \qquad w_\ell > 0,\ \sum_{\ell=1}^{K} w_\ell = 1. \tag{S1.1}$$

(Here $\ell = 1, \ldots, K$ indexes mixture components and is unrelated to the number of historical trials k.)

Robustification. To account for potential prior–data conflict, we mix the MAP prior with a vague component:

$$\begin{aligned} \widehat{p}_{H,\text{robust}}(\pi_{\text{new}}) = w_R\, \text{Beta}(\pi_{\text{new}} \mid 1, 1) + (1 - w_R) \sum_{\ell=1}^{K} w_\ell\, \text{Beta}(\pi_{\text{new}} \mid a_\ell, b_\ell), \\ w_R \in (0, 1). \end{aligned} \tag{S1.2}$$

(with $w_R = 0.1$ in our simulations). In what follows we identify the new-study control with the prospective RCT control, i.e. $\pi_{\text{new}} \equiv \pi_c$.

Posterior update for the prospective control. Let the prospective control observe y_c of n_c events. By Beta–Binomial conjugacy, each Beta component updates to a Beta posterior and the mixture weights are updated by Bayes factors. Writing $m_\star(y_c)$ for the component-wise marginal likelihood,

$$m_\star(y_c) \;=\; \binom{n_c}{y_c} \frac{\text{Beta}(a_\star + y_c,\ b_\star + n_c - y_c)}{\text{Beta}(a_\star, b_\star)}, \qquad \star \in \{0,\ 1, \ldots, K\},$$

with $(a_0, b_0) = (1, 1)$. (The binomial coefficient cancels in all weight ratios.)

The posterior for π_c is the finite mixture

$$\pi_c \mid y_c \;\sim\; \widetilde{w}_R\, \text{Beta}(1 + y_c,\ 1 + n_c - y_c) \;+\; (1 - \widetilde{w}_R) \sum_{\ell=1}^{K} \widetilde{w}_\ell\, \text{Beta}(a_\ell + y_c,\ b_\ell + n_c - y_c),$$

with updated weights

$$\widetilde{w}_R \;=\; \frac{w_R\, m_0(y_c)}{w_R\, m_0(y_c) + (1 - w_R) \sum_{\ell=1}^{K} w_\ell\, m_\ell(y_c)}, \qquad \widetilde{w}_\ell \;=\; \frac{w_\ell\, m_\ell(y_c)}{\sum_{j=1}^{K} w_j\, m_j(y_c)}.$$

Treatment arm. We use an independent Beta prior $\pi_t \sim \mathrm{Beta}(a_{t0}, b_{t0})$ (e.g. Beta(1, 1)), with current treatment data $y_t \sim \mathrm{Bin}(n_t, \pi_t)$. Then

$$\pi_t \mid y_t \sim \mathrm{Beta}(a_{t0} + y_t,\ b_{t0} + n_t - y_t).$$

Joint posterior in the prospective RCT. With independent priors across arms, the joint posterior factorizes as

$$\begin{aligned} p(\pi_t, \pi_c \mid y_t, y_c) \propto \mathrm{Bin}(y_t \mid n_t, \pi_t)\,\mathrm{Bin}(y_c \mid n_c, \pi_c)\,\mathrm{Beta}(\pi_t \mid a_{t0}, b_{t0}) \\ \times \Big[w_R\,\mathrm{Beta}(\pi_c \mid 1, 1) + (1 - w_R)\sum_{\ell=1}^{K} w_\ell\,\mathrm{Beta}(\pi_c \mid a_\ell, b_\ell)\Big], \end{aligned} \tag{S1.3}$$

which yields

$$\pi_t \mid y_t \sim \mathrm{Beta}(a_{t0} + y_t,\ b_{t0} + n_t - y_t),$$

and

$$\pi_c \mid y_c \sim \widetilde{w}_R\,\mathrm{Beta}(1 + y_c,\ 1 + n_c - y_c) + (1 - \widetilde{w}_R)\sum_{\ell=1}^{K} \widetilde{w}_\ell\,\mathrm{Beta}(a_\ell + y_c,\ b_\ell + n_c - y_c),$$

with the updated weights $\widetilde{w}_R, \widetilde{w}_\ell$ defined via the component-wise marginal likelihoods $m_\star(y_c)$ as in the previous paragraph.

Posterior probability of superiority. Define success as $\pi_t > \pi_c$ on the probability scale. By independence across arms and mixture decomposition,

$$\Pr(\pi_t > \pi_c \mid \mathcal{D}_0, \mathcal{H}) = \widetilde{w}_R(y_c)\ \Pr\big[X > Y_0\big]\ +\ \big(1 - \widetilde{w}_R(y_c)\big)\sum_{\ell=1}^{K} \widetilde{w}_\ell(y_c)\ \Pr\big[X > Y_\ell\big], \tag{S1.4}$$

where

$$X \sim \mathrm{Beta}(a_{t0}{+}y_t,\ b_{t0}{+}n_t{-}y_t), \quad Y_0 \sim \mathrm{Beta}(1{+}y_c,\ 1{+}n_c{-}y_c), \quad Y_\ell \sim \mathrm{Beta}(a_\ell{+}y_c,\ b_\ell{+}n_c{-}y_c).$$

Each term $\Pr[X > Y_\bullet]$ admits the integral representation

$$\Pr[X > Y]\ =\ \int_0^1\int_0^x \mathrm{Beta}(x \mid \alpha, \beta)\,\mathrm{Beta}(y \mid \gamma, \delta)\ dy\,dx,$$

which can be evaluated numerically; when α is a positive integer, closed-form finite series in Beta functions are available [2].

S2 Simulation study

In the simulation both historical trials and prospective trial data were simulated, under various assumptions, and the operating characteristics are interpreted as unconditional measures.

Please note that for the threshold and optimal power selection mechanisms, the retained historical pool may contain no or only one trial. In this case the robust MAP framework is not appropriate. We therefore adapt the borrowing strategy in these cases. If no historical controls are selected, the prospective trial is analysed separately as described in Section 2 in the main manuscript. If only one historical control is selected the robust MAP prior is replaced with a *robust mixture prior*, i.e. a convex combination of the vague prior Beta$(1,1)$ and the posterior Beta$(1 + x_h,\ 1 + n_h - x_h)$ of the retained trial (see Callegaro 2023 [3]).

$$\hat{p}_{H,\mathrm{RMP}}(\pi_{\mathrm{new}}) = w_R\, \mathrm{Beta}(1,1) + (1 - w_R)\, \mathrm{Beta}\big(1 + x_h,\ 1 + n_h - x_h\big), \qquad w_R = 0.1.$$

This ensures that Bayesian borrowing remains well-defined under every selection outcome while preserving comparability across selection mechanisms.

The first part of this section investigated the impact of most parameters (highlighted in red) in Table 2 (from main manuscript, also shown below); Second part in monotone selection we fixed the parameters $k = 8$ historical trials, with sample size per historical control arm $n_{hc} = 30$, 1:1 randomization ratio and moderate between-trial heterogeneity $\tau = 0.30$ to investigate different scenarios as in Table 3 (from main manuscript, also shown below). Scenario (1a +1b) can be compared to Scenario 2 to show the impact of temporal drift in historical trials, using the same prospective trial data. On the other hand Scenario (1a +1b) can also be compared to Scenario 3 to show the impact of large prospective trial, as these 2 scenarios used same historical studies pool.

Table 2: Factors varied in the simulation, classified as Design (D) or Assumption (A), with investigated values and description.

**Factor**	**Type**	**Investigated values**	**Description**
τ	A	0.10, 0.30, 0.50	Between-trial heterogeneity; values motivated by Neuenschwander et al. (2010) [4].
k	A	4, 8	Number of historical trials (control arm only).
n_{hc}	A	30, 90	Historical control sample size per trial.
n_{total}	D	60, 180	Prospective trial total sample size.
Randomization ratio	D	1:1, 2:1, 3:1	Allocation ratio of prospective trial (treatment:control).
π_c	A	0.15-0.75 (step 0.05)	True prospective control response rate.

Type: D = Design; A = Assumption.

Scenario 1a: Exchangeable This part supports the findings on unconditional type I error rates, power, bias (under H_0, H_1), root mean square error(RMSE, under H_0, H_1), effective sample size (robust MAP) reported in the main text Section 3.2.1, when historical control response rates and prospective control response rate are *exchangeable*. The presented results assume $E(\pi_{hc}) = E(\pi_c) = 0.20$.

Table 3: Simulation scenarios for prospective trial. The scenario numbers are used for reference in the Results section.

**Scenario**	**Name**	**Description**
1a	Exchangeable	No distributional shift, $E(\pi_{hc}) = E(\pi_c) = 0.20$, historical and prospective control response rates π_{hc}, π_c are exchangeable.
1b	Prospective-historical distributional shift	$E(\pi_{hc}) = 0.20$, prospective $E(\pi_c)$ varied from 0.15 to 0.75 (step 0.05). Represents prior-data conflict.
2	Time trend	Linear logit drift ($\beta_2 = -0.05$) across historical trials for $k = 8$, $n_{hc} = 30$; prospective trial fixed at $E(\pi_c) = 0.20$, earliest historical trial $E(\pi_{hc}) \approx 0.14$.
3	Large prospective trial	Larger prospective trial with total sample size (n_{total}) = 500 with smaller effects RD = (0.0635, 0.1152), calibrated so that the no-borrowing design achieves ≈ 39% and 84% power, respectively.

RD denotes risk differences in prospective trial used for calibration. π_{hc}: historical control trials response rate; π_c: prospective trial control response rate

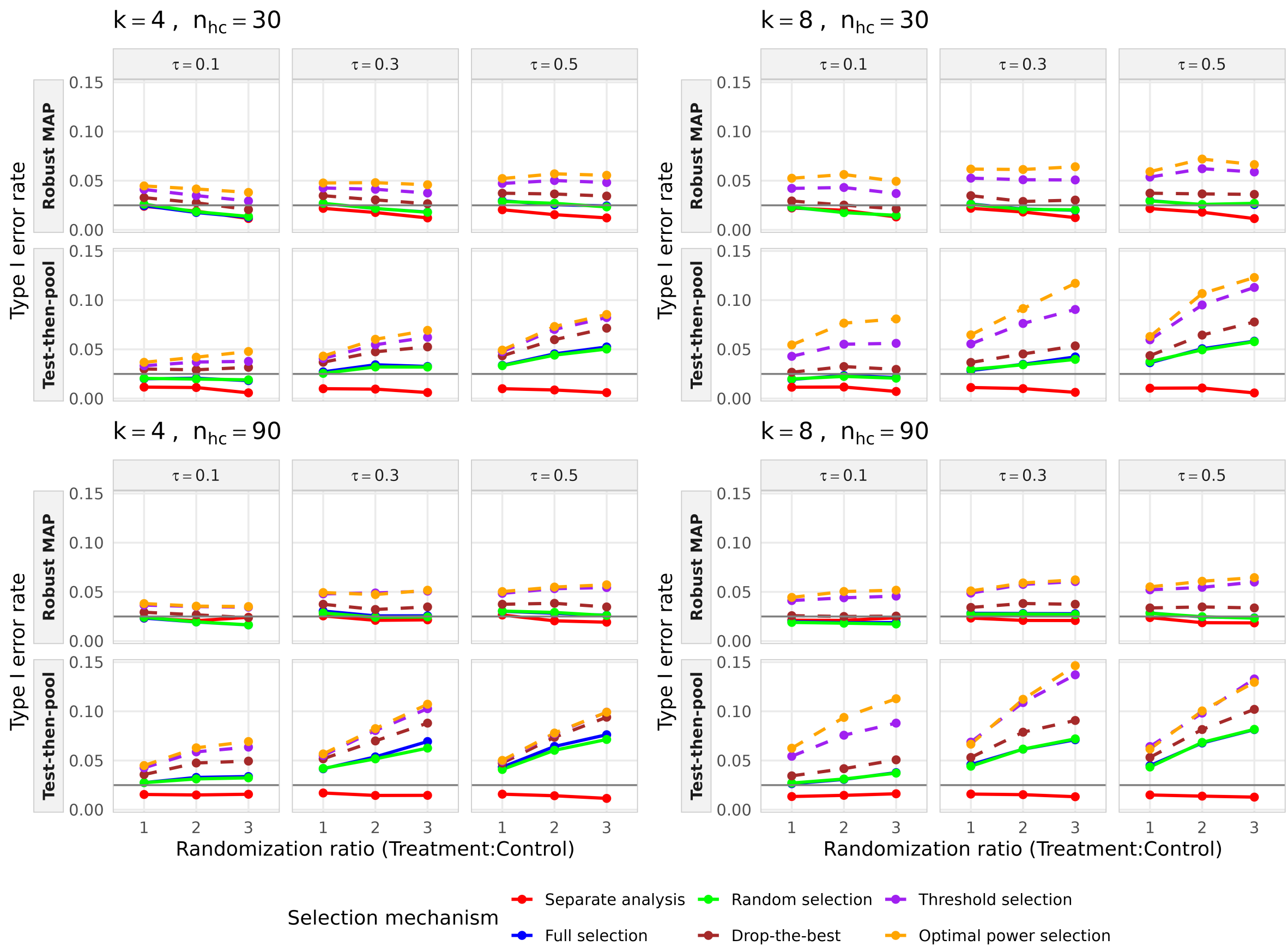

Supplementary Figure S1: Type I error rates of various selection mechanisms, to show the impact of randomization ratio (x-axis, total sample size in the prospective trial is fixed at $n_{total} = 2 * n_{hc}$) and between-trial heterogeneity τ (columns); Type I error rates of different numbers of historical trials k and sample size per historical control arm n_{hc} are provided. Dashed line: outcome-dependent selections. Results from both robust MAP and TTP are provided.

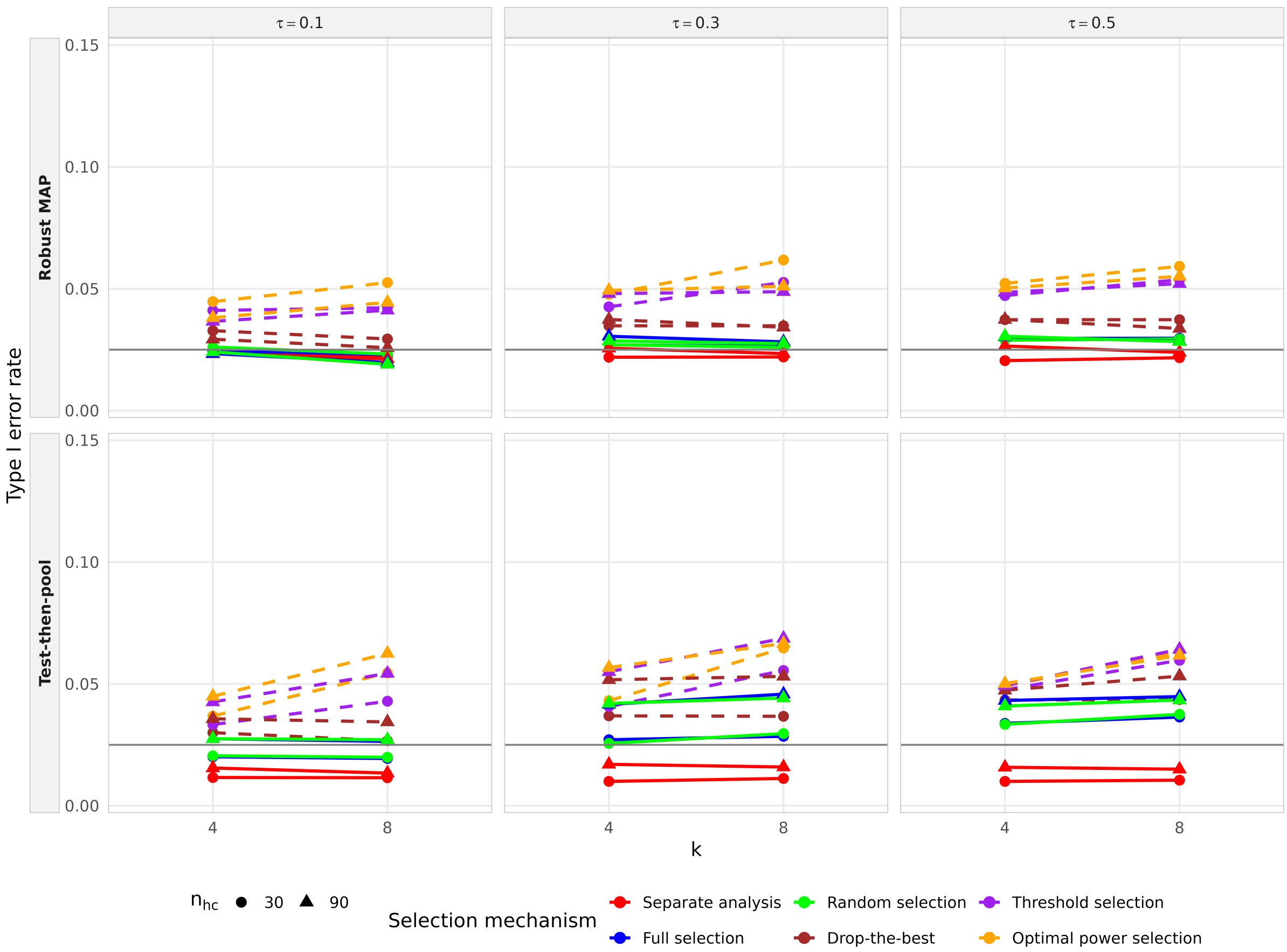

Supplementary Figure S2: Type I error rates of various selection mechanisms, to show the impact of numbers of historical trials k (x-axis) and sample size per historical control arm n_{hc} (circle: 30, triangle: 90); Type I error rates of different levels of between-trial heterogeneity τ are provided, with fixed 1:1 randomization ratio. Dashed line: outcome-dependent selections. Results from both robust MAP and TTP are provided.

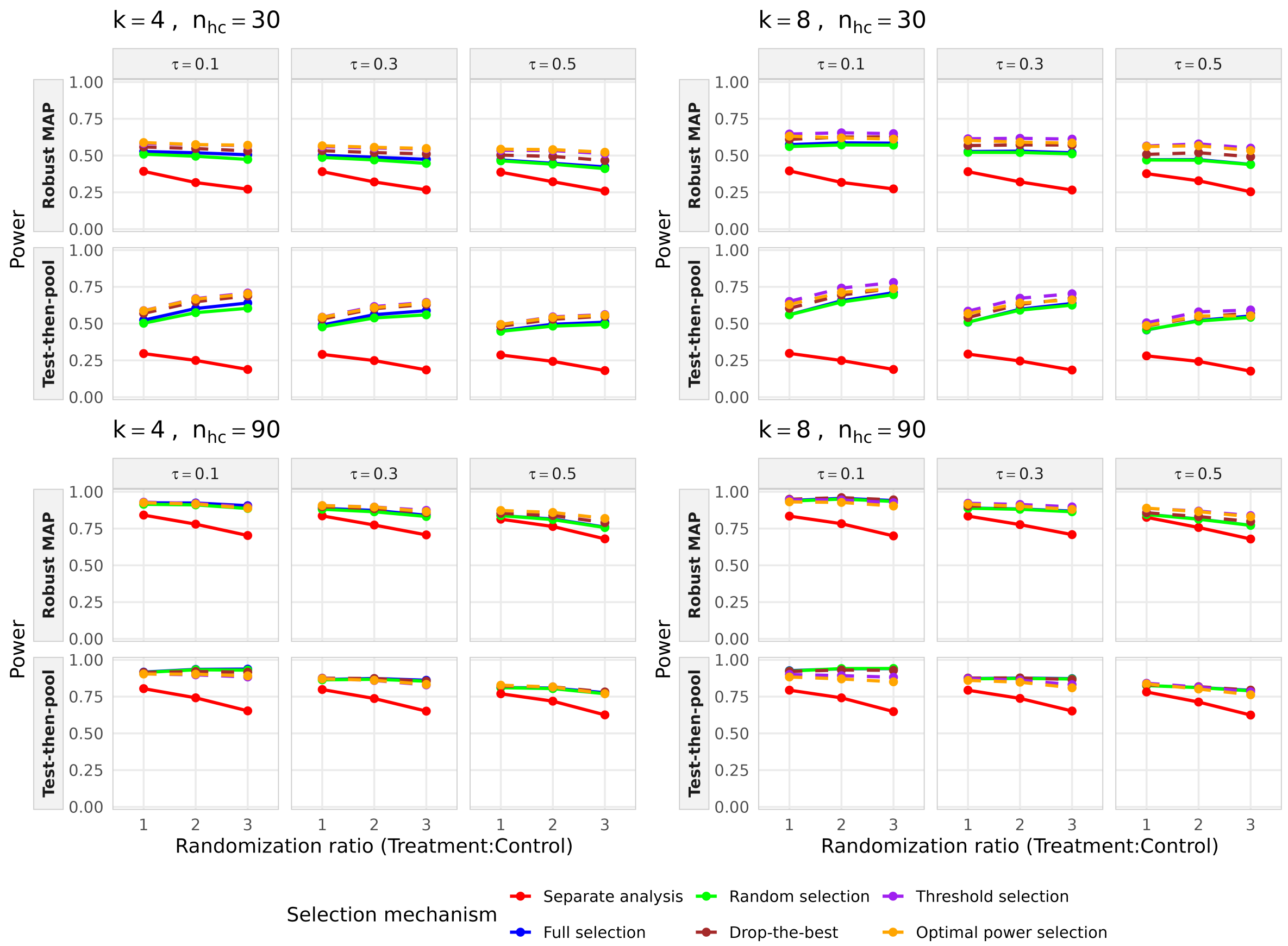

Supplementary Figure S3: Power of various selection mechanisms, to show the impact of randomization ratio (x-axis, total sample size in the prospective trial is fixed at $n_{total} = 2 * n_{hc}$) and between-trial heterogeneity τ (columns); Power of different numbers of historical trials k and sample size per historical control arm n_{hc} are provided. Dashed line: outcome-dependent selections. Results from both robust MAP and TTP are provided.

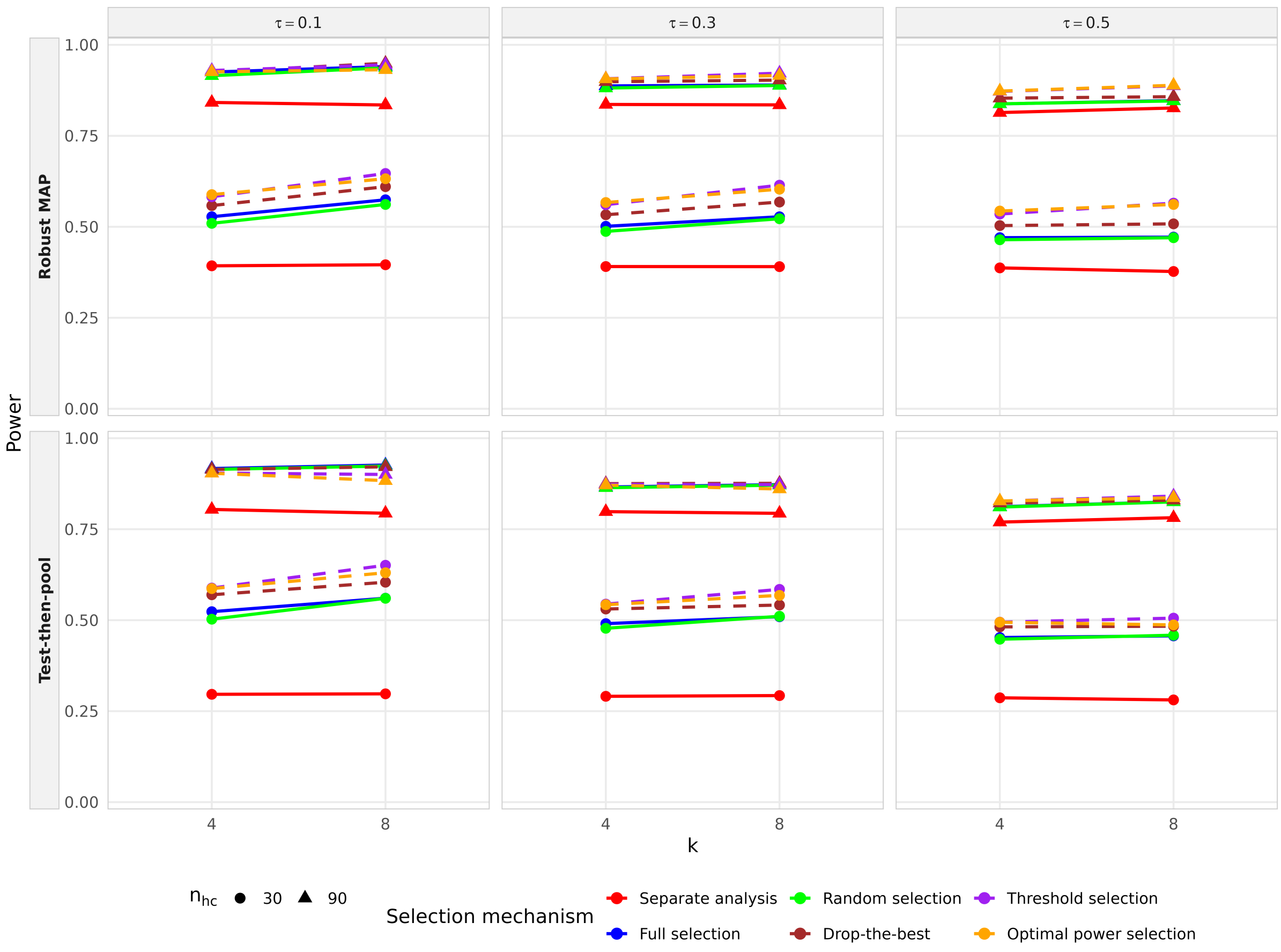

Supplementary Figure S4: Power of various selection mechanisms, to show the impact of numbers of historical trials k (x-axis) and sample size per historical control arm n_{hc} (circle: 30, triangle: 90); Power of different levels of between-trial heterogeneity τ are provided, with fixed 1:1 randomization ratio. Dashed line: outcome-dependent selections. Results from both robust MAP and TTP are provided.

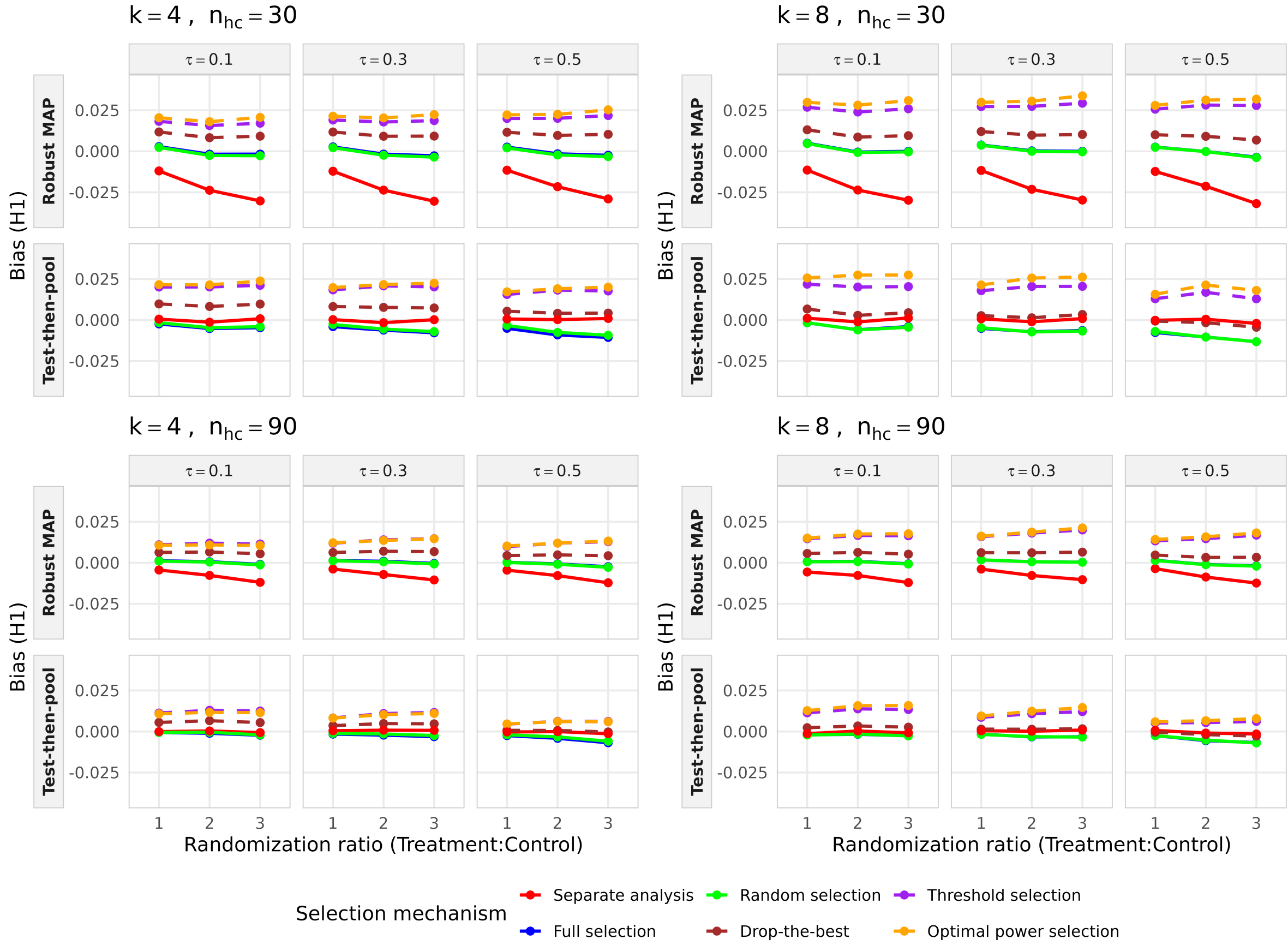

Supplementary Figure S5: Bias of various selection mechanisms under H_1, to show the impact of randomization ratio (x-axis, total sample size in the prospective trial is fixed at $n_{total} = 2 * n_{hc}$) and between-trial heterogeneity τ (columns); Bias of different numbers of historical trials k and sample size per historical control arm n_{hc} are provided. Dashed line: outcome-dependent selections. Results from both robust MAP and TTP are provided.

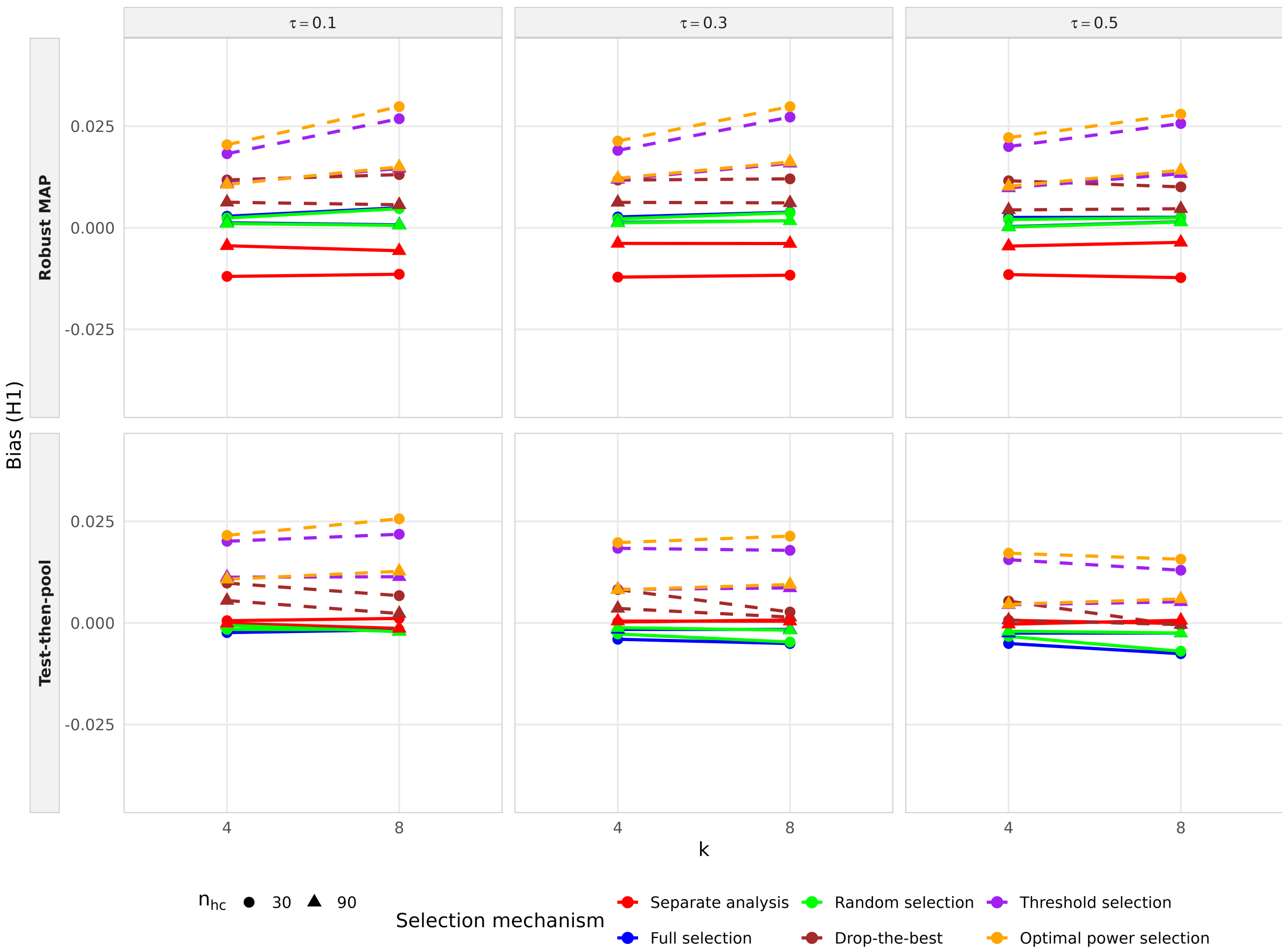

Supplementary Figure S6: Bias of various selection mechanisms under H_1, to show the impact of numbers of historical trials k (x-axis) and sample size per historical control arm n_{hc} (circle: 30, triangle: 90); Bias of different levels of between-trial heterogeneity τ are provided, with fixed 1:1 randomization ratio. Dashed line: outcome-dependent selections. Results from both robust MAP and TTP are provided.

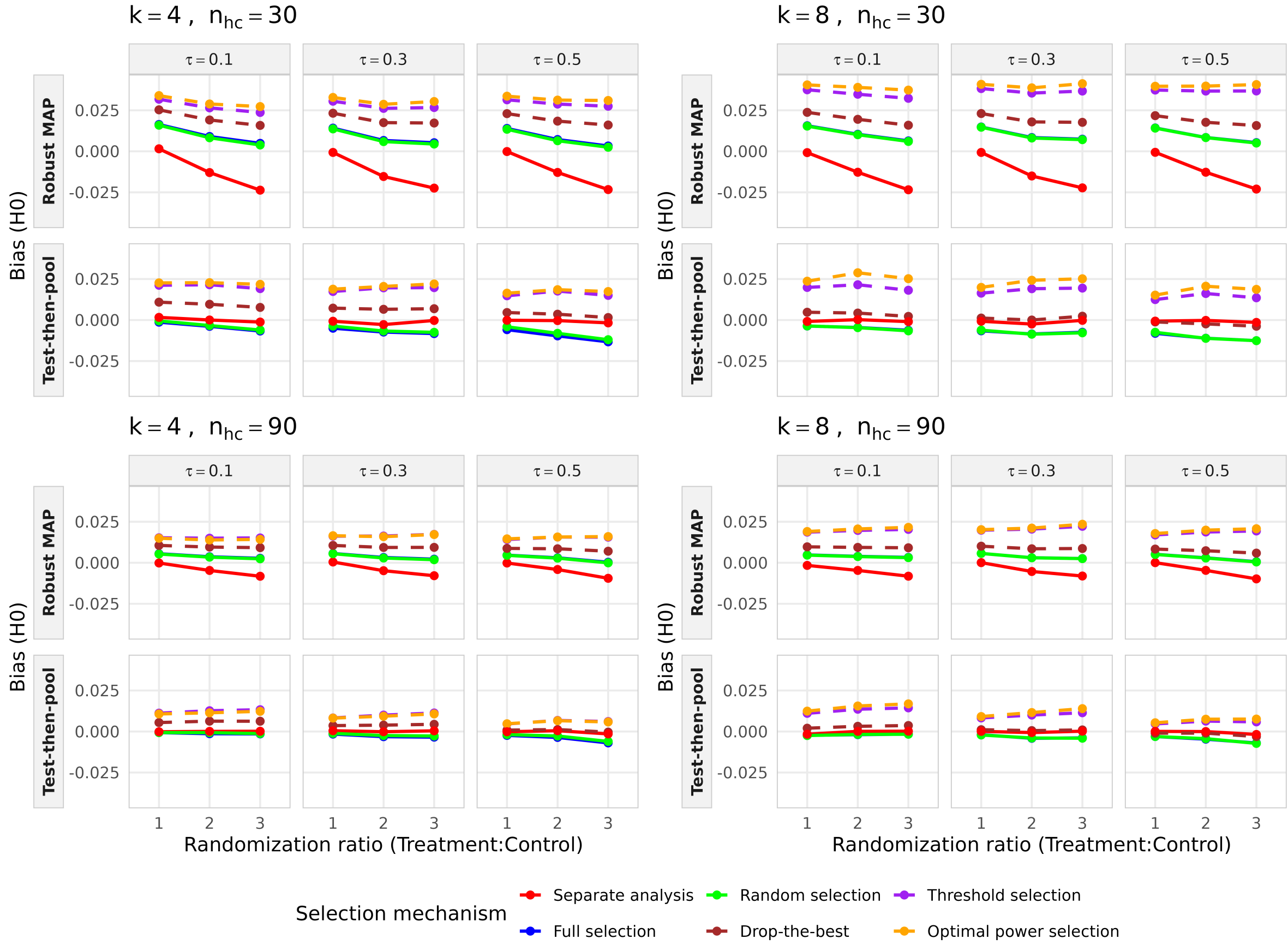

Supplementary Figure S7: Bias of various selection mechanisms under H_0, to show the impact of randomization ratio (x-axis, total sample size in the prospective trial is fixed at $n_{total} = 2 * n_{hc}$) and between-trial heterogeneity τ (columns); Bias of different numbers of historical trials k and sample size per historical control arm n_{hc} are provided. Dashed line: outcome-dependent selections. Results from both robust MAP and TTP are provided.

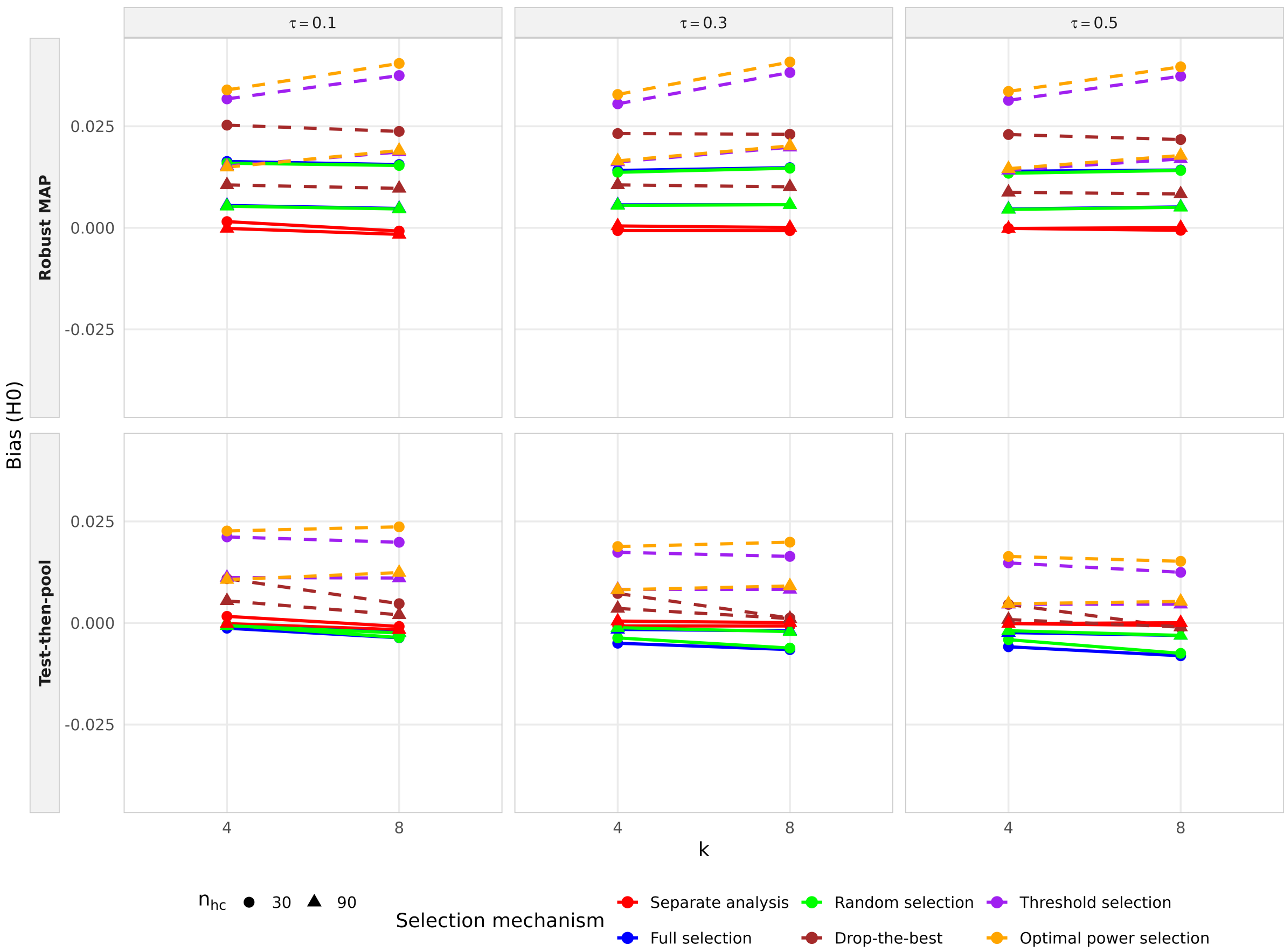

Supplementary Figure S8: Bias of various selection mechanisms under H_0, to show the impact of numbers of historical trials k (x-axis) and sample size per historical control arm n_{hc} (circle: 30, triangle: 90); Bias of different levels of between-trial heterogeneity τ are provided, with fixed 1:1 randomization ratio. Dashed line: outcome-dependent selections. Results from both robust MAP and TTP are provided.

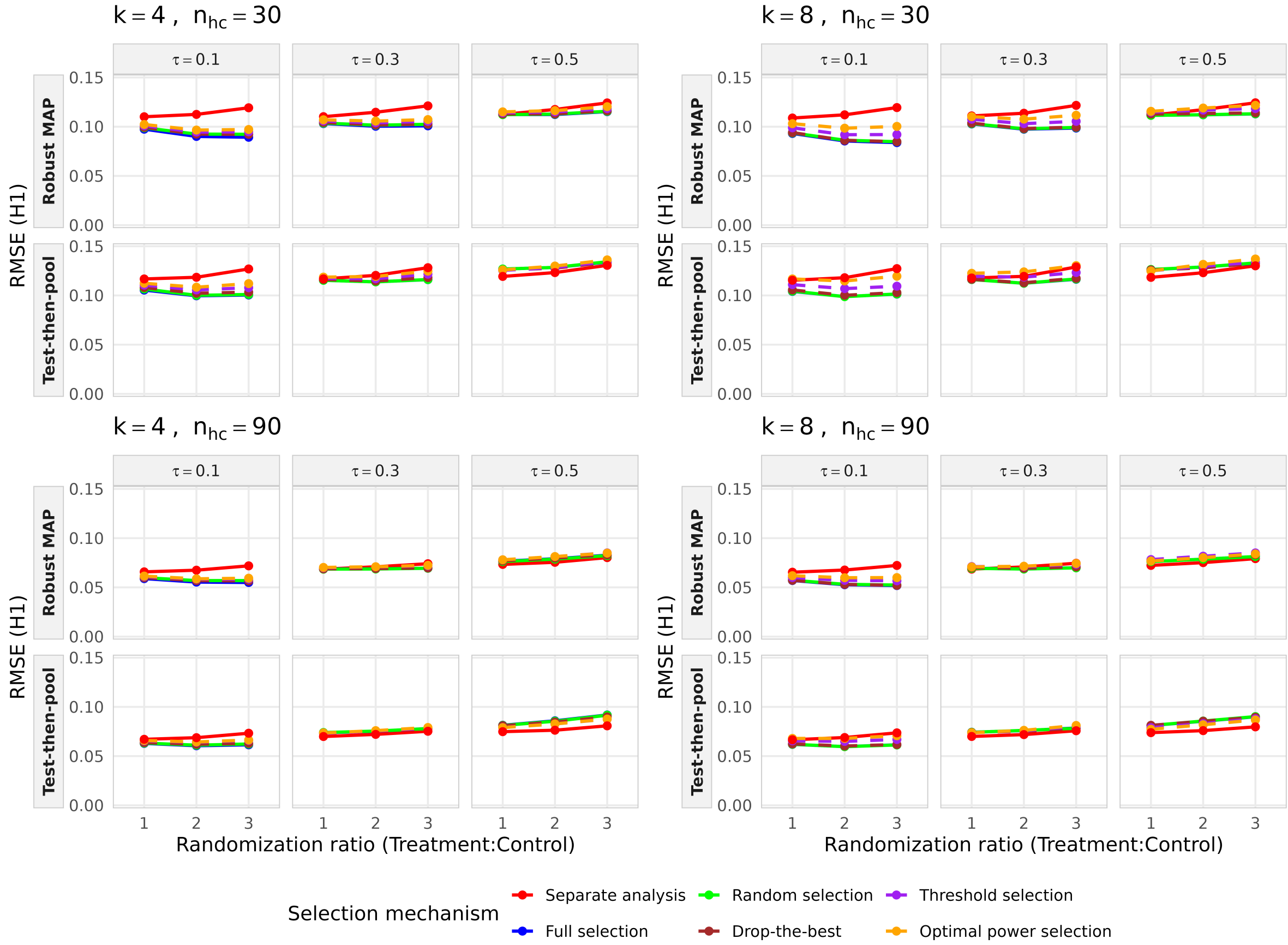

Supplementary Figure S9: RMSE of various selection mechanisms under H_1, to show the impact of randomization ratio (x-axis, total sample size in the prospective trial is fixed at $n_{total} = 2*n_{hc}$) and between-trial heterogeneity τ (columns); RMSE of different numbers of historical trials k and sample size per historical control arm n_{hc} are provided. Dashed line: outcome-dependent selections. Results from both robust MAP and TTP are provided.

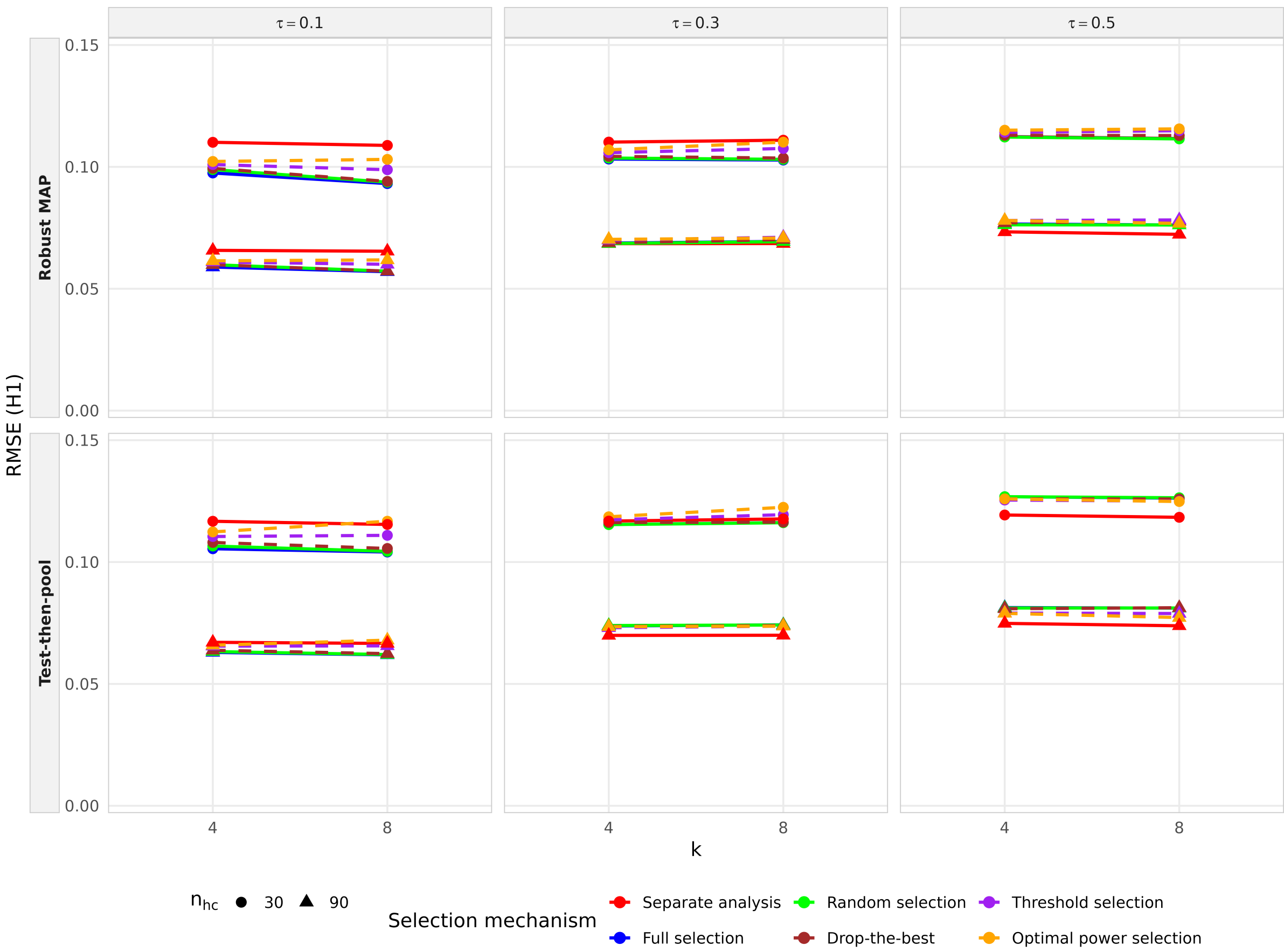

Supplementary Figure S10: RMSE of various selection mechanisms under H_1, to show the impact of numbers of historical trials k (x-axis) and sample size per historical control arm n_{hc} (circle: 30, triangle: 90); RMSE of different levels of between-trial heterogeneity τ are provided, with fixed 1:1 randomization ratio. Dashed line: outcome-dependent selections. Results from both robust MAP and TTP are provided.

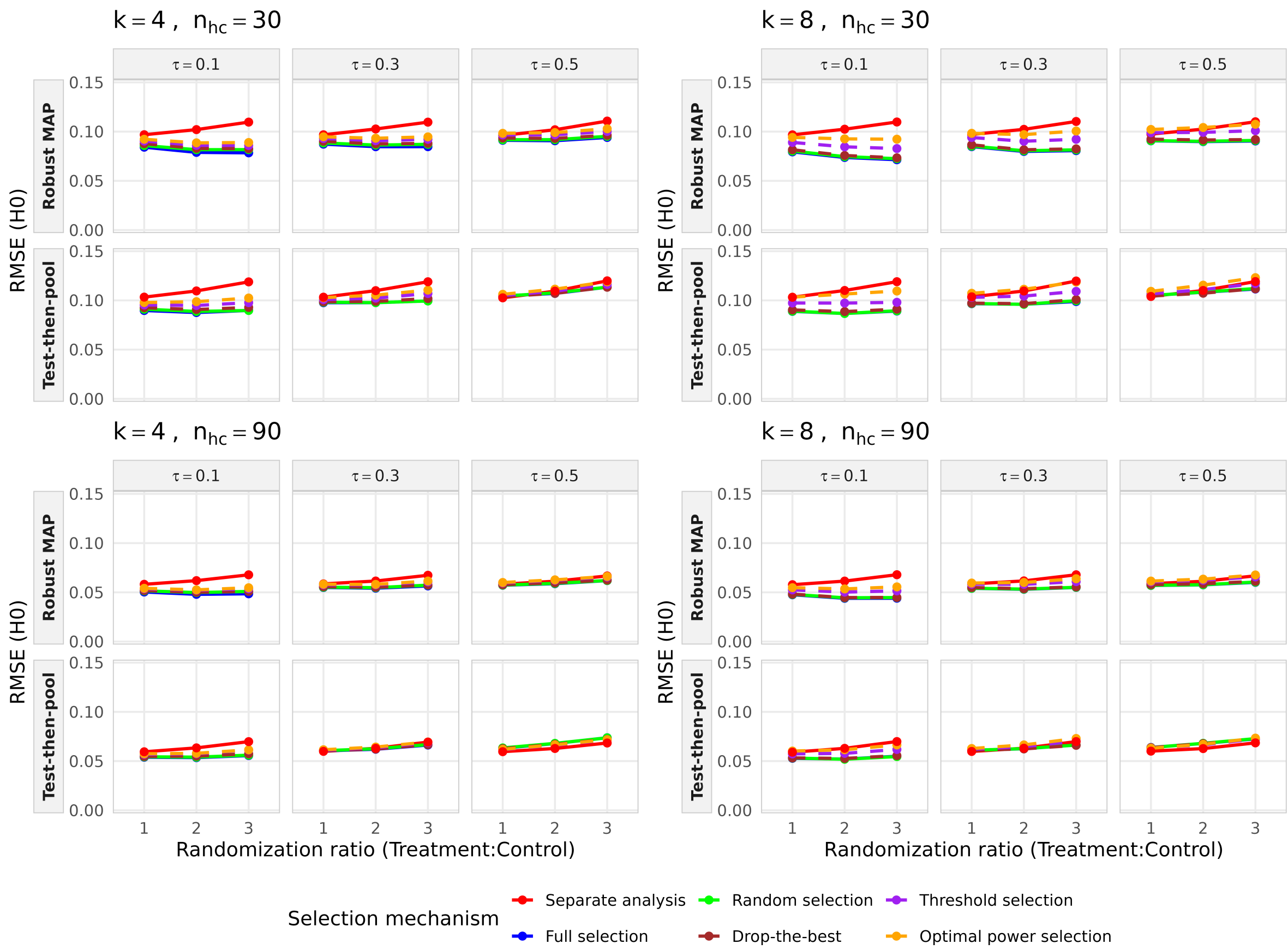

Supplementary Figure S11: RMSE of various selection mechanisms under H_0, to show the impact of randomization ratio (x-axis, total sample size in the prospective trial is fixed at $n_{total} = 2 * n_{hc}$) and between-trial heterogeneity τ (columns); RMSE of different numbers of historical trials k and sample size per historical control arm n_{hc} are provided. Dashed line: outcome-dependent selections. Results from both robust MAP and TTP are provided.

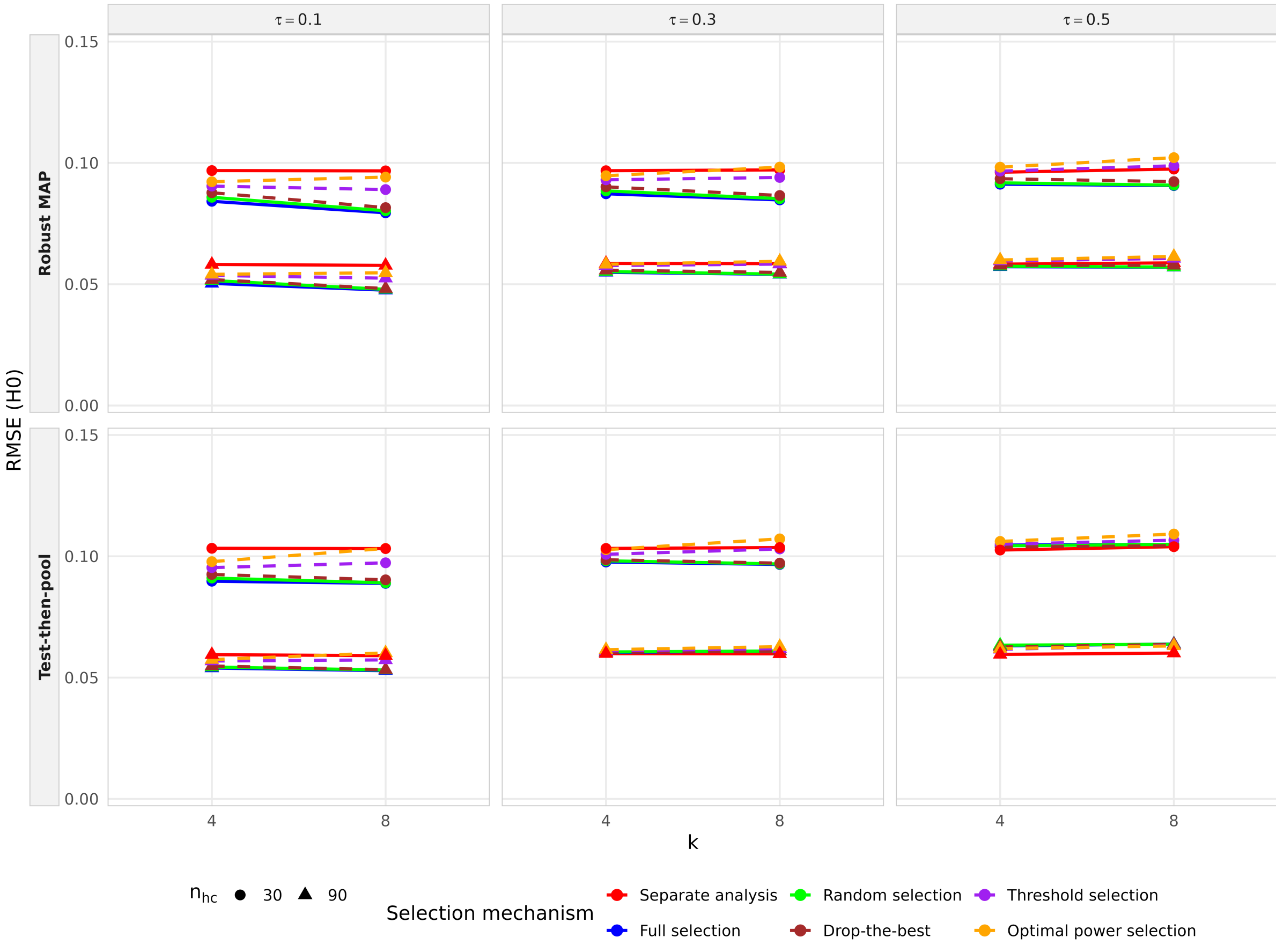

Supplementary Figure S12: RMSE of various selection mechanisms under H_0, to show the impact of numbers of historical trials k (x-axis) and sample size per historical control arm n_{hc} (circle: 30, triangle: 90); RMSE of different levels of between-trial heterogeneity τ are provided, with fixed 1:1 randomization ratio. Dashed line: outcome-dependent selections. Results from both robust MAP and TTP are provided.

Supplementary Table S1: Effective Sample Size (ESS) of robust MAP prior across selection mechanisms (excluding optimal power selection), half-normal prior for between-trial heterogeneity $\tau \sim HN(1)$ as default. k: numbers of historical trials; n_{hc}: sample size per historical control arm. Note that the total sample size in the prospective trial is fixed at $n_{total} = 2 * n_{hc}$.

τ	k	n_{hc}	Full selection	Random selection	Drop-the-best	Threshold selection
0.10	4	30	23.52	16.75	18.76	19.23
0.10	4	90	60.62	39.78	45.54	46.88
0.10	8	30	51.00	44.45	52.27	39.85
0.10	8	90	141.52	122.15	147.01	99.31
0.30	4	30	20.79	15.33	17.85	18.62
0.30	4	90	41.33	30.17	37.80	43.88
0.30	8	30	40.15	35.68	44.76	36.19
0.30	8	90	71.05	65.69	93.08	80.88
0.50	4	30	16.81	13.35	16.42	18.20
0.50	4	90	25.31	21.39	29.63	40.79
0.50	8	30	26.12	24.29	34.18	32.41
0.50	8	90	28.30	28.60	46.50	63.11

Supplementary Table S2: Effective Sample Size (ESS) across selection mechanisms (excluding optimal power selection), half-normal prior between-trial heterogeneity for $\tau \sim HN(0.5)$ as sensitivity analysis. k: numbers of historical trials; n_{hc}: sample size per historical control arm. Note that the total sample size in the prospective trial is fixed at $n_{total} = 2 * n_{hc}$.

τ	k	n_{hc}	Full selection	Random selection	Drop-the-best	Threshold selection
0.10	4	30	32.73	25.66	28.41	27.19
0.10	4	90	74.96	54.78	61.54	59.40
0.10	8	30	60.03	53.28	61.61	50.00
0.10	8	90	151.97	133.34	158.35	113.65
0.30	4	30	29.80	24.00	27.56	26.57
0.30	4	90	53.61	43.38	53.15	56.04
0.30	8	30	48.92	44.32	54.44	46.91
0.30	8	90	79.59	74.98	104.22	96.33
0.50	4	30	25.12	21.48	26.07	26.26
0.50	4	90	34.55	32.07	43.50	52.71
0.50	8	30	33.65	31.88	43.77	43.98
0.50	8	90	33.49	34.57	54.89	79.13

Supplementary Table S3: Effective Sample Size (ESS) for optimal power selection by randomization ratio, half-normal prior for between-trial heterogeneity $\tau \sim HN(1)$ as default. k: numbers of historical trials; n_{hc}: sample size per historical control arm. In contrast to the other selection mechanisms, optimal power selection depends on the design specification of the prospective trial, such as allocation ratio. Note that the total sample size in the prospective trial is fixed at $n_{total} = 2 * n_{hc}$.

τ	k	n_{hc}	Ratio	ESS
0.10	4	30	1	19.09
0.10	4	30	2	18.20
0.10	4	30	3	18.35
0.10	4	90	1	41.27
0.10	4	90	2	42.19
0.10	4	90	3	40.35
0.10	8	30	1	28.10
0.10	8	30	2	25.85
0.10	8	30	3	24.94
0.10	8	90	1	63.50
0.10	8	90	2	65.14
0.10	8	90	3	66.18
0.30	4	30	1	19.01
0.30	4	30	2	18.22
0.30	4	30	3	18.52
0.30	4	90	1	41.91
0.30	4	90	2	40.69
0.30	4	90	3	39.56
0.30	8	30	1	26.68
0.30	8	30	2	24.75
0.30	8	30	3	23.99
0.30	8	90	1	55.03
0.30	8	90	2	55.05
0.30	8	90	3	54.98
0.50	4	30	1	19.11
0.50	4	30	2	18.55
0.50	4	30	3	18.80
0.50	4	90	1	42.36
0.50	4	90	2	40.82
0.50	4	90	3	40.38
0.50	8	30	1	25.53
0.50	8	30	2	24.12
0.50	8	30	3	23.46
0.50	8	90	1	49.13
0.50	8	90	2	48.51
0.50	8	90	3	47.92

Supplementary Table S4: Effective Sample Size (ESS) for optimal power selection by randomization ratio, under the alternative hypothesis; half-normal prior for between-trial heterogeneity $\tau \sim HN(0.5)$ as sensitivity analysis. k: numbers of historical trials; n_{hc}: sample size per historical control arm. In contrast to the other selection mechanisms, optimal power selection depends on the design specification of the prospective trial, such as allocation ratio. Note that the total sample size in the prospective trial is fixed at $n_{total} = 2 * n_{hc}$.

τ	k	n_{hc}	Ratio	ESS
0.10	4	30	1	26.54
0.10	4	30	2	25.88
0.10	4	30	3	25.72
0.10	4	90	1	55.37
0.10	4	90	2	56.91
0.10	4	90	3	55.19
0.10	8	30	1	38.13
0.10	8	30	2	35.75
0.10	8	30	3	34.88
0.10	8	90	1	79.57
0.10	8	90	2	81.29
0.10	8	90	3	82.12
0.30	4	30	1	26.32
0.30	4	30	2	25.75
0.30	4	30	3	25.75
0.30	4	90	1	54.97
0.30	4	90	2	54.54
0.30	4	90	3	53.47
0.30	8	30	1	37.06
0.30	8	30	2	35.00
0.30	8	30	3	34.27
0.30	8	90	1	71.81
0.30	8	90	2	72.04
0.30	8	90	3	71.83
0.50	4	30	1	26.41
0.50	4	30	2	26.00
0.50	4	30	3	26.04
0.50	4	90	1	54.80
0.50	4	90	2	53.89
0.50	4	90	3	53.50
0.50	8	30	1	36.65
0.50	8	30	2	35.08
0.50	8	30	3	34.45
0.50	8	90	1	66.89
0.50	8	90	2	66.46
0.50	8	90	3	65.79

Monotone selection Monotone selection is a stricter selection mechanism introduced in Section 5 of the main text as a possible way to mitigate outcome-dependent selection bias. The performance measures of monotone selection implemented on optimal power selected trial are shown in Supplementary Table S5 and Supplementary Figure S13–S14. For the ease of interpretation only results of $k = 8$ historical trials, with sample size per historical control arm $n_{hc} = 30$, and moderate between-trial heterogeneity $\tau = 0.30$ are provided.

Supplementary Table S5: As Table 5 ($k = 8$ historical trials, with sample size per historical control arm $n_{hc} = 30$, and moderate between-trial heterogeneity $\tau = 0.30$), with an additional row reporting the monotone selection constraint applied to the optimal power selection. *Monotone selection* (red): if a later trial is excluded, all earlier trials are also excluded.

**Selection mechanisms**	**Exchangeable**				**Time-trend**				**Large prospective trial**			
	T1E	Power	Bias	RMSE	T1E	Power	Bias	RMSE	T1E	Power	Bias	RMSE
Separate analysis	2.20	39.05	−1.17	11.09	2.20	39.05	−1.17	11.09	2.57	38.87	−0.04	3.85
Full selection	2.63	52.75	0.38	10.28	3.95	58.32	1.85	10.52	2.83	42.91	0.18	3.90
Random selection	2.60	52.21	0.37	10.31	3.95	57.70	1.78	10.52	2.79	42.62	0.17	3.89
Drop-the-best	3.48	56.80	1.21	10.36	5.27	61.92	2.62	10.73	3.28	45.98	0.41	3.92
Threshold selection	5.26	61.43	2.73	10.76	6.54	63.96	3.38	11.01	4.29	49.38	0.80	3.95
Optimal power selection	6.18	60.33	2.98	11.02	7.33	60.67	3.47	11.34	4.35	49.03	0.79	3.95
Monotone selection	3.63	48.02	0.43	10.95	3.62	45.97	0.15	11.06	3.09	42.95	0.30	3.88

Exchangeable: reference risk difference $RD = 0.20$.
Time-trend: linear drift in the historical data.
Large prospective trial: prospective trial with $RD = 0.0635$ with $n = 250$ per arm.
RD: treatment effect measure, risk differences between the treatment and control arm.
All values are **percentages**. T1E: type I error rate at nominal one-sided level 2.5%. Bias: percentage-point deviation of posterior mean RD from the truth, under H_1, calculated by $\frac{1}{n_{\text{sim}}}\sum_{r=1}^{n_{\text{sim}}}\big(\hat{RD}^{(r)} - RD\big)\times 100$. RMSE: root mean square error, under H_1, calculated by $\sqrt{\frac{1}{n_{\text{sim}}}\sum_{r=1}^{n_{\text{sim}}}\big(\hat{RD}^{(r)} - RD\big)^2}\times 100$..

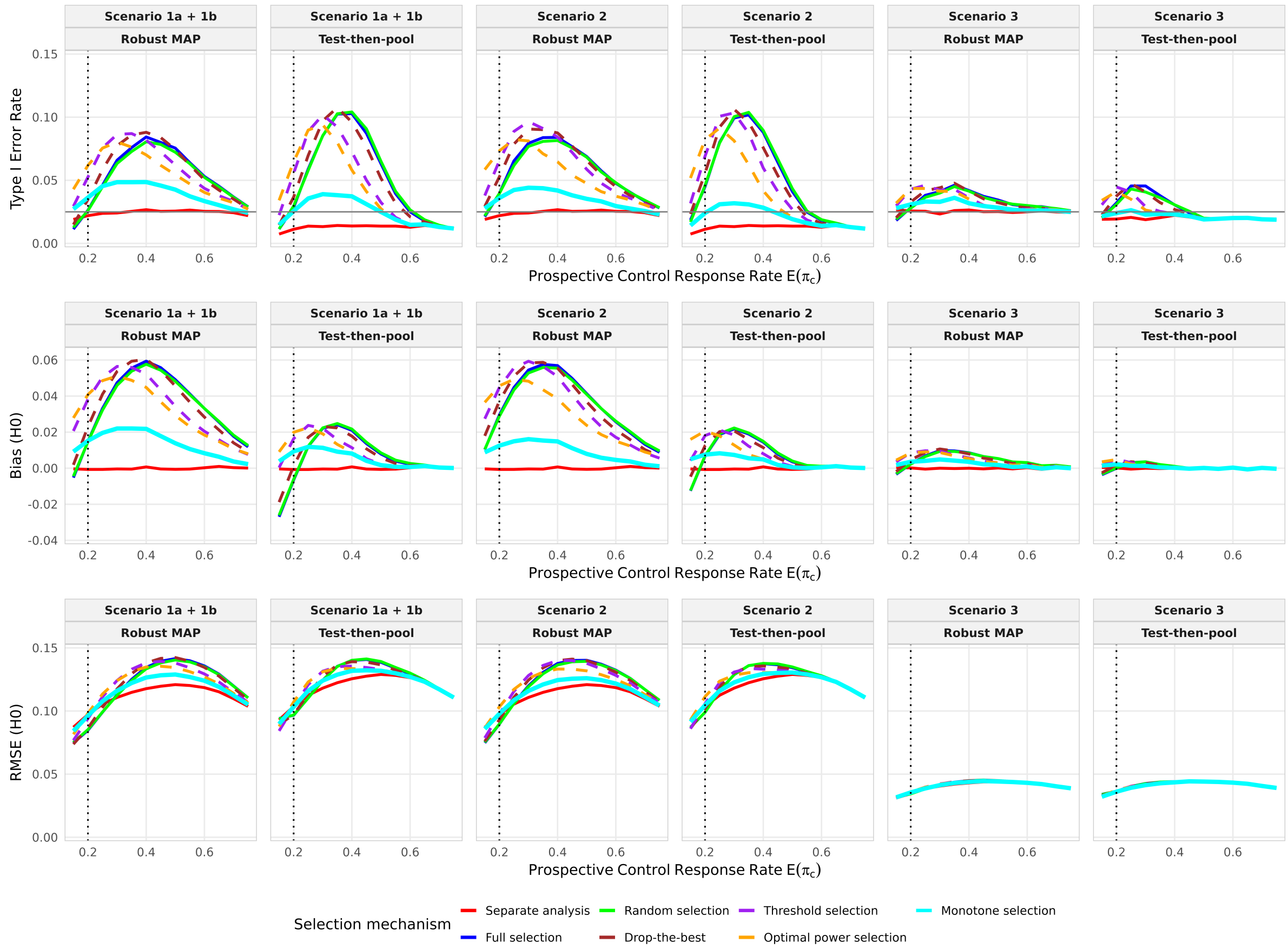

Supplementary Figure S13: Performance under the null hypothesis with $k = 8$ historical trials, with sample size per historical control arm $n_{hc} = 30$, and moderate heterogeneity between historical trials ($\tau = 0.30$). Scenario 1a + 1b : under H_0, $RD = 0$. Scenario 2 Time-trend: linear drift in historical data. Scenario 3 Large prospective trial: under H_0, $RD = 0$ and with $n = 250$ per arm. Panels show Type I error rate, Bias (H0), and RMSE (H0). x-axis: Prospective control response rate; the vertical line at $x = 0.20$ indicates exchangeability (Scenario 1a). Dashed line: outcome-dependent selections. Cyan solid line: Monotone selection, mitigating the impact of optimal power selection (orange dashed line).

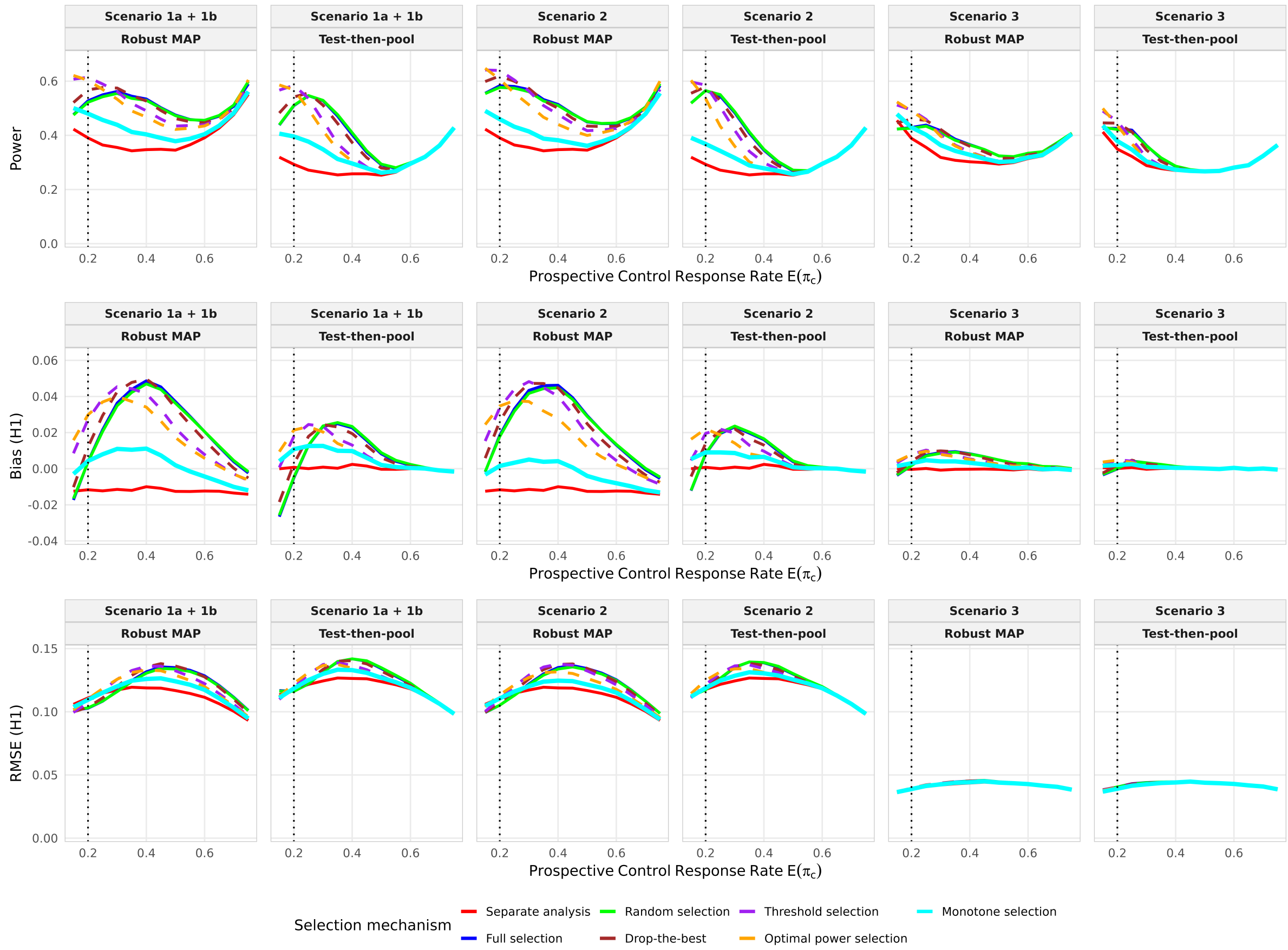

Supplementary Figure S14: Performance under the alternative hypothesis with $k = 8$ historical trials, with sample size per historical control arm $n_{hc} = 30$, and moderate heterogeneity between historical trials ($\tau = 0.30$). Scenario 1a + 1b : reference risk difference $RD = 0.20$. Scenario 2 Time-trend: linear drift in historical data. Scenario 3 Large prospective trial: $RD = 0.0635$ with $n = 250$ per arm. Panels show Power, Bias (H1), and RMSE (H1). x-axis: Prospective control response rate; the vertical line at $x = 0.20$ indicates exchangeability (Scenario 1a). Dashed line: outcome-dependent selections. Cyan solid line: Monotone selection, mitigating the impact of optimal power selection (orange dashed line).

S3 Additional information for case study

In this section we present additional information for the case study in the ankylosing spondylitis (AS) trial data originally from Baeten et al.[5]. In the main manuscript the case study is described in more detail in Section 4. Supplementary Figure S15 shows which of the 8 historical studies have been selected depending on the selection mechanism. Supplementary Table S6 shows the results from applying the frequentist TTP analysis to the selected trials (see methods in main text Section 2). The two-sided pre-test at significance level 0.1 was implemented using Fisher's exact test. In the second step a one-sided Fisher's exact test at $\alpha = 0.025$ was used to compare the treatment and control. The estimates and 95% confidence intervals of risk differences are obtained using the two-sample test for equality of proportions with Yates continuity correction (large-sample chi-square test, `prop.test()` function), as the Fisher's exact test only provides odds ratio. In Supplementary Figure S16 additional conditional results based on the same historical studies with varying prospective settings are presented.

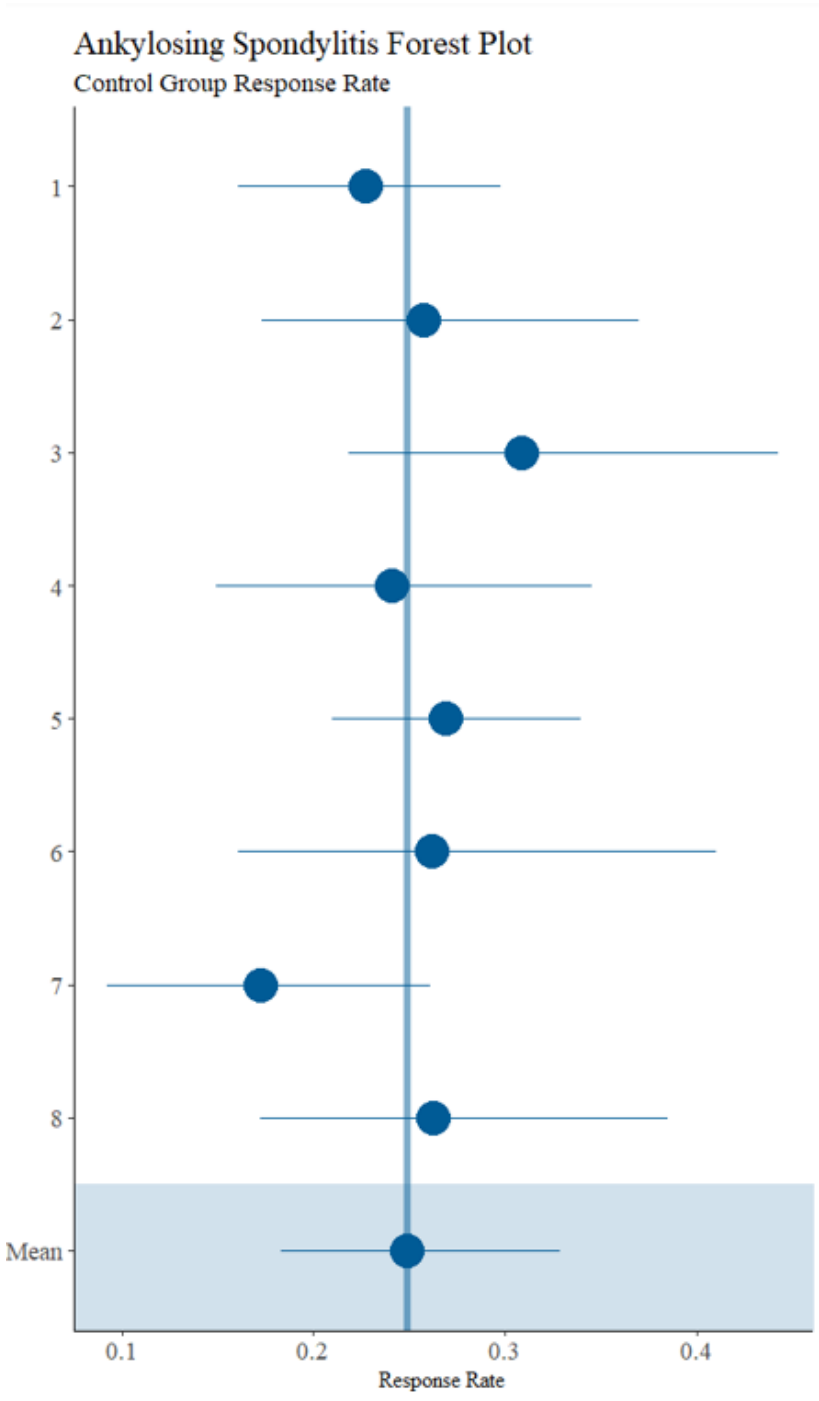

Full selection	Random selection	Drop-the-best	Threshold selection	Optimal Power selection	Separate analysis
✓		✓	✓		
✓	✓	✓			
✓	✓				
✓	✓	✓	✓		
✓	✓	✓			
✓	✓	✓			
✓	✓	✓	✓	✓	
✓	✓	✓			
The mean of these 8 AS trials ASAS20 response rates is 0.25, served as the threshold in threshold selection of this case study.					

Supplementary Figure S15: Selection results in the case study. Left: Forest plot of AS data, centered at 0.25; Right: selected trials with indicators (✓)

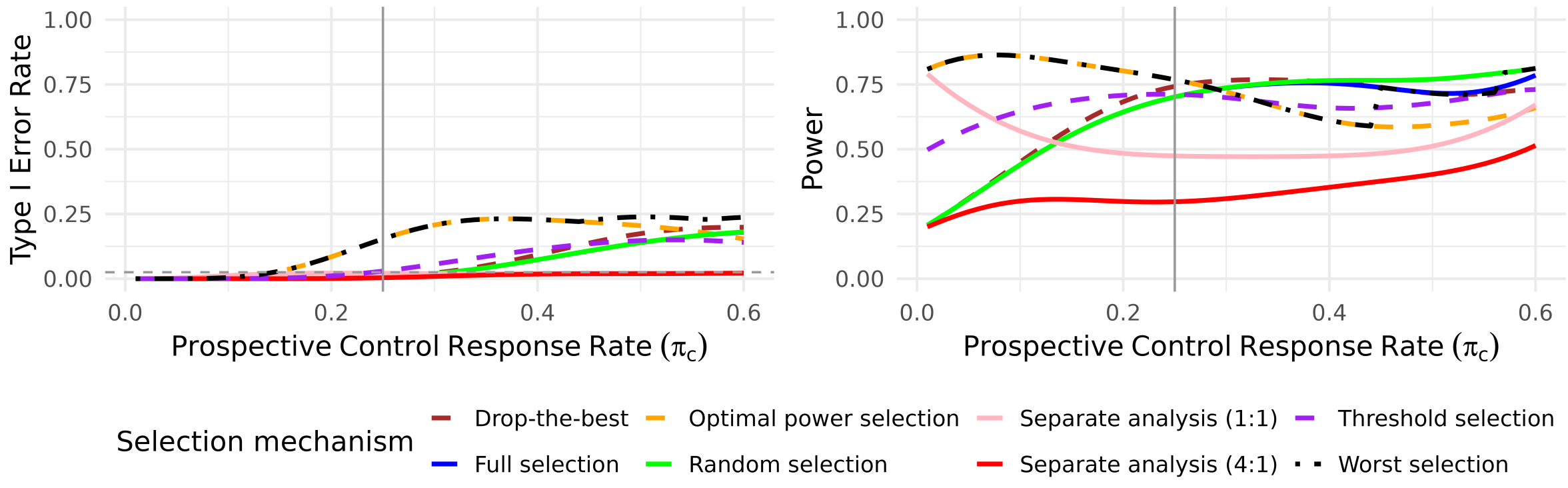

(a) Conditional type I error and conditional power for $n_{\text{total}} = 30$.

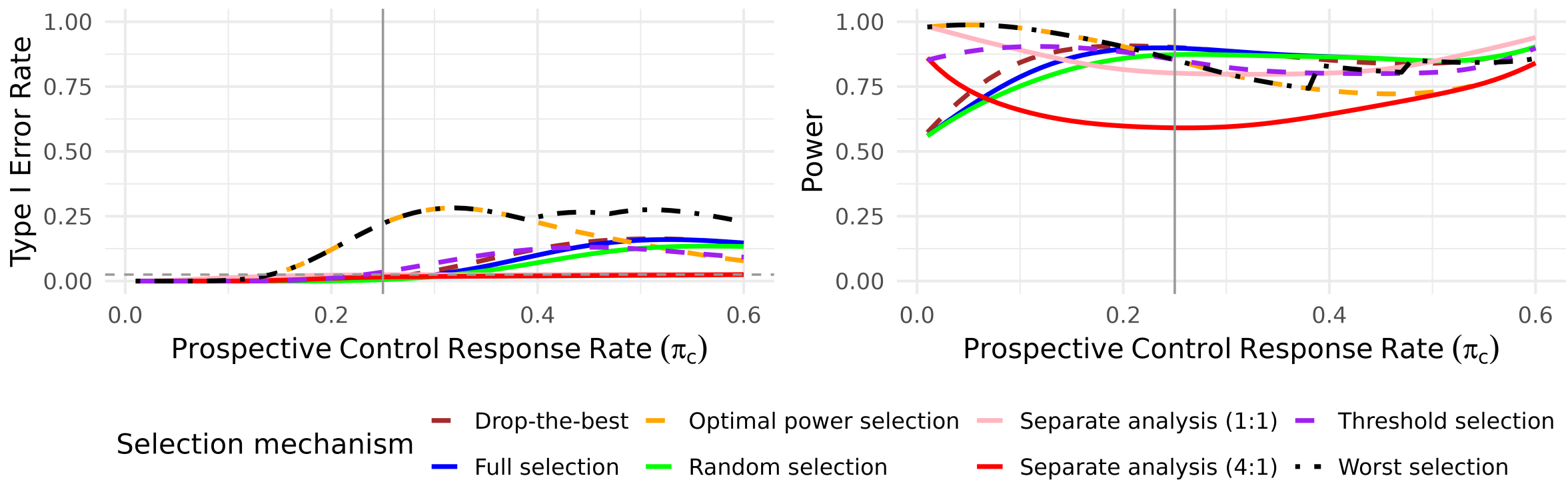

(b) Conditional type I error and conditional power for $n_{\text{total}} = 60$.

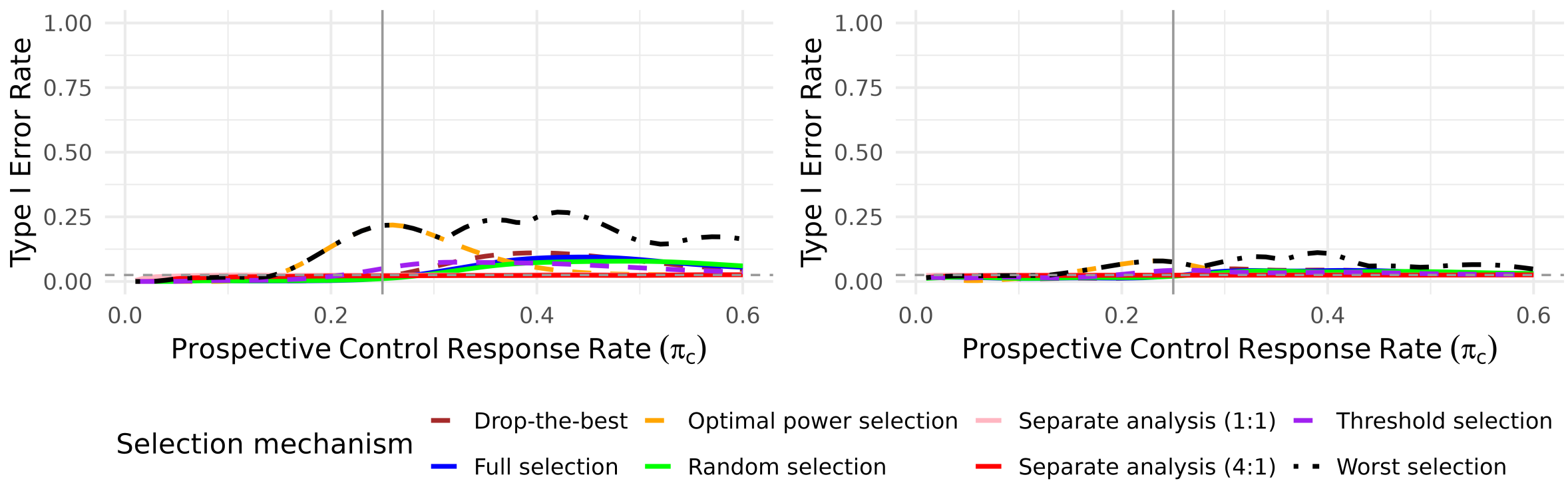

(c) Conditional type I error for $n_{\text{total}} = 300, 3000$.

Supplementary Figure S16: Conditional operating characteristics of different selection mechanisms based on the same historical pool and varying prospective settings. (a) Conditional type I error rates and power when prospective trial planned to have total sample size $n_{\text{total}} = 30$. (b) Conditional type I error rates and power when prospective trial planned to have total sample size $n_{\text{total}} = 60$. (c) Conditional type I error rates when prospective trial planned to have total sample size $n_{\text{total}} = 300$ (left) and $n_{\text{total}} = 3000$ (right), power is not shown here as under this sample size the power will be very close to 1. The black dashed line marks the worst (max conditional type I error) selection.

Supplementary Table S6: Results of applying various selection mechanisms to the ankylosing spondylitis (AS) case study trial data, analyzed with TTP.

**Selection mechanism**	**Estimate**	**95% CI**	***p*-value**
Full selection	0.337	(0.114, 0.559)	< 0.001
Random selection	0.328	(0.105, 0.552)	< 0.001
Drop-the-best	0.350	(0.128, 0.573)	< 0.001
Threshold selection	0.401	(0.174, 0.627)	< 0.001
Optimal Power selection	0.464	(0.228, 0.700)	< 0.001
Separate analysis	0.417	(-0.045, 0.878)	0.084

Estimate: Risk difference between treatment and control from two-sample test for equality of proportions with continuity correction. 95% CI: confidence interval from two-sample test for equality of proportions with continuity correction. p-value: Obtained from Fisher's exact test with one-sided α=0.025

S4 Design-stage quantities (binary endpoint; Beta(1,1) for treatment; robust MAP for control)

When planning a hybrid RCT the observed rates of the historical control data are already available. Therefore, one can already condition on the observed results when calculating operating characteristics such as conditional type I error rates or power. The following section derives how these quantities can be calculated using the binomial likelihood. These formulas were used to calculate the conditional operating characteristics for the case study presented in Section 4.1 in the main manuscript.

Let $\pi_t^\star, \pi_c^\star \in (0,1)$ denote the *assumed true* response rates for design evaluation, n_t, n_c the planned sample sizes, and γ the posterior success threshold (e.g. $\gamma = 0.975$). The treatment prior is $\pi_t \sim \text{Beta}(1,1)$ and the control prior is a robust MAP derived from the selected historical trials $\mathcal{H}$

$$\pi_c \sim w_R \,\text{Beta}(1,1) + (1-w_R)\sum_{\ell=1}^{K} w_\ell \,\text{Beta}(a_\ell, b_\ell), \qquad w_R \in (0,1),\ w_\ell > 0,\ \sum_{\ell=1}^{K} w_\ell = 1.$$

Decision rule and boundary. Given counts (y_t, y_c), the posterior probability of superiority decomposes as

$$\Pr(\pi_t > \pi_c \mid \mathcal{D}_0, \mathcal{H}) = \ \widetilde{w}_R(y_c)\ \Pr\big[X > Y_0\big] \ + \ \big(1 - \widetilde{w}_R(y_c)\big)\sum_{\ell=1}^{K} \widetilde{w}_\ell(y_c)\ \Pr\big[X > Y_\ell\big],$$

where $\mathcal{D}_0 = (y_t, y_c;\, n_t, n_c)$ denotes the prospective trial data, and

$$X \sim \text{Beta}(\underbrace{1+y_t}_{\alpha_1}, \underbrace{1+n_t-y_t}_{\beta_1}), \quad Y_0 \sim \text{Beta}(\underbrace{1+y_c}_{\gamma_0}, \underbrace{1+n_c-y_c}_{\delta_0}), \quad Y_\ell \sim \text{Beta}(\underbrace{a_\ell+y_c}_{\gamma_\ell}, \underbrace{b_\ell+n_c-y_c}_{\delta_\ell}).$$

Each term $\Pr[X > Y_\bullet]$ has the closed-form series [2] when $\alpha_1 \in \mathbb{N}$:

$$\Pr[X > Y] \ = \ \sum_{s=0}^{\alpha_1 - 1} \frac{B(\gamma + s,\ \beta_1 + \delta)}{(\beta_1 + s) B(\gamma, \delta)\, B(s+1, \beta_1)}.$$

The updated mixture weights are based on component-wise marginal likelihoods

$$m_0(y_c) \propto \frac{B(1+y_c,\ 1+n_c-y_c)}{B(1,1)}, \qquad m_\ell(y_c) \propto \frac{B(a_\ell+y_c,\ b_\ell+n_c-y_c)}{B(a_\ell, b_\ell)},$$

$$\widetilde{w}_R(y_c) = \frac{w_R\, m_0(y_c)}{w_R\, m_0(y_c) + (1-w_R)\sum_{j=1}^{K} w_j\, m_j(y_c)}, \qquad \widetilde{w}_\ell(y_c) = \frac{w_\ell\, m_\ell(y_c)}{\sum_{j=1}^{K} w_j\, m_j(y_c)}.$$

Define the data-dependent boundary as the largest treatment count not crossing the threshold,

$$d_1(y_c) = \max\{\, y_t \le n_t :\ \Pr(\pi_t > \pi_c \mid \mathcal{D}_0, \mathcal{H}) \le \gamma \,\}. \tag{S4.5}$$

so that success occurs if $y_t > d_1(y_c)$.

Conditional success probability (design stage). Given planning values $(\pi_t^\star, \pi_c^\star)$, the design-stage probability of meeting the success rule is

$$\text{CP}(\pi_t^\star, \pi_c^\star) = \sum_{y_c=0}^{n_c} \binom{n_c}{y_c} (\pi_c^\star)^{y_c} (1-\pi_c^\star)^{n_c - y_c} \sum_{y_t = d_1(y_c)+1}^{n_t} \binom{n_t}{y_t} (\pi_t^\star)^{y_t} (1-\pi_t^\star)^{n_t - y_t}. \tag{S4.6}$$

Design-stage operating characteristics (conditional on planning values). When evaluated at the null, $\pi_t^\star = \pi_c^\star = \pi$, (S4.6) yields the *conditional type I error rate*:

$$\text{T1E}_{\text{cond}} = \text{CP}(\pi, \pi).$$

When evaluated at an alternative with $\pi_t^\star > \pi_c^\star$, it yields the (design-stage) *conditional power*:

$$\text{Power}_{\text{cond}} = \text{CP}(\pi_t^\star, \pi_c^\star).$$

Probability of success (PoS, assurance). While conditional power (CP) assumes fixed true response rates (π_t, π_c), the probability of success averages CP over the design-stage priors for these parameters. For example, we use $\pi_t \sim \text{Beta}(1,1)$ and the robust MAP prior for π_c. Formally,

$$PoS = \int_0^1 \int_0^1 \text{CP}(\pi_t, \pi_c)\, p_T(\pi_t)\, p_{\text{rMAP}}(\pi_c)\, d\pi_t\, d\pi_c. \tag{S4.7}$$

Thus PoS represents the unconditional probability that the trial will declare success, integrating over current uncertainty in treatment and control response rates. In our implementation, (S4.6) and (S4.7) correspond to `oc2S()` and `pos2S()` with `decision2S(`γ`, 0, lower.tail=FALSE)`. The calculations use exact expressions, implemented in the `RBesT` package [6].

S5 Power comparison after type I error rate calibration

As a supplementary analysis, we assessed whether the apparent power gain from incorporating historical controls remained after restricting type I error rate. This is important because a procedure with a higher type I error rate can also show higher power simply because it uses a more permissive decision rule.

For test-then-pool, the separate analysis uses a one-sided Fisher's exact test. With a binary endpoint, the test is discrete and, without randomization at the rejection boundary, its attained type I error rate can be below the nominal level of 0.025. We therefore used the maximum empirical type I error rate of the separate analysis across the investigated prospective control response rates as the reference for each combination of between-trial heterogeneity and randomization ratio. The significance level of the final Fisher's exact test in each borrowing procedure was reduced until its maximum type I error rate did not exceed this reference. For robust MAP, the posterior probability threshold for declaring treatment benefit was increased from 0.975 until the maximum empirical type I error rate did not exceed 0.025. The borrowing model and prior specification were otherwise unchanged. In both frameworks, calibrated decision thresholds were determined under the null hypothesis and then applied under the alternative to estimate power. For each borrowing procedure s, the power difference at prospective control response rate π_c was defined as

$$\Delta_s(\pi_c) = \mathrm{E}_{H_1}\left[\phi_s^*(\mathcal{D}_0, \mathcal{H}_s)\right] - \mathrm{E}_{H_1}\left[\phi_{\mathrm{sep}}(\mathcal{D}_0)\right], \tag{S5.8}$$

where the expectations are evaluated under H_1 at the indicated value of π_c. Here, $\mathcal{H}_s$ denotes the historical data retained under selection mechanism s, ϕ_s^* denotes the corresponding borrowing decision rule using the recalibrated threshold, and ϕ_{sep} denotes the decision rule for the separate analysis. For test-then-pool, the calibrated threshold is denoted by α_s^*, whereas for robust MAP it is denoted by c_s^*, the posterior probability threshold for declaring treatment benefit. Table S7 reports the calibrated decision thresholds for the representative setting with $\tau = 0.30$ and 1:1 randomization.

Supplementary Table S7: Calibrated decision thresholds for the test-then-pool and robust MAP analyses for the setting with $\tau = 0.30$ and 1:1 randomization.

**Selection rule** s	**Test-then-pool** Fisher α_s^*	**Robust MAP** Posterior probability cutoff c_s^*
Drop-the-best	3.39×10^{-4}	0.995
Random selection	3.19×10^{-4}	0.994
Threshold selection	5.02×10^{-4}	0.995
Full selection	2.81×10^{-4}	0.994
Optimal power selection	7.81×10^{-4}	0.994

For test-then-pool, treatment benefit is declared when $p < \alpha_s^*$; a smaller value therefore represents a more stringent decision rule. For robust MAP, treatment benefit is declared when $\Pr(\pi_t - \pi_c > 0 \mid \mathcal{D}_0, \mathcal{H}_s) > c_s^*$. Here, a larger value represents a more stringent decision rule. The original thresholds were $\alpha = 0.025$ and $c = 0.975$, respectively.

Because the endpoint is binary, the attainable rejection probabilities are discrete, so the calibrated type I error rate may not have an exact match to the reference level. Across the investigated settings, calibration substantially reduced the apparent power advantage of borrowing (Supplementary Figure S17 – S18), consistent with previous work on fair power comparisons under type I error control by Kopp-Schneider et al.[7].

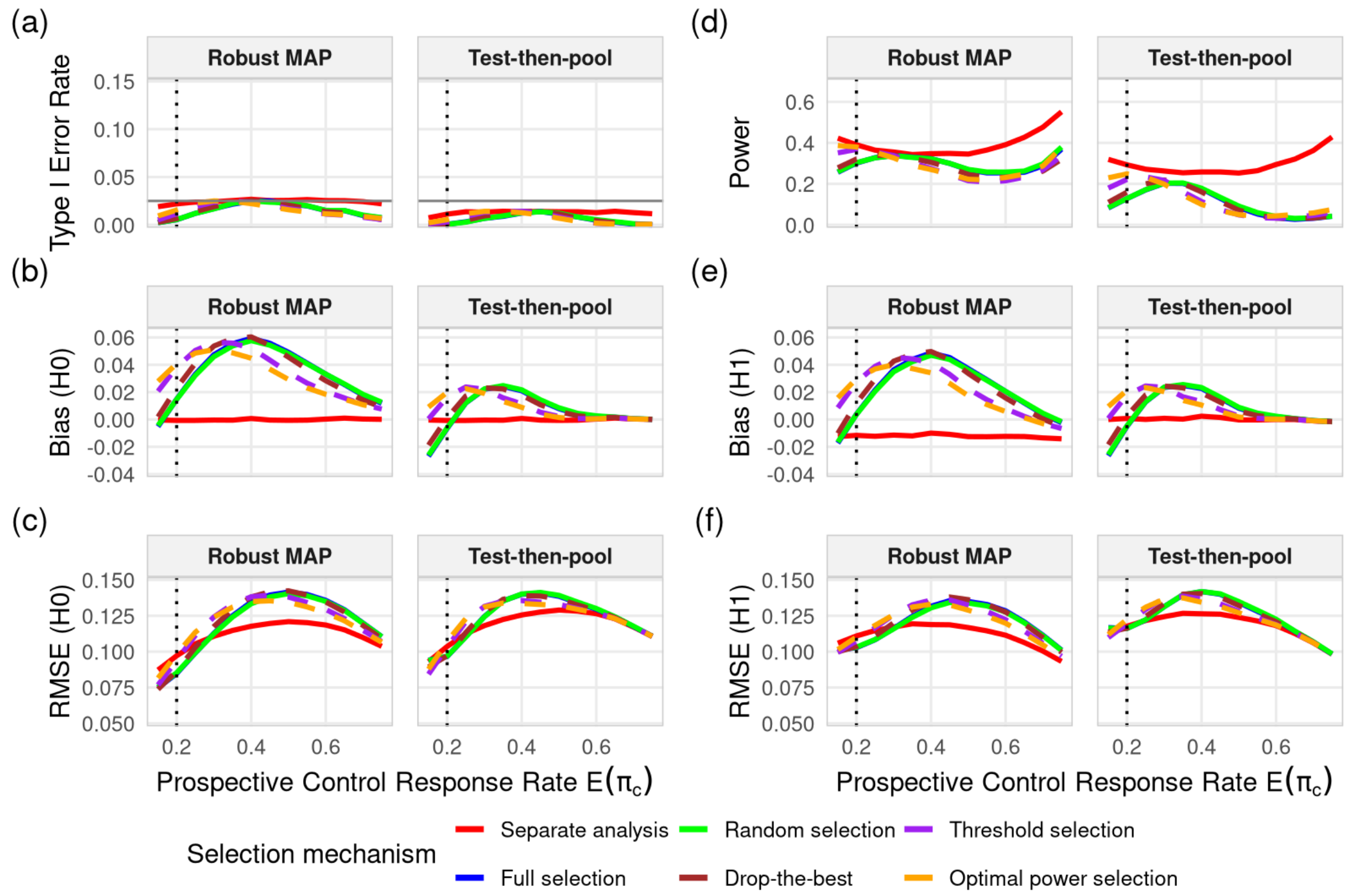

Supplementary Figure S17: Marginal operating characteristics of selection mechanisms with an initial set of $k = 8$ historical control trials each with a sample size of $n_{hc} = 30$ and assuming a moderate between-trial heterogeneity of $\tau = 0.30$, prospective trial total sample size $n_{\text{total}} = 60$. For the borrowing procedures, type I error rate and power were computed using calibrated decision thresholds that restricted the maximum type I error rate to the corresponding reference level. (a)–(c) H_0: type I error rate, bias, RMSE. (d)–(f) H_1: power, bias, RMSE. x-axis: prospective control response rate; the vertical line at $x = 0.20$ indicates exchangeability, where maximum amount of borrowing is appropriate; dashed lines denote outcome-dependent selections.

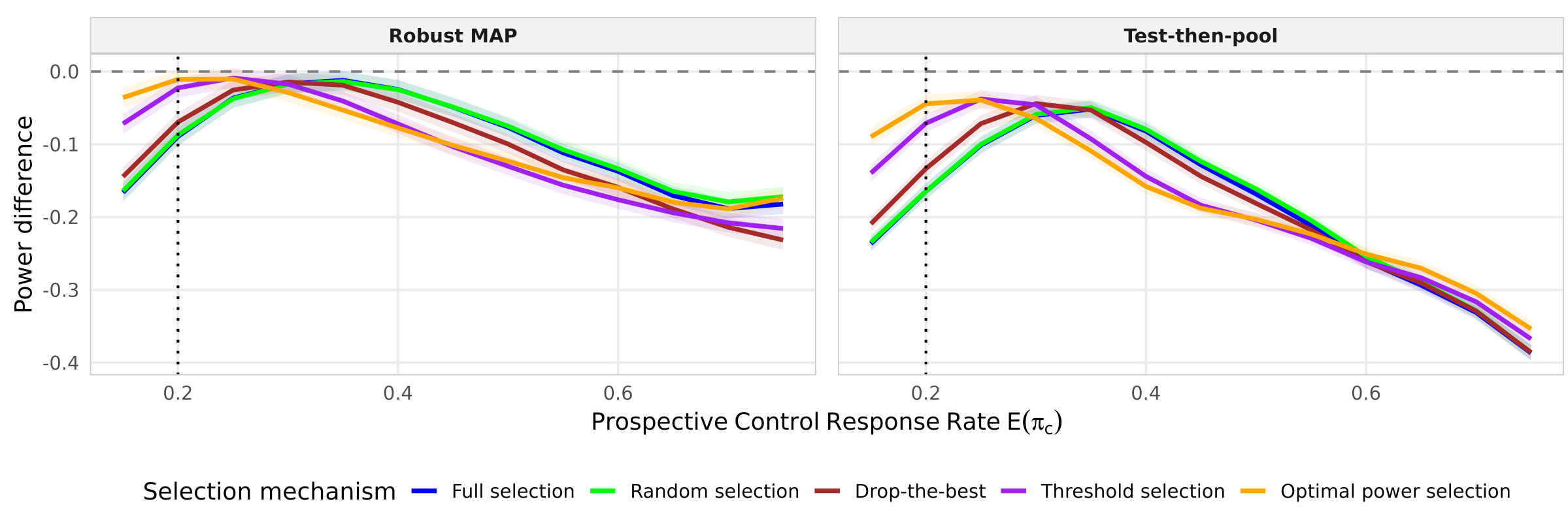

Supplementary Figure S18: Power difference $\Delta_s(\pi_c)$ between each borrowing procedure and the separate analysis for an initial set of $k = 8$ historical control trials each with sample size $n_{hc} = 30$, moderate between-trial heterogeneity $\tau = 0.30$, prospective trial total sample size $n_{\text{total}} = 60$, and 1:1 randomization. Shaded bands denote approximate 95% Monte Carlo intervals. The horizontal dashed line indicates equal power, and the vertical line at $x = 0.20$ indicates exchangeability between historical and prospective controls.

References

[1] S. R. Dalal and W. J. Hall. Approximating Priors by Mixtures of Natural Conjugate Priors. *Journal of the Royal Statistical Society Series B: Statistical Methodology*, 45(2):278–286, January 1983.

[2] T. Pham-Gia, N. Turkkan, and P. Eng. Bayesian analysis of the difference of two proportions. *Communications in Statistics - Theory and Methods*, 22(6):1755–1771, January 1993.

[3] Andrea Callegaro, Naveen Karkada, Emmanuel Aris, and Toufik Zahaf. Vaccine clinical trials with dynamic borrowing of historical controls: Two retrospective studies. *Pharmaceutical Statistics*, 22(3):475–491, May 2023.

[4] Beat Neuenschwander, Gorana Capkun-Niggli, Michael Branson, and David J Spiegelhalter. Summarizing historical information on controls in clinical trials. *Clinical Trials*, 7(1):5–18, February 2010.

[5] Dominique Baeten, Xenofon Baraliakos, Jürgen Braun, Joachim Sieper, Paul Emery, Désirée van der Heijde, Iain McInnes, Jacob M van Laar, Robert Landewé, Paul Wordsworth, Jürgen Wollenhaupt, Herbert Kellner, Jacqueline Paramarta, Jiawei Wei, Arndt Brachat, Stephan Bek, Didier Laurent, Yali Li, Ying A Wang, Arthur P Bertolino, Sandro Gsteiger, Andrew M Wright, and Wolfgang Hueber. Anti-interleukin-17A monoclonal antibody secukinumab in treatment of ankylosing spondylitis: a randomised, double-blind, placebo-controlled trial. *The Lancet*, 382(9906):1705–1713, November 2013.

[6] Sebastian Weber, Yue Li, John W. Seaman Iii, Tomoyuki Kakizume, and Heinz Schmidli. Applying Meta-Analytic-Predictive Priors with the *R* Bayesian Evidence Synthesis Tools. *Journal of Statistical Software*, 100(19), 2021.

[7] Annette Kopp-Schneider, Manuel Wiesenfarth, Leonhard Held, and Silvia Calderazzo. Simulating and reporting frequentist operating characteristics of clinical trials that borrow external information: Towards a fair comparison in case of one-arm and hybrid control two-arm trials. *Pharmaceutical Statistics*, 23(1):4–19, January 2024.
